# Multiple unbiased approaches identify oxidosqualene cyclase as the molecular target of a promising anti-leishmanial

**DOI:** 10.1016/j.chembiol.2021.02.008

**Published:** 2021-05-20

**Authors:** Luciana S. Paradela, Richard J. Wall, Sandra Carvalho, Giulia Chemi, Victoriano Corpas-Lopez, Eoin Moynihan, Davide Bello, Stephen Patterson, Maria Lucia S. Güther, Alan H. Fairlamb, Michael A.J. Ferguson, Fabio Zuccotto, Julio Martin, Ian H. Gilbert, Susan Wyllie

**Affiliations:** 1 Division of Biological Chemistry and Drug Discovery, Wellcome Centre for Anti-Infectives Research, School of Life Sciences, University of Dundee, Dow Street, Dundee DD1 5EH, UK; 2 Drug Discovery Unit, Wellcome Centre for Anti-Infectives Research, School of Life Sciences, University of Dundee, Dow Street, Dundee DD1 5EH, UK; 3 Global Health R&D, GlaxoSmithKline, Tres Cantos 28760, Spain

**Keywords:** oxidosqualene cyclase, Leishmania donovani, drug target, mechanism of action, lanosterol, neglected tropical disease, visceral leishmaniasis, drug discovery

## Abstract

Phenotypic screening identified a benzothiophene compound with activity against *Leishmania donovani*, the causative agent of visceral leishmaniasis. Using multiple orthogonal approaches, oxidosqualene cyclase (OSC), a key enzyme of sterol biosynthesis, was identified as the target of this racemic compound and its enantiomers. Whole genome sequencing and screening of a genome-wide overexpression library confirmed that *OSC* gene amplification is associated with resistance to compound **1**. Introduction of an ectopic copy of the *OSC* gene into wild-type cells reduced susceptibility to these compounds confirming the role of this enzyme in resistance. Biochemical analyses demonstrated the accumulation of the substrate of OSC and depletion of its product in compound (*S*)**-1**-treated-promastigotes and cell-free membrane preparations, respectively. Thermal proteome profiling confirmed that compound (*S*)**-1** binds directly to OSC. Finally, modeling and docking studies identified key interactions between compound (*S*)-**1** and the *Ld*OSC active site. Strategies to improve the potency for this promising anti-leishmanial are proposed.

## Highlights

• Genetics and chemo-proteomics identify the target of a promising anti-leishmanial
• Biochemical assays confirm the direct inhibition of oxidosqualene cyclase in cells
• Docking and modeling studies identify key interactions between compound and target
• Strategies to improve the potency of this benzothiophene are proposed

## Graphical abstract

**Figure:**
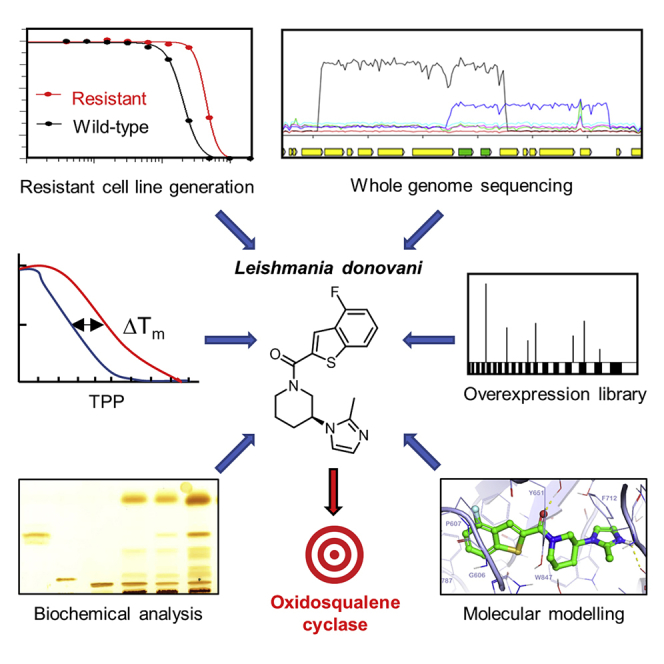

## Teaser

Paradela et al. investigated the mechanism of action of a promising anti-leishmanial. Using genetics, chemo-proteomics, and biochemical approaches, the target of this benzothiophene was confirmed as oxidosqualene cyclase, a key enzyme of sterol biosynthesis. Docking and modeling identified key interactions between this compound and the active site of this enzyme.

## Introduction

The protozoan parasites *Trypanosoma cruzi*, *Trypanosoma brucei,* and *Leishmania* spp. are the causative agents of the vector-borne diseases African sleeping sickness, Chagas disease, and the leishmaniases. Collectively, these diseases are responsible for more than 50,000 fatalities annually and the loss of more than 4,600,000 disease-adjusted life years (www.who.int/leishmaniasis/burden). Trypanosomatid diseases are principally diseases of poverty and inflict an enormous economic burden on some of the poorest countries on earth. Over the past 5 years, treatment options for African sleeping sickness have improved immeasurably with the introduction of nifurtimox-eflornithine combination therapy (NECT) to replace the more toxic organo-arsenical, melarsoprol (Priotto et al., 2009) and the licensing of the oral drug fexinidazole as an alternative to more complex parenteral regimens such as NECT (Pelfrene et al., 2019). Improved therapeutics, alongside robust surveillance screening programs and vector control measures, has significantly reduced cases of African sleeping sickness, raising hopes that this disease may be eliminated as a public health problem in the near future (Barrett, 2018). Unfortunately, new drugs for Chagas disease and leishmaniasis have been more difficult to develop. Current drugs suffer from a range of serious problems, including severe toxicity (Aldasoro et al., 2018; Soto and Soto, 2006), emerging drug resistance (Croft et al., 2006; Mueller et al., 2007), and poor efficacy (Jackson et al., 2010; Yun et al., 2009). New drugs that are safer, efficacious, and suitable for use in resource-poor settings are urgently required for the treatment of these neglected tropical diseases.

The principal goals of anti-trypanosomal drug discovery programs are to develop novel therapeutics that demonstrate improved efficacy with minimal host toxicity, are suitable for single-dose oral administration, and have the potential for use in a future combination therapy. However, progress has been hindered by the lack of robustly validated drug targets in *T. cruzi* and *Leishmania donovani* or *Leishmania infantum*, causative agents for Chagas disease and visceral leishmaniasis, respectively. As a consequence, almost all compounds currently in drug development pipelines for both diseases evolved from primary hits identified through whole-cell (phenotypic) screening of compounds (www.dndi.org) (Don and Ioset, 2014). Phenotypic approaches, although effective, do not provide information regarding the mechanism(s) of action (MoA) or specific molecular target(s) of active compounds. This information can be invaluable in the development of these compounds into selective and potent anti-parasitic agents, by addressing issues such as poor pharmacokinetic properties and host toxicity. MoA studies can also play a pivotal role in the management of drug discovery portfolios. For instance, compounds that act via mechanisms previously confirmed as unsuitable for drug development can be efficiently de-prioritized (Riley et al., 2015; Wall et al., 2018). Compound series found to inhibit the same molecular target(s) can be prioritized and rationalized, thus mitigating against the overpopulation of pipelines with compounds acting via the same mechanism (Wall et al., 2020). Furthermore, knowledge of the target pathway can be of potential value in optimizing drug combination therapy.

Here, we use orthogonal genetic, molecular, and biochemical approaches to determine the MoA of a benzothiophene compound demonstrating promising anti-leishmanial activity. Our comprehensive studies reveal that this compound specifically targets oxidosqualene cyclase (OSC), a key enzyme in the sterol biosynthetic pathway of these parasites. The implications of developing compounds with this MoA as anti-leishmanials of the future is discussed.

## Results

### Compound selection

**Figure 1:**
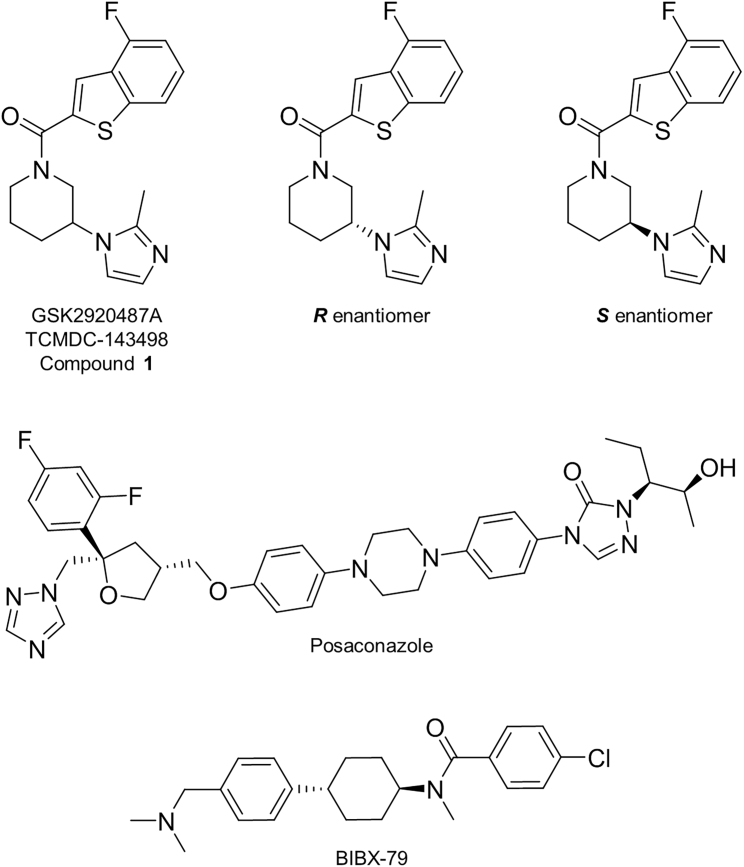
Chemical structures

GSK's Kinetoboxes are three open-access compound collections assembled following high-throughput phenotypic screening of 1.8M compounds against *T. brucei*, *L. donovani* and *T. cruzi* (Peña et al., 2015)*.* TCMDC-143498 (compound **1,** Figure 1), also known as GSK2920487A, is included in the *T. cruzi* box, having demonstrated promising activity against the intracellular stage of the parasite (half maximal effective concentration [EC_50_] value of 0.8 μM). Interestingly, this compound was also moderately active against *L. donovani* axenic amastigotes and promastigotes (EC_50_ values of 20 μM and 0.5 μM, respectively) while demonstrating limited activity against mammalian cell lines (HepG2) (Table 1). The principal aim of our current study was to gain an understanding of the mechanism of action and/or molecular target(s) of compound **1** to facilitate the development of more potent and selective versions of this compound. Our studies focused on using multiple unbiased approaches to determine the MoA of this compound in *L. donovani*.

**Table 1:** Collated EC_50_ values for compound 1 and related analogues

Compound	EC_50_ values, μM			
	Promastigotes	Axenic amastigotes	Intramacrophage	HepG2
Compound **1**	0.5 ± 0.02	20 ± 4	39 ± 8	>50
(*R*)-**1**	2.6 ± 0.1	>50	>50	>50
(*S*)-**1**	0.4 ± 0.01	17 ± 3	31 ± 7	>50
BIBX-79	0.5 ± 0.01	>50	ND	5 ± 0.4

EC_50_ values represent the weighted mean ± standard deviation of at least two biological replicates (n ≥ 2) with each biological replicate comprised of three technical replicates. The exception is intramacrophage amastigotes EC_50_ values, where data represent the mean ± standard deviation of two technical replicates and representative of two biological replicates. ND, not determined.

### Resistance generation followed by whole genome sequencing

**Figure 2:**
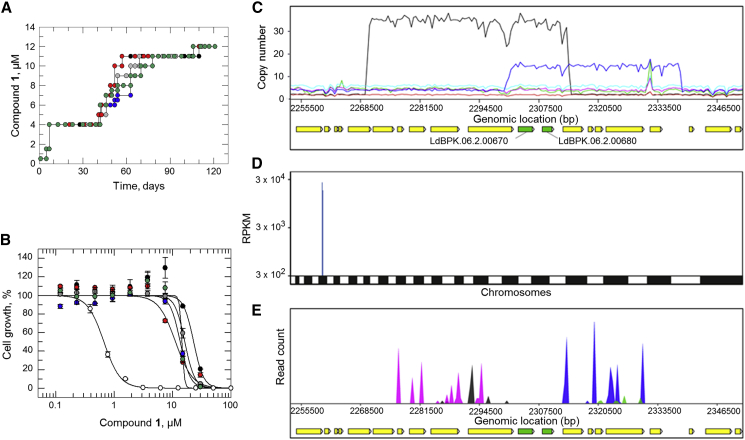
Target deconvolution studies with compound 1 (A) Schematic representation of the generation of compound **1**-resistant cell lines in *L. donovani*. Each passage of cells in culture (circles, lines 1-V) is indicated with cell lines I-V indicated in black, green, gray, blue, and red, respectively. (B) EC_50_ values for compound **1** were determined for WT (white circles) and cloned resistant cell lines I–V (black, gray, blue, red, and green circles, respectively). These curves are the nonlinear fits of data using a two-parameter EC_50_ equation provided by GraFit. An EC_50_ value of 0.7 ± 0.01 μM was determined for compound **1** against WT promastigotes. EC_50_ values for resistant clones I–V were 23 ± 4, 16 ± 0.3, 13 ± 1, 11 ± 1, and 14 ± 6 μM, respectively. These EC_50_ curves and values are from one biological replicate, composed of two technical replicates. Collated datasets reporting the weighted mean ± SD of multiple biological replicates are summarized in Table S3. (C) Copy number variations in resistant clones relative to WT. Amplification of chromosome 6 (or fragments) are evident in all resistant clones. Resistant clones are indicated as follows: RES I (blue), RES II (black), RES III (green), RES IV (pink), and RES V (cyan); WT clone is shown in red. *OSC* (LdBPK.06.2.000670) and *HP* (LdBPK._006.2.000680) green bars; other genes, yellow bars. (D) Genome-wide map indicating cosmid library hits from screening of compound **1**. A single primary hit was identified, indicated in blue. (E) Focus on primary “hit” on chromosome 6. *OSC* and *HP* genes shown as green bars; other genes, as yellow bars. The blue/pink and black/green peaks indicate independent cosmid inserts in different orientations.

As a first step toward identifying the molecular target(s) of compound **1**, *L. donovani* promastigote cell lines resistant to this benzothiophene were selected. Starting at 0.5 μM (1× EC_50_), five independent clonal lines of compound-susceptible parasites were exposed to stepwise increasing levels of compound **1** for a total of 120 days, until they were routinely growing at concentrations equivalent to >20× the established EC_50_ value (12 μM) (Figure 2A). The five independently generated resistant cell lines were cloned by limiting dilution and clones were assessed for susceptibility to compound **1**. The resulting clones were between 18- and 51-fold less sensitive to compound **1** than the wild-type (WT) parental clone (Figure 2B and Table S3). In each case, the resistance demonstrated by each clone was stable over at least 10 passages in culture in the absence of compound.

Genomic DNA recovered from the five resistant clones was analyzed by whole genome sequencing (WGS). Notably, copy number variant analysis revealed that all five resistant clones maintained amplified fragments of, or indeed entire copies of, chromosome 6. Resistant lines RES I, RES III, and RES IV had two additional copies of chromosome 6 compared with WT, while RES V maintained three additional copies (Figure 2C). RES I also maintained an additional 13 copies of a 30.9 kb region of chromosome 6. Furthermore, RES II resistant parasites had amplified a 44.3-kb fragment of chromosome 6, maintaining more than 34 extra copies of this chromosomal fragment compared with WT, drug-sensitive cells. Rationalizing the regions of chromosome 6 amplified in resistant clones indicates that a fragment encoding two complete gene-coding sequences is common to all. This coincident chromosomal fragment encodes oxidosqualene cyclase (*OSC*, LdBPK.06.2.000670) and a hypothetical protein (*HP*, LdBPK.06.2.000680).

A total of six single nucleotide polymorphisms (SNPs) encoding amino acid changes, relative to our parental clone, were identified in the genomes of compound **1**-resistant parasites (Table S4); however, no SNP was common to all cell lines. Notably, in RES V, a single heterozygous SNP was identified in the gene encoding OSC. This SNP encodes a Cys to Phe mutation at position 778 in this enzyme, a key component of the sterol biosynthetic pathway of *L. donovani*.

### Screening of compound 1 against an *L. donovani* genome-wide overexpression library

As a parallel unbiased approach to investigate MoA, compound **1** was also screened against our genome-wide cosmid-based overexpression library (Corpas-Lopez et al., 2019). The principle behind this gain-of-function approach is that overexpression of a drug target can result in resistance to the corresponding drug by increasing the pool of functional protein or by reducing free drug through binding. *L. donovani* promastigotes were transfected with a pooled population of cosmids containing genomic DNA fragments of between 35 and 45 kb. The final transfected library provides a >15-fold genome coverage with 99% of genes represented. The library was selected with 1 μM (2× EC_50_) compound **1** for 5 days and for a further 4 days at 2 μM. Following compound selection, cosmids maintained by the “resistant” parasite population were harvested and analyzed by next-generation sequencing. Mapping of overexpressed inserts to an assembled *L. donovani* genome revealed that 87% of all mapped reads aligned to a 54.5-kb region on chromosome 6 (Figures 2D and 2E). This region is composed of 10 genes (Table S5); however, only two genes were flanked by all the opposing barcodes: LdBPK.06.2.000670 encoding OSC and LdBPK.06.2.000680 encoding HP. Collectively, these data support our WGS analysis and the hypothesis that one of these proteins may be the molecular target of compound **1** or a key resistance determinant.

### Target validation

**Figure 3:**
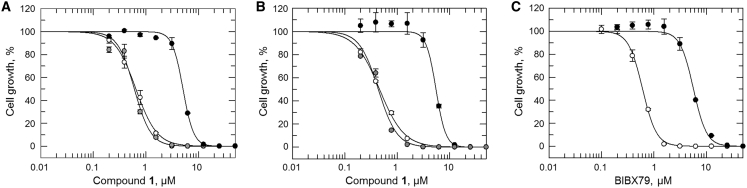
Validation of OSC as the molecular target of compound 1 (A) Dose–response curves for WT (white), OSC-overexpressing (black), and HP-overexpressing (gray) clones treated with compound **1**. EC_50_ values of 0.6 ± 0.01, 5 ± 0.1, and 0.6 ± 0.03 μM were determined for WT, OSC-overexpressing, and HP-overexpressing promastigotes, respectively. (B) EC_50_ values for WT (white), OSC-overexpressing (black), and OSC^C778F^-overexpressing (gray) promastigotes treated with compound **1** were 0.5 ± 0.01, 6 ± 0.3, and 0.4 ± 0.02 μM, respectively. (C) EC_50_ values for WT (white) and OSC-overexpressing cells (black) treated with BIBX-79 were 0.6 ± 0.01 and 6 ± 0.2, respectively. All EC_50_ curves and values are from one biological replicate, composed of two technical replicates. Collated datasets reporting the weighted mean ± SD of multiple biological replicates are summarized in Table S3.

To interrogate the potential role(s) of each protein in the MoA of compound **1**, cell lines overexpressing both putative targets were generated. Elevated levels of HP and OSC in these transgenic promastigotes, compared with WT, were confirmed by label-free MS quantification (Figure S1). While overexpression of HP had little or no effect on the potency of compound **1** (Figure 3A), cells overexpressing OSC were ~10-fold less sensitive to compound **1** in comparison to WT. These data identify OSC overexpression as the driver of compound **1**-resistance and identify this enzyme as the likely target of this benzothiophene. Next, we investigated the role of the OSC (Cys778Phe) mutation identified in RES V in resistance (Figure 3B). Promastigotes overexpressing the mutated version of this cyclase demonstrated the same level of susceptibility to compound **1** as WT. The fact that cells bearing elevated levels of this mutated enzyme remain susceptible to compound **1** not only rules out this mutation as playing any role in resistance, but also suggests that the mutated version of this enzyme may be nonfunctional.

**Figure 4:**
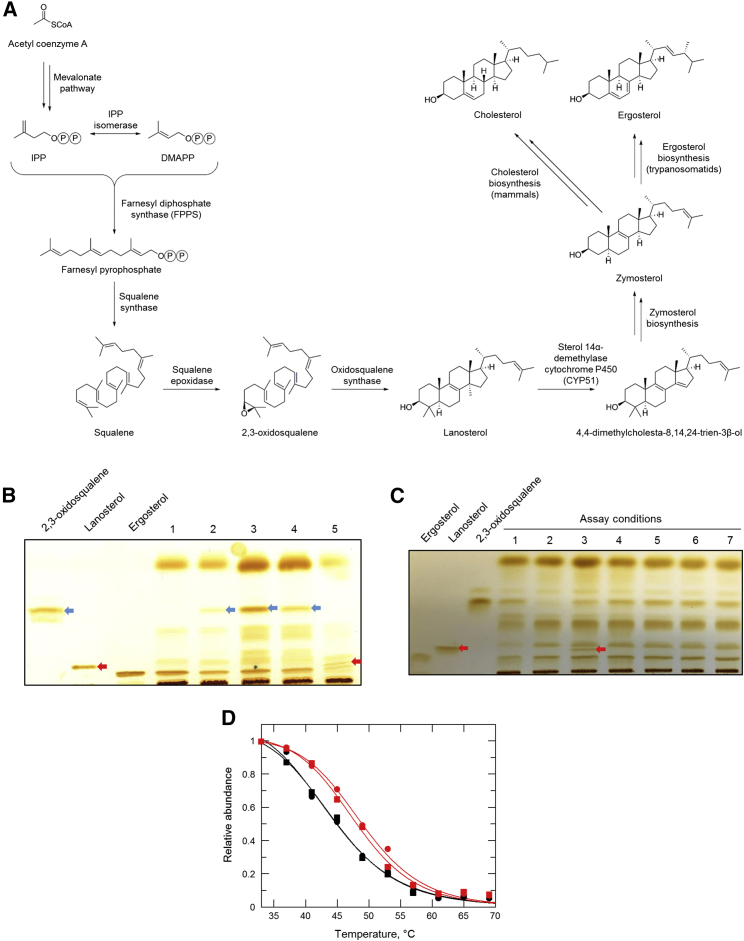
Effect of compound 1 on the sterol biosynthesis in *L. donovani* (A) Sterol biosynthetic pathway of *L. donovani*. (B) Sterols extracted from WT promastigotes (lane 1); promastigotes treated with compound **1** (racemate) (lane 2), compound (*S*)-**1** (lane 3), BIBX-79 (lane 4), and posaconazole (lane 5) for 96 h and analyzed by normal phase silica TLC. Ergosterol, lanosterol, and 2,3-oxidosqualene standards (1 mg·mL^−1^) were run in parallel and the unsaturated double bonds of separated lipids were stained with iodine vapor. Bands representing 2,3-oxidosqualene and lanosterol are indicated by blue and red arrows, respectively. (C) The activity of *L. donovani* OSC was monitored in cell-free membrane preparations by TLC. Crude membrane preparations were treated as follows: 2,3-oxidosqualene (OSC substrate) added and sterols immediately extracted (lane 1); no additions and sterols immediately extracted (lane 2); 2,3-oxidosqualene added, incubated at 37°C for 24 h and sterols extracted (lane 3); 2,3-oxidosqualene and compound (*S*)**-1** (equivalent to 1× EC_50_ value) added, incubated at 37°C for 24 h and sterols extracted (lane 4); 2,3-oxidosqualene and compound (*S*)**-1** (equivalent to 3× EC_50_ value) added, incubated at 37°C for 24 h and sterols extracted (lane 5); 2,3-oxidosqualene and compound (*S*)**-1** (equivalent to 1× EC_50_ value) added, preparation incubated at 37°C for 24 h and sterols extracted (lane 6); 2,3-oxidosqualene and BIBX-79 (equivalent to 3× EC_50_ value) added, incubated at 37°C for 24 h and sterols extracted (lane 7). Bands representing lanosterol (OSC product) are indicated by red arrows. (D) TPP melt curves for *L. donovani* OSC following incubation with compound (*S*)**-1** (red) or vehicle (0.1% DMSO, black). Data from technical replicates (circles and squares) are shown, and the mean shift in melting temperature (ΔT_m_) for OSC was 3.4°C.

OSC, also known as lanosterol synthase or ERG7, is a key enzyme in sterol biosynthesis, catalyzing the cyclization of 2,3-oxidosqualene to lanosterol (Figure 4A). A number of specific inhibitors of OSC have been described in the literature (Mark et al., 1996; Morand et al., 1997; Phillips et al., 2019) with a variety of different applications such as anti-fungal (Rose et al., 1996), anti-microbial (Hinshaw et al., 2003), and anti-cancer agents (Liang et al., 2014, 2016; Maione et al., 2015). Most notably, Buckner and colleagues (2001) have demonstrated that inhibitors of OSC can be potent inhibitors of *T. cruzi* growth *in vitro*. Consequently, we assessed the potency of an established OSC inhibitor, BIBX-79 (Mark et al., 1996), to evaluate potential cross-resistance against our transgenic and resistant cell lines. Indeed, OSC-overexpressing promastigotes were 10-fold less susceptible to BIBX-79 than WT (Figure 3C), demonstrating an equivalent level of resistance to that seen with compound **1**. Similarly, all compound **1**-resistant clones (RES I-V) were cross-resistant to BIBX-79 (Table S3). The cross-resistance profiles shared by compound **1** and BIBX-79 are entirely consistent with a shared mechanism of action and support OSC as the molecular target of both compounds in *L. donovani*.

### Stereochemistry of compound 1

Compound **1** has a chiral center at the 3-position of the piperidine ring, thus previously reported screening data associated with this benzothiozine has been obtained with a racemate (1:1 mixture of *R* and *S* enantiomers) (Peña et al., 2015). In order to profile the biological activity of the individual enantiomers, semi-preparative chiral chromatography was used to separate both enantiomers of compound **1**. The absolute stereochemistry of the enantiomer eluted first following chromatographic separation (retention time 11.9 min) was established by X-ray crystallography to be *R* (Figure S2 and Table S1). Therefore, the enantiomer eluted in peak 2 (retention time 15.5 min) is compound (*S*)-**1**. The enantiomeric excess values of the separated samples of (*R*) and (*S*) were measured by chiral HPLC and found to be >99.7% and 98.6%, respectively, indicating that both samples were of excellent optical purity. The potency of each enantiomer was then determined against the various developmental stages of *L. donovani* and also against the transgenic cell lines developed in the course of our studies (Tables 1 and S3). While both enantiomers appear to specifically target OSC, as evidenced by reduced sensitivity in cell lines overexpressing this enzyme, in all assays, the *S* enantiomer was found to be at least 10-fold more potent than (*R*)-**1**. Of particular note, (*S*)-**1** demonstrated activity against intramacrophage amastigotes (31 ± 7 μM, Table 1), while (*R*)-**1** was inactive at all concentrations tested.

### Evidence of target engagement

Our cumulative data provide compelling circumstantial evidence that OSC is the molecular target of compound **1** in *L. donovani*. Here, we sought to provide biochemical evidence of the inhibition of this enzyme by compound **1** and to investigate the broader effects on sterol biosynthesis. Thin-layer chromatography (TLC) was used to separate and then identify specific sterols in compound-treated and untreated promastigotes. Cells were treated with compound **1** (racemate), compound (*S*)-**1**, BIBX-79, or posaconazole for 96 h. Posaconazole targets sterol 14α-demethylase (CYP51), the enzyme immediately downstream of OSC that uses lanosterol as a substrate to produce 4,4-dimethylcholesta-8,14,24-trien-3β-ol. Neutral lipids were then extracted and separated by TLC alongside 2,3-oxidosqualene, lanosterol, and ergosterol standards (Figures 4B and S3). In keeping with our assertion that compound **1** and its enantiomers inhibit OSC, promastigotes treated with (*S*)-**1** were found to accumulate significant amounts of the substrate of this enzyme, 2,3-oxidosqualene, in comparison with untreated cells. Similarly, promastigotes treated with BIBX-79 were also found to accumulate this substrate. Levels of lanosterol, the product of OSC, would be reasonably expected to decrease in the presence of an OSC-specific inhibitor; however, lanosterol was below the limits of detection in the majority of our TLC studies, even in untreated parasites. This is likely due to the rapid turnover and generally low basal levels of lanosterol, a substrate for the zymosterol biosynthetic pathway (Figure 4A). Indeed, lanosterol is only visible, at very modest levels, in parasites treated with the CYP51-specific inhibitor posaconazole. In *T. cruzi*, CYP51 has been identified as a promiscuous drug target, with up to 80% of screening hits found to inhibit this enzyme (Riley et al., 2015). Previous studies report that compound **1** does not inhibit CYP51 in *in vitro* assays (Peña et al., 2015). The fact that the sterol signature of our posaconazole-treated promastigotes is distinct from that seen with compound **1**-treated parasites supports this observation.

Crude, cell-free membrane preparations were obtained by subjecting *L. donovani* promastigotes to nitrogen cavitation. Our aim was to use this cell-free, membrane system to focus solely on the reaction catalyzed by OSC, an established membrane protein. 2,3-oxidosqualene (OSC substrate) was added to these washed membranes prior to incubation at 37°C for 24 h. Sterols were then extracted, separated, and identified by TLC (Figure 4C). The production of lanosterol (OSC product) was clearly evident in assays supplemented with exogenous 2,3-oxidosqualene, confirming that OSC was active in these *L. donovani* membrane preparations. Lanosterol was undetectable in assays not supplemented with substrate. Most importantly, the production of lanosterol by membrane preparations supplemented with 2,3-oxidosqualene was ablated by the presence of the established OSC-inhibitor BIBX-79 and compound (*S*)-**1** at a range of concentrations. Collectively, these data provide direct, biochemical evidence that compound (*S*)-**1** inhibits the ergosterol biosynthetic pathway specifically at the 2,3-oxidosqualene to lanosterol step catalyzed by OSC.

Finally, thermal proteome profiling (TPP) was used as an approach to confirm the direct binding of compound (*S*)**-1** to OSC. TPP is based on the principle that binding of a drug to its protein target can significantly alter the thermal stability of that protein (Jafari et al., 2014). Briefly, *L. donovani* promastigotes were treated with compound (*S*)**-1** (10× established EC_50_ value) or DMSO vehicle. Lysates of treated promastigotes were then prepared and maintained in the continuous presence of compound or vehicle. Aliquots of each lysate were then incubated at designated temperatures (33–69°C), and for each temperature, insoluble (denatured) proteins were removed. The resulting soluble protein samples were reduced, alkylated, and digested with trypsin prior to derivatization with tandem mass tags. Pooled peptides were fractionated by HPLC and analyzed by LC/MS-MS prior to identification and quantitation. The melting points of each identified protein were then established. Full melt curves were established for 5,083 proteins, representing 68.3% coverage of the *L. donovani* proteome. The top 10 proteins demonstrating thermal shift in the presence of compound (*S*)**-1** and confirmed as legitimate “hits” by nonparametric analysis of response curves (NPARC), are summarized in Table S6. *Ld*OSC ranks as the fifth strongest “hit” in this unbiased, proteome-wide analysis, with individual melt curves revealing that the thermal stability of this enzyme increased by an average of 3.4°C in the presence of compound (*S*)**-1** (Figure 4D). These data confirm that compound (*S*)**-1** interacts directly with OSC and, in combination with our other studies, identifies *Ld*OSC as the molecular target of this benzothiophene compound.

### Molecular modeling

With the aim of understanding the molecular basis for the superior potency of compound (*S*)-**1** and identifying key interactions within the active site of *Ld*OSC, a homology model was generated using the structure of the human OSC orthologue complexed with lanosterol as a template (Thoma et al., 2004). The consensus *Ld-human* OSC sequence used to build the model was derived from a multisequence alignment of several OSCs and squalene cyclases (SC) from different organisms. The human and parasite enzymes share 40% sequence identity and 56% sequence similarity. This level of homology is well within the range required to generate a model of sufficient quality to support ligand binding studies. In general, the overall sequence similarity of this class of enzymes is relatively low. However, these enzymes are characterized by a number of functionally relevant QX_2-5_GXW consensus sequence motifs that are present in their C-termini (QW motifs) (Poralla et al., 1994). These motifs were used to guide and verify the sequence alignments. The *N*-terminus of *L. donovani* OSC (up to the QW7 motif) aligns poorly with OSC and SC sequences from other organisms. Thus, our modeling efforts focused on the C-terminus of the protein, which, with the exception of a 50-amino acid insertion between QW6 and QW7, aligns well with *h*OSC, and encompasses the lanosterol binding site. Indeed, the lanosterol binding site is particularly well conserved. Specifically, of the 31 amino acids within 5 Å of lanosterol, 23 residues are identical (74%) and five of the remaining eight share similar properties (19%) (Figure S4).

**Figure 5:**
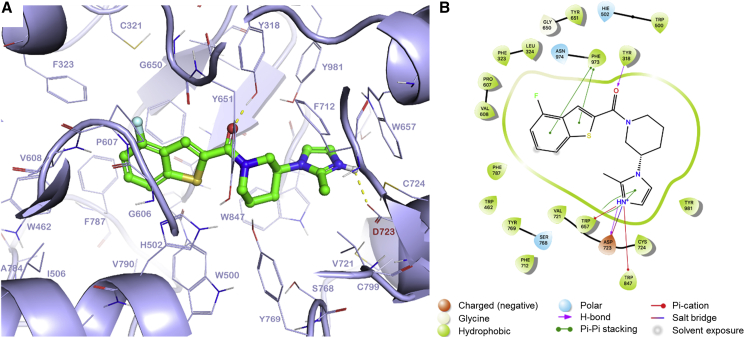
Docking of compound (*S*)-1 into a homology model of the *L. donovani* OSC active site (A) Induced-fit docking pose of compound (*S*)-**1**. The ligand is represented as green sticks. The hydrogen bonds between the ligand and Asp723 and Tyr318 are shown as a yellow dotted line. (B) 2D ligand interaction diagram based on the best-scoring docking pose for compound (*S*)-**1**.

The generated homology model was refined, optimized (see STAR methods), and used to investigate the binding modes of compounds (*S*)-1 and (*R*)-**1** by molecular docking. The *S* enantiomer generated the best docking score: −14.4 kcalꞏmol^−1^ compared with −12.7 kcal·mol^−1^ obtained for (*R*)-**1**. The top scoring poses for both enantiomers were used to evaluate changes in binding free energy (ΔG_bind_). In keeping with our crystallographic and potency data, the *S* enantiomer was confirmed as having a higher affinity for the active site of OSC with a ΔG_bind_ of −80.7 kcal·mol^−1^ compared with a value of −73.9 kcal·mol^−1^ for (*R*)-**1**. The most favorable binding pose for (*S*)-**1** is characterized by formation of a salt bridge with the carboxylate of the catalytic Asp723 through the positively charged nitrogen atom of the methylimidazole moiety (Figure 5). The imidazole moiety of compound **1** has a calculated pK_a_ value ranging between 7.8 and 8.6, suggesting that it is protonated at physiological pH. The model indicates that this positive charge is further stabilized by π-cation interactions with the aromatic systems of Trp657 and Trp847. The carbonyl oxygen is hydrogen bonded to the Tyr318 side chain hydroxyl group and the aromatic systems at the extremes of the molecule are involved in two π-stacking interactions: the first between the methylimidazole ring and the indole ring of Trp657 and second between the benzothiophene moiety and the aromatic ring in Phe973 (Figure 5). It should be noted that our model of OSC indicates that residue Cys778, mutated to Phe in our resistant cell lines RES V, is between 12 and 19 Å from the lanosterol binding site. The remoteness of this mutation from the binding site is consistent with it playing no direct role in resistance to compound **1**.

### Assessing the essentiality of OSC

**Figure 6:**
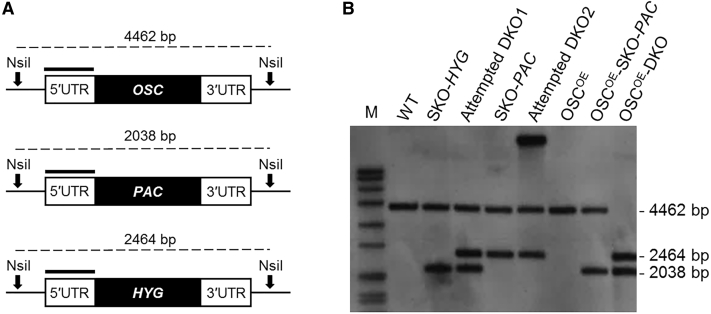
Assessing the essentiality of *OSC* in *L. donovani* promastigotes (A) Schematic representation of the *OSC* locus in *OSC* single-knockout (HYG) and (PAC) cells. Black bars represent the 5′ UTR region upstream of the open reading frame of OSC, which was used as a probe in Southern blot analysis. NsiI sites with expected fragment sizes are shown. (B) Southern blot analysis of NsiI-digested genomic DNA (~5 μg) from WT *L. donovani* (*Ld*BOB) cells, *OSC* single-knockout clones, attempted double knockout and a “rescued” DKO clone (OSC^OE^-DKO) . The DIG-labeled 5′ UTR of *OSC* was used as a probe. This Southern blot was stripped and re-probed using a DIG-labeled fragment of OSC as a probe (see Figure S5).

Previous studies have provided conflicting evidence regarding the essentiality of enzymes involved in sterol biosynthesis across different *Leishmania* species. For instance, sterol 14α-demethylase (CYP51) has been demonstrated as essential for survival in *L. donovani* (McCall et al., 2015), but not in *L. major* (Xu et al., 2014). Here, our aim was to assess the essentiality of OSC in *L. donovani* promastigotes for the first time using a classical gene replacement strategy (Figure 6A). In the first instance, a single copy of *OSC* was successfully replaced in WT parasites with either a puromycin (*PAC*, puromycin *N*-acetyltransferase) or hygromycin (*HYG*, hygromycin phosphotransferase) drug resistance gene via homologous recombination. Attempts were made to directly replace the second allelic copy of *OSC* in these single-knockout cells (SKO) with either *HYG* or *PAC* directly or in cells constitutively expressing an ectopic copy of *OSC*. Despite recovering more than 40 putative nulls, resistant to selection with both hygromycin and puromycin, all of these clones retained a genomic copy of *OSC.* Indeed, both allelic copies of *OSC* were only successfully replaced in promastigotes bearing an ectopic copy of the gene (Figures 6B and S5). Our failure to replace both the endogenous copies of *OSC*, except in the presence of an ectopic copy of the gene, strongly suggests that this enzyme is essential for the promastigote stage of *L. donovani*. The current absence of a robust and reliable inducible-expression system in *Leishmania,* compatible with use intramacrophage, precludes our investigation of OSC essentiality in the more relevant amastigote stage of the parasite.

## Discussion

Our current study highlights the power of using orthogonal approaches to elucidate mechanisms of compound action. Several lines of evidence presented here establish OSC, a key enzyme of sterol biosynthesis, as the primary target of compound **1** and its enantiomers. WGS and screening of our genome-wide overexpression library confirmed that elevated levels of OSC in *Leishmania* promastigotes are associated with resistance to compound **1**. The generation of OSC-overexpressing parasites, through introduction of an ectopic copy of the gene into WT cells, led to a precipitous drop in susceptibility to compound **1**, thus confirming the direct role of this enzyme in resistance. Cells treated with these compounds accumulate significant amounts of 2,3-oxidosqualene, the established substrate of OSC. *L. donovani* membrane preparations supplemented with 2,3-oxidosqualene produced lanosterol, while this activity was ablated by incubation with the established OSC-specific inhibitor BIBX-79, as well as in the presence of compound (*S*)**-1**. Finally, TPP was used as an unbiased approach to confirm that compound (*S*)**-1** directly and specifically interacts with this enzyme of sterol biosynthesis.

Sterols perform a vital function in maintaining the structural integrity of cellular membranes. OSC catalyzes the most complex step in the production of mature sterols, the cyclization of 2,3-oxidosqualene to form lanosterol. In higher eukaryotes (including humans), the predominant sterol is cholesterol; however, the membranes of kinetoplastid parasites, such as *Leishmania*, more closely resemble those of fungi in composition, with ergosterol and ergostane-based sterols most abundant (Haughan et al., 1995). Indeed, kinetoplastids are unable synthesize cholesterol *de novo* (Roberts et al., 2003) and the different sterol composition of parasite membranes compared with that of their mammalian hosts has long been considered exploitable for drug discovery. Of particular note, posaconazole, which targets CYP51, the enzyme immediately downstream of OSC, was recently assessed in phase II clinical trials for Chagas disease (Molina et al., 2014). Despite initially positive results, 92% of patients taking part in this trial relapsed in the subsequent 10 to 12 months. Buckner and colleagues (Buckner et al., 2001; Hinshaw et al., 2003) have a long-standing interest in developing OSC inhibitors as antimicrobials, particularly targeting *T. cruzi*, with the most promising demonstrating low nM activity against the mammalian (amastigote) stage of the parasite. Several studies have demonstrated that *Leishmania* are susceptible to antifungal inhibitors of sterol biosynthesis, such as terbinafine (squalene epoxidase inhibitor) (Bahamdan et al., 1997; Berman and Gallalee, 1987; Goad et al., 1985) and ketoconazole (CYP51 inhibitor) (Berman, 1981; Berman et al., 1984). Ketoconazole and fluconazole, another CYP51 inhibitor, have shown promise for the treatment of cutaneous leishmaniasis in clinical trials (Alrajhi et al., 2002; Saenz et al., 1990). Collectively, these studies demonstrate the suitability of sterol biosynthesis as a target for anti-leishmanial drug discovery.

Despite being unable to synthesize cholesterol, *T. brucei*, *Leishmania* spp. and *T. cruzi* have demonstrated an ability to scavenge this sterol from their hosts (Coppens et al., 1988; De Cicco et al., 2012; Pereira et al., 2011). Indeed, uptake of exogenous cholesterol, via endocytosis of low-density lipoprotein (LDL) particles, is essential for the survival of bloodstream-form *T. brucei* (Coppens et al., 1995). The fact that kinetoplastids can use host-derived cholesterol raises the possibility that this may provide a route for parasites to circumvent the effects of sterol biosynthesis inhibition and reduce the efficacy of drugs targeting this metabolic pathway. In support of this hypothesis, studies with *L. amazonensis* showed that promastigotes treated with a variety of sterol biosynthesis inhibitors responded by increasing endocytosis of LDL (Andrade-Neto et al., 2011). Furthermore, the potency of these inhibitors could be somewhat modulated by varying access to exogenous cholesterol. In our current study, we did not observe this phenomenon, finding instead that varying levels of fetal calf serum in culture medium (5%–20%), an exogenous source of cholesterol, had little or no effect on the potency of compound **1** against promastigotes (Figure S6). It should also be noted that scavenged cholesterol alone cannot satisfy promastigote and amastigote sterol requirements (Roberts et al., 2003), thus *de novo* synthesis of ergosterol-based sterols is essential.

Compound **1** and its enantiomers demonstrate a notable drop-off in potency against axenic and intramacrophage amastigotes, compared with promastigotes. Initially, we hypothesized that this reduced potency may be due to the sparing effect of cholesterol scavenged from the macrophage, as discussed above. However, this would not explain the drop in potency seen with amastigotes cultured axenically in the absence of a host cell. The imidazole moiety of compound **1** has a calculated pK_a_ value range of between 7.8 and 8.6, suggesting that a proportion of this benzothiophene will be protonated at physiological pH. Intramacrophage amastigotes reside within acidified parasitophorous vacuoles at pH 5.5 and axenic amastigotes are cultured in media that closely mimics this environment. At pH 5.5, the levels of protonated compound **1** will be considerably higher than those in promastigote cultures (pH 7.4). This positively charged moiety is likely to adversely affect compound **1** permeability and thus the increased levels of protonation in amastigote cultures may be at least partially responsible for the observed drop in potency. With this in mind, one strategy to improve the potency of compound (*S*)-**1** could be to replace the methylimidazole with a bioisostere with a lower pK_a_. For instance, the *h*OSC inhibitor BIBB515 has a dihydrooxazole moiety in this position (Lenhart et al., 2003) and a calculated pK_a_ value in the range of 5.44 to 4.72. Another potential strategy to improve potency is to exploit a hydrophobic channel within the active site of *Ld*OSC. Interestingly, both the terminal aliphatic chain of lanosterol and the *p*-bromo phenyl moiety of the *h*OSC inhibitor Ro 48-8071 extend in a hydrophobic channel that ultimately leads to the cell membrane (Thoma et al., 2004) (Figure S7). Based on our docking studies, positions 6 and 7 of the benzothiophene ring provide the right vector to exploit this channel and this has the potential to increase the potency of these inhibitors. Our intention is to investigate hydrophobic substituents to occupy this area.

In summary, these data confirm that compound **1** and its enantiomers specifically target OSC in *L. donovani*. We establish OSC as genetically essential for the survival of these parasites. Modeling and docking studies identify key interactions made between compound (*S*)-**1** and the OSC active site. In addition, we outline potential strategies to improve potency. Future studies should focus on evolving compounds within this series to achieve sub-μM potency against the mammalian stages of *L. donovani* prior to assessing the most promising compounds *in vivo*. It should be noted that compound **1** also demonstrates promising potency against intracellular *T. cruzi* (EC_50_ in the range 0.6–1 μM [Peña et al., 2015]). The failure of clinical trials with posaconazole for Chagas disease have left some in drug discovery reticent to pursue inhibitors of sterol biosynthesis. Nevertheless, future studies should also investigate the potential of these OSC-specific inhibitors for the treatment of American trypanosomiasis.

## Significance

**Visceral leishmaniasis (kala-azar) is a serious vector-borne disease afflicting people, particularly in parts of Asia, Africa, and Latin America. There are approximately 300,000 new cases and an estimated 20,000 deaths each year, making it the world's second biggest parasitic killer after malaria. In 95% of cases, death can be prevented by timely and appropriate drug therapy; however, current treatments are far from ideal. Clinically used anti-leishmanials suffer from a number of serious issues, including the need for hospitalization, prolonged therapy, parenteral administration, high cost, variable efficacy, severe toxic side effects, and resistance. Thus, there is a pressing need for better, safer efficacious drugs that are fit-for-purpose in resource-poor settings. Unfortunately, anti-leishmanial drug discovery has been hindered by a lack of robustly validated drug targets in these parasites. This has limited target-focused screening programs and has increased reliance on phenotypic screening of parasites to identify start points for drug discovery. This approach has proved effective, however, a lack of information regarding the mechanism(s) of action or specific molecular target(s) of these active compounds can prove a barrier to the optimization of these early lead compounds. Here, we used multiple, unbiased approaches to identify the molecular target of a promising phenotypic hit as OSC, a key enzyme in sterol biosynthesis. Identifying the target of this benzothiophene enabled structure-focused strategies to improve potency to be proposed. Furthermore, this knowledge can inform future drug combinations and be exploited for *de novo*, target-based drug discovery. This illustrates the great value of comprehensive mechanism of action studies as an integrated part of a drug discovery program.**

## STAR★Methods

### Key resources table

**Table:** 

REAGENT or RESOURCE		SOURCE	IDENTIFIER
**Chemicals, peptides, and recombinant proteins**			

Compound 1		GSK	TCMDC-85 143498
Compound 1		Enamine	Z1139335838
BIBX-79		Enamine	Z1768160684
Posaconazole		Sigma Aldrich	Cat# SML2287
Resazurin		Sigma Aldrich	Cat# R7017
G418 disulfate salt		Sigma Aldrich	Cat# A1720
Puromycin *N*-acetyltransferase		Invitrogen	Cat# ant-pr-1
Hygromycin 180 phosphotransferase		Invitrogen	Cat# ant-hg-1
Nourseothricin antibiotic		Jena Bioscience	Cat# AB-101
BamHI-HF restriction enzyme		New England Biolabs	Cat# R3136
SwaI restriction enzyme		New England Biolabs	Cat# R0604
SmaI restriction enzyme		New England Biolabs	Cat# R0141
Octyl β-D-glucopyranoside		Sigma Aldrich	Cat# O8001
Nα-Tosyl-L-lysine chloromethyl ketone hydrochloride		Sigma Aldrich	Cat# T7254
cOmplete™, Mini, EDTA-free Protease Inhibitor Cocktail		Sigma Aldrich	Cat# 11836170001
Bradford Reagent		Supelco	Cat# B6916
2,3-Oxidosqualene		Sigma Aldrich	Cat# 41043
Lanosterol		Sigma Aldrich	Cat# L5768
Ergosterol		Sigma Aldrich	Cat# PHR1512
Methanol		Sigma Aldrich	Cat# 34860
Chloroform		VWR Chemicals	Cat# 22711.260
Iodine		Sigma Aldrich	Cat# 207772
Heptane CHROMASOLV™		Fischer Scientific	Cat# 34873
Diethyl ether		Fischer Scientific	Cat# D/2400/21
Acetic acid (glacial)		VWR Chemicals	Cat# 20104.334P
Lysyl Endopeptidase®, Mass Spectrometry Grade		Alpha Labs (Wako)	Cat# 125-02543
Trichloroacetic acid solution 6.1 N		Sigma-Aldrich	Cat# T0699
HPTLC silica gel 60		Supelco	Cat# 1055470001

**Critical commercial assays**			

Human T Cell Nucleofector™ Kit	Lonza		Cat# VPA-1002
RNeasy Mini Kit	Qiagen		Cat# 74104
Luna® Universal One-Step RT-qPCR Kit	New England Biolabs		Cat# E3005
PCR DIG Probe Synthesis Kit	Roche		Cat# 11636090910
TMT10plex™ Isobaric Mass Tagging Kit	Thermo		Cat# 90111

**Deposited data**			

Sequencing of genome-wide cosmid library screening		European Nucleotide Archive (ENA)	PRJEB37256
Whole genome sequencing of resistant cell lines		European Nucleotide Archive (ENA)	PRJEB37435
Mass spectrometry data		Proteomics Identification Database (PRIDE)	PXD023780

X-ray structural data		Cambridge Crystallographic Data Centre (CCDC)	2027159
**Experimental models: cell lines**			

LdBOB cosmid-based genome-wide overexpression library		(Corpas-Lopez et al., 2019)	N/A
*Ld*BOB (MHOM/SD/62/1S-CL2D) RES I-V clones		This paper	N/A
OE-*LdOSC*-pIR1SAT		This paper	N/A
OE-*LdHP*-pIR1SAT		This paper	N/A
KO-*Ld*OSC-PAC cassette		This paper	N/A
KO-*Ld*OSC-HYG cassette		This paper	N/A

**Experimental models: organisms/strains**			

*Leishmania donovani Ld*BOB (MHOM/SD/62/1S-CL2D)		(Goyard et al., 2003)	

**Oligonucleotides**			

Summarised in Tables S1 and S2		This paper	University of Dundee oligonucleotide synthesis service
Primer SDM-*LdOSC*-F 5′-GACA CGCGGCCCGGCGTACTTCGA GCTGCTGGACTGTGCGG-3′		*In house*	University of Dundee oligonucleotide synthesis service
Primer SDM-*LdOSC*-R 5′- CCGCACAG TCCAGCAGCTCGAAGTACG CCGGGCCGCGTGTC-3′		*In house*	University of Dundee oligonucleotide synthesis service
Oxidosqualene cyclase (*Ld*OSC, LdBPK.06.2.000670)		Commercial synthesis (GeneArt, Invitrogen)	Custom synthesis
Hypothetical protein (*Ld*HP, LdBPK.06.2.000680)		Commercial synthesis (GeneArt, Invitrogen)	Custom synthesis

**Recombinant DNA**			

pIR1-SAT plasmid		Kindly provided by Professor Stephen Beverley Washington University	N/A

**Software and algorithms**			

GRAFIT version 5.0.4		Erithacus Software	http://www.erithacus.com/grafit/
Artemis genome browser		Wellcome Sanger	https://www.sanger.ac.uk/tool/artemis/
MaxQuant software		Mav Plank Institute	http://maxquant.org/
Schrödinger suite (2019-3 release)		Schrödinger, LLC, New York, NY, 2020	https://www.schrodinger.com/
Moka - Molecular Discovery		(Milletti et al., 2007)	https://www.moldiscovery.com/software/moka/
Bowtie2		(Langmead and Salzberg, 2012)	http://bowtie-bio.sourceforge.net/bowtie2/index.shtml
Samtools v1.9		(Li et al., 2009)	http://www.htslib.org/
BCFtools v1.9		(Li et al., 2009)	https://samtools.github.io/bcftools/
Artemis v16.0.0		Wellcome Sanger	https://www.sanger.ac.uk/tool/artemis/
RITseq.py - Python script for sequence mapping		(Glover et al., 2015)	Nature methods
Adobe Illustrator CS5.1		Adobe	https://www.adobe.com/
Adobe Photoshop CS5.1		Adobe	https://www.adobe.com/
Inkscape vector graphics Editor 0.48		Inkscape	https://inkscape.org/
Excel 365		Microsoft	https://www.office.com/

### Resource availability

#### Lead contact

Further information and requests for resources should be directed to the Lead Contact, Susan Wyllie (s.wyllie@dundee.ac.uk).

#### Materials availability

Materials and reagents are available from the authors upon reasonable request.

#### Data and code availability

Genomic datasets generated during this study are available at European Nucleotide Archive [https://www.ebi.ac.uk/ena] deposited under the accession numbers PRJEB37256 and PRJEB37435, respectively. Proteomics datasets generated during this study are available at Proteomics Identification Database [https://www.ebi.ac.uk/pride/] deposited under the accession number PXD023780. X-ray structural data generated during this study are available at the Cambridge Crystallographic Data Centre [https://www.ccdc.cam.ac.uk/] deposited under the accession number 2027159.

### Experimental model and subject details

#### Cell lines and culture conditions

A clonal *Leishmania donovani* cell line *Ld*BOB (derived from MHOM/SD/62/1S-CL2D) was grown as either promastigotes or axenic amastigotes in media-specific for each developmental stage, as previously described (Goyard et al., 2003). Axenic amastigotes were grown at 37°C in 5% CO_2_ and promastigotes were grown at 28°C.

### Method details

#### Chemistry

**Figure:**
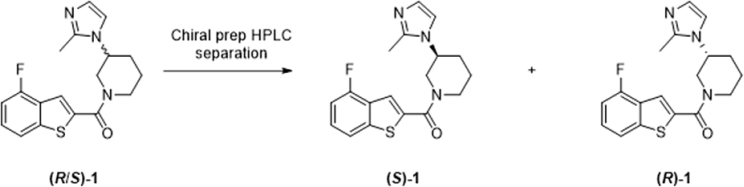

The enantiomers of (*R*/*S*)-1 were separated by chiral preparative-HPLC (performed by NEOMED Institute, Montréal). Full experimental details and analytical data for all compounds are shown below.

Absolute stereochemistry was determined by X-ray crystallography (Dr Alan Kennedy, University of Strathclyde, Glasgow). Briefly, a sample of the enantiomer that eluted first during HPLC purification was recrystallised by slow evaporation from heptane/toluene, and structural solution of the resultant crystals demonstrated that peak 1 was the *R* enantiomer. Crystallographic data were measured using a Bruker D8 Venture instrument. The structure was refined to convergence against F2 using all unique reflections and the program SHELXL-2014 as utilised within the WINGX GUI. All non-H atoms were refined anisotropically and H atoms were refined in riding modes, with the exception of H atoms in water molecules. These were refined independently and isotropically. Selected crystallographic data are presented in Table S1 and full structural details in cif format can be obtained from the Cambridge Crystallographic Data Centre (CCDC) via https://www.ccdc.cam.ac.uk/structures/. The database reference number is CCDC 2027159.

#### Chemistry - compound purification

^1^H-NMR, ^19^F-NMR, and 2D-NMR spectra were recorded on a Bruker Avance DPX 500 spectrometer (^1^H at 500.1 MHz, ^19^F at 470.5 MHz). Chemical shifts (δ) are expressed in ppm recorded using the residual solvent as the internal reference in all cases. Signal splitting patterns are described as singlet (s), doublet (d), triplet (t), quartet (q), multiplet (m), broad (br), or a combination thereof. LC-MS analyses were performed using an Agilent Technologies 1200 series HPLC connected to an Agilent Technologies 6130 quadrupole LC/MS connected to an Agilent diode array detector. High-resolution electrospray measurements were performed on a Bruker Daltonics MicrOTOF mass spectrometer.

##### (*R*/*S*)-(4-fluorobenzo[*b*]thiophen-2-yl)(3-(5-methyl-1*H*-imidazol-1-yl)piperidin-1-yl)methanone ((*R*/*S*)-1)

Purchased from Enamine (Product ID Z1139335838), off-white solid. MS (ES+): *m/z* (%) 344 (100) [M+H]^+^, 687 (9) [2M+H]^+^. HRMS (ES+): calcd. for C_18_H_19_F_1_N_3_O_1_S_1_ [M+H]^+^ 344.1227, found 344.1233 (1.5 ppm).

##### Chiral separation of (*R*/*S*)-(4-fluorobenzo[*b*]thiophen-2-yl)(3-(5-methyl-1*H*-imidazol-1-yl)piperidin-1-yl)methanone ((*R*/*S*)-1)

The individual enantiomers of **(*R*/*S*)-1** (88 mg) were separated on a ChiralPak IA column (10×250 mm, 5 μm) connected to a Minigram semi-preparative SFC system (mobile phase 25:75 MeOH +10 mM ammonium formate: CO_2_, 10 mL/min). The same HPLC system and conditions were used to determine the enantiomeric excess of each separated enantiomer.

(*R*)-(4-fluorobenzo[*b*]thiophen-2-yl)(3-(5-methyl-1*H*-imidazol-1-yl)piperidin-1-yl)methanone (**(*R*)-1**) (peak 1, retention time 11.9 min, e.e. >99.7%): off-white solid (29 mg). Note, on standing the material would turn into a semi-solid, likely due to formation of the hydrate. ^1^H-NMR (DMSO-*d*_6_): δ 7.89 (d, 1H, *J*=8.5 Hz, ArH), 7.77 (d, 1H, *J*=0.5 Hz, ArH), 7.51-7.46 (m, 1H, ArH), 7.26 (dd, 1H, *J*=10.5, 8.5 Hz, ArH), 7.20 (br s, 1H, ArH), 6.76 (br s, 1H, ArH), 4.50-4.00 (m, 3H), 3.4-3.0 (m, 2H), 2.29 (s, 3H, CH_3_), 2.10-2.04 (m, 1H, C*H*H), 2.03-1.95 (m, 1H, C*H*H), 1.89-1.82 (m, 1H, C*H*H), 1.76-1.67 (m, 1H, C*H*H). Note, many of the aliphatic peaks are broad probably due to restricted bond rotation. Note, residual formate from the HPLC purification was also observed. ^19^F{^1^H}-NMR (DMSO-*d*_6_): δ -117.4. MS (ES+): *m/z* (%) 179 (76) [M- 3-(5-methyl-1*H*-imidazol-1-yl)piperidine]^+^, 262 (39) [M- 5-methyl-1*H*-imidazole]^+^, 344 (100) [M+H]^+^.

(*S*)-(4-fluorobenzo[*b*]thiophen-2-yl)(3-(5-methyl-1*H*-imidazol-1-yl)piperidin-1-yl)methanone (**(*S*)-1**) (peak 2, retention time 15.5 min, enantiomeric excess. 98.6%): off-white solid (21 mg). Note, on standing the material would turn into a semi-solid, likely due to formation of the hydrate. ^1^H-NMR (DMSO-*d*_6_): δ 7.89 (d, 1H, *J*=8.5 Hz, ArH), 7.77 (d, 1H, *J*=0.5 Hz, ArH), 7.51-7.46 (m, 1H, ArH), 7.26 (dd, 1H, *J*=10.5, 8.5 Hz, ArH), 7.20 (br s, 1H, ArH), 6.76 (br s, 1H, ArH), 4.50-4.00 (m, 3H), 3.4-3.0 (m, 2H), 2.29 (s, 3H, CH_3_), 2.09-2.05 (m, 1H, C*H*H), 2.03-1.95 (m, 1H, C*H*H), 1.88-1.83 (m, 1H, C*H*H), 1.76-1.67 (m, 1H, C*H*H). Note, many of the aliphatic peaks are broad most likely due to restricted bond rotation. Note, residual formate from the HPLC purification was also observed. ^19^F{^1^H}-NMR (DMSO-*d*_6_): δ -117.4. MS (ES+): *m/z* (%) 179 (100) [M- 3-(5-methyl-1*H*-imidazol-1-yl)piperidine]^+^, 262 (21) [M- 5-methyl-1*H*-imidazole]^+^, 344 (15) [M+H]^+^. [α]^20^_D_ = +53.3 (c 1.00, CH_2_Cl_2_).

Chiral HPLC chromatograms and NMR spectra for (*R/S*)-1, (*R*)-1 and (*S*)-1 are available upon request.

#### Drug sensitivity assays

To examine the effects of compounds on parasite growth, promastigote and axenic amastigotes were seeded in 96-well plates at 5 × 10^4^ and 2 × 10^5^ parasites mL^-1^, respectively. Parasites were exposed to test compounds over a range of concentrations (two-fold serial dilutions). Cells were incubated for 72 h, after which resazurin was added to each well to a final concentration of 50 μM before measuring fluorescence (excitation of 528 nm and emission of 590 nm), after a further 2-3 h incubation. Data were processed using GRAFIT (version 5.0.4, Erithacus Software) and fitted to a 2-parameter equation to determine the effective concentration inhibiting growth by 50% (EC_50_):

$$y=\frac{100}{1+{\left(\frac{\left[I\right]}{EC_{50}}\right)}^{m}}$$

In this equation, [*I*] represents the inhibitor concentration, and m is the slope factor. Experiments were performed at least in three independent biological replicates for each parasite species with the data presented as the weighted mean ± standard deviation. Intra-macrophage drug sensitivity assays were determined, as previously described (Wyllie et al., 2012).

#### Cosmid library screening

The construction of our cosmid-based genome-wide overexpression library in *L. donovani* has been described in detail previously (Corpas-Lopez et al., 2019). Here, cosmid-containing *L. donovani* promastigotes were maintained at a minimum concentration of 3.33 x 10^5^ cells mL^-1^ (1.5 x 10^7^ cells in total) in the presence of 125 μg mL^-1^ G418. Compound **1** was added to the library at an initial concentration equivalent to 2x the established EC_50_ value. Cell densities were monitored daily and the library was sub-cultured before reaching 1 x 10^7^ mL^-1^, with addition of fresh test compound. Resistant cells were harvested and cosmid DNA isolated as described (Corpas-Lopez et al., 2019). After purification, cosmid DNA (30 μg in 100 μL Tris-buffer) was sequenced using an Illumina HiSeq platform (Beijing Genomics Institute). Sequence reads were aligned to the *L. donovani* BPKLV9 genome sequence (v39.0, tritrypdb.org) and *L. donovani* BPK282A1 genome sequence (v39, tritrypdb.org) using Bowtie2 software (Langmead and Salzberg, 2012) with the following condition: very-sensitive-local. The aligned files were then manipulated using SAMtools (Li et al., 2009) and a custom python script to identify reads with the following barcodes: 5’-GCGGCCGCTCTAGAACTAGT-3’ and 5’-CTCTTAAAAGCATCATGTCT-3’ (for fragments in sense direction) or 5’-ACTAGTTCTAGAGCGGCCGC-3’ and 5’-AGACATGATGCTTTTAAGAG-3’ (for fragments in anti-sense direction). Reads were then quantified using the Artemis genome browser (Carver et al., 2012) and Excel then analysed as previously described (Corpas-Lopez et al., 2019). All associated datasets have been deposited with the European Nucleotide Archive under the following accession number: PRJEB37256.

#### Resistance generation

Compound-resistant cell lines were generated by subculturing a clone of wild-type *L. donovani* in the continuous presence of test compounds. Starting at sublethal concentrations, drug concentrations in 5 independent cultures were increased in a stepwise manner. When parasites were able to survive and grow in concentrations of compound **1** equivalent to >20x the established EC_50_ value, the resulting cell lines were cloned by limiting dilution in the presence of compound. Five clones (RES I–V) were selected for further biological study.

#### Whole genome sequencing and analysis

Genomic DNA was isolated from WT and resistant clones using a standard alkaline lysis protocol. Whole genomic sequencing was performed using a HiSeq4000 next-generation sequencing platform (Beijing Genomics Institute, Hong Kong) and the resulting data was analysed as described previously (Wall et al., 2020), except the newly released LdBPKLV9 genome was also used as a reference. All associated datasets have been deposited with the European Nucleotide Archive under the following accession number: PRJEB37435.

#### Generation of overexpression and knockout constructs

Oxidosqualene cyclase (*LdOSC*, LdBPK.06.2.000670) and hypothetical protein (*LdHP*, LdBPK.06.2.000680) overexpression constructs were assembled by inserting synthetic versions of each gene (GeneArt, Invitrogen) into the pIR1SAT vector via a BamHI site. The resulting overexpression constructs (OE-*LdOSC* or OE-*LdHP*-pIR1SAT, respectively) were sequenced in-house to confirm their accuracy. Primers used in sequencing are summarised in Table S2.

*LdOSC* gene replacement cassettes were synthesised, comprising the selectable drug resistance genes puromycin *N*-acetyltransferase (*PAC*) or hygromycin phosphotransferase (*HYG*) flanked by the 470 bp immediately upstream and downstream of *LdOSC* gene. The accuracy of the resulting cassettes (KO-*LdOSC*-PAC and KO-*LdOSC*-*HYG*) were again verified by sequencing. Primers used in sequencing are summarised in Table S2.

#### Generation of LdBOB transgenic cell lines

Overexpression constructs were linearised with SwaI and knockout constructs were digested with SmaI prior to transfection. Mid-log-promastigotes (2 x 10^7^ cells in total) were transfected with 5 – 10 μg of overexpression or knockout constructs using the Human T-Cell Nucleofector kit and Amaxa Nucleofector electroporator (program V-033). Following transfection, cells were allowed to recover for 16-24 h, before the appropriated drug selection (nourseothricin 100 μg mL^−1^, hygromycin 50 μg mL^-1^ and puromycin 20 μg mL^-1^). Cloned cell lines were generated by limiting dilution, maintained in selective medium and removed from drug selection for one passage prior to experiments.

#### Southern blot analysis of transgenic cell lines

The ORF and 5′ UTR of *OSC* were amplified by PCR (using the primers listed in Table S2) with the PCR DIG Probe Synthesis kit (Roche). The resulting digoxigenin (DIG)-labelled products were used as probes and Southern-blot analysis was carried out as previously described (Wyllie et al., 2009).

#### Protein quantification

*L. donovani* promastigotes WT and transgenic cell lines confirmed as overexpressing OSC and HP were grown for 72 h in roller bottles, starting at an initial concentration of 1 × 10^5^ cells mL^−1^ (1.5 × 10^7^ cells in total). Mid-log phase promastigotes were washed with ice-cold PBS and harvested by centrifugation (1912 g, 15 min, 4°C). The cell pellets were resuspended in 8 mL of ice-cold lysis buffer (1 mM EDTA, 1 mM DTT, 100 μM TLCK, and 1× Roche EDTA-free cOmplete protease inhibitor cocktail in 50 mM potassium phosphate buffer, pH 7.4), submitted to 3 freeze−thaw cycles in a dry ice/ethanol bath to biologically inactivate the parasites and followed by cell disruption (Constant Systems, UK) at 30 kpsi. The resulting lysates were centrifuged (100,000 g, 20 min, 4°C), supernatants were collected, and the protein concentrations were determined using the Bio-Rad Protein Assay.

#### LC-MS/MS analysis

Analysis of peptides was performed on a Q Exactive™ plus, Mass Spectrometer (Thermo Scientific) coupled to a Dionex Ultimate 3000 RS (Thermo Scientific). The following buffers were used: Buffer A (0.1% formic acid in Milli-Q water (v/v)) and Buffer B (80% acetonitrile and 0.1% formic acid in Milli-Q water (v/v)). Samples (15 μL) were loaded at 10 μL min^-1^ onto a trap column (100 μm × 2 cm, PepMap nanoViper C18 column, 5 μm, 100 Å, Thermo Scientific) that had been pre-equilibrated with Buffer A (98%). The trap column was then washed for 5 min and switched in-line with a resolving C18 column (75 μm × 50 cm, PepMap RSLC C18 column, 2 μm, 100 Å; Thermo Scientific). Peptides were eluted from the column at a constant flow rate of 300 nL min^-1^ with a linear gradient of 2-35% Buffer B over 125 min, followed by 98% Buffer B for 127 min. The column was then washed with 98% Buffer B for 20 min prior to equilibration in 2% Buffer B for 17 min. The Q Exactive plus was used in data-dependent mode. The scan cycle comprised MS1 scan (m/z range from 335-1600, with a maximum ion injection time of 20 ms, a resolution of 70 000 and automatic gain control (AGC) value of 1 × 10^6^) followed by 15 sequential dependent MS2 scans (with an isolation window set to 1.4 Da, resolution at 17500, maximum ion injection time at 100 ms and AGC 2 × 10^5^). Stepped collision energy was set to 27 and fixed first mass to 100 m/z. The spectrum was acquired in centroid mode and unassigned charge states, charge states above 6, as well as singly charged species, were rejected. To ensure mass accuracy, the Q Exactive plus was calibrated on the day of analysis. LC-MS analysis was performed by the FingerPrints Proteomics Facility (University of Dundee).

#### Data analysis

MS data analysis was performed using MaxQuant software (http://maxquant.org/, version 1.6.2.6a). Carbamidomethyl (C), oxidation (M), acetyl (Protein N-term), deamidation (NQ) and Gln-> pyro-Glu were set as a variable modification. Proteins were identified by searching a protein sequence database containing *L. donovani* BPK282A1 annotated proteins (downloaded from TriTrypDB 46, http://www.tritrypdb.org). Label-free quantitation (LFQ) and “match between runs” features were enabled. Trypsin/P and LysC/P were selected as the digestive enzymes with two potential missed cleavages. The FDR threshold for peptides and proteins was 0.01. FTMS MS/MS mass tolerance was set to 10 ppm and ITMS MS/MS mass tolerance was 0.6 Da. Protein abundance was obtained from LFQ intensity values. LFQ intensities were calculated using at least 2 unique peptides. Data was visualised using Perseus 1.6.2.1 (https://maxquant.org/perseus/).

#### Site-directed mutagenesis

The custom-synthesised plasmid pMS containing *LdOSC* gene was used as a template for site-directed mutagenesis using the QuikChange II Site-Directed Mutagenesis Kit (Agilent), as per manufacturer's instructions. Complimentary primers were designed to generate a single nucleotide polymorphism (G2333T): SDM-*LdOSC*-F 5′-GACACGCGGCCCGGCGTACTTCGAGCTGCTGGACTGTGCGG-3′ and SDM-*LdOSC*-R 5′-CCGCACAGTCCAGCAGCTCGAAGTACGCCGGGCCGCGTGTC-3′. PCR conditions were as follows: 1 cycle of 30 s at 95°C; and 16 cycles of 30 s at 95°C, 1 min at 55°C and 5 min at 68°C. The accuracy of the mutagenesis was confirmed by sequencing.

#### Analysis of neutral lipids by thin-layer chromatography (TLC)

Promastigotes were incubated with compound **1,** compound (*S*)-**1,** BIBX-79 or posaconazole at concentrations equivalent to 3x their respective EC_50_ values. Depending on the level of compound treatment, the starting cell density of cultures varied between 1 × 10^3^ cells mL^−1^ and 8 × 10^5^ cells mL^−1^. After 96 h, 50 mL of each culture (mid-log) were washed 2× with phosphate-buffered saline (3000*g*, 5 min, RT). Resulting pellets were resuspended in 1:2:0.8 parts of chloroform:methanol:water (v/v/v), homogenised and left at room temperature overnight for extraction. The supernatant enriched with lipids was harvested following centrifugation (3000*g,* 20 min), transferred to a new tube and subjected to a second round of extraction. The lower phase containing the total pool of lipids was collected and the extract volume corresponding to 1 × 10^8^ cells was dried under nitrogen.

Extracted lipids were dissolved in 30 μL chloroform, spotted onto HPTLC silica gel 60 plates (Sigma) and allowed to migrate in heptane: ethyl ether: acetic acid (85:15:1, v/v/v). Ergosterol, lanosterol and 2,3-oxidosqualene standards (1 mg mL^-1^) were run in parallel. Following separation, the unsaturated double bonds of lipids were stained with iodine vapour.

#### OSC cell-free assay

Mid-log promastigotes were harvested by centrifugation (3000*g*, 10 min, RT) and washed once with ice-cold phosphate-buffered saline. Membrane-enriched lysates were prepared by nitrogen cavitation using a pre-chilled 45 mL Parr cell disruption vessel (406 PSI, 4°C), as described (Brown et al., 1996). Aliquots of membrane-enriched fractions (equivalent to 3 x10^8^ cells) were suspended in Buffer A (50 mM Hepes pH 7.4, 25 mM KCl, 5 mM MgCl_2_, 5 mM MnCl_2_, 0.1 mM TosLysCH_2_Cl, 1 μg/mL leupeptin) supplemented with 20% glycerol, flash-frozen in liquid nitrogen and stored at −80°C. Prior to assay, aliquots were defrosted on ice then diluted in Buffer A supplemented with 0.8% of octyl β-D-glucopyranoside to the final volume of 600 μL. Substrate (2,3-oxidosqualene, 6 μg) and/or test compounds (*S*)-1 and BIBX-79 were added at concentrations equivalent to 1, 3 or 10× their respective EC_50_ values. Following incubation for 24h at 37°C, lipids were extracted from each sample, spotted onto HPTLC silica gel and stained with iodine vapour, as previously described.

#### Homology modelling

The full amino acid sequence of *Ld*OSC (E9B8S7) was used to identify homologues through a BLAST homology search. The human OSC (*h*OSC: P48449) demonstrated 40% sequence identity and 58% similarity to the parasite enzyme; and was chosen as a suitable template for homology modelling. A 2.1Å crystal structure of *h*OSC complexed with lanosterol was used as the template structure (PDB ID: 1W6K). In this structure, a lanosterol molecule is fully enclosed within the enzyme active site. To improve the modelling of the enzyme binding site, lanosterol was also modelled into the active site of *Ld*OSC. A multi-sequence alignment of 10 squalene and oxidosqualene cyclases from different organisms was used to derive the *Ld*-human OSC sequence alignment used to build the homology model. The alignment was further manually curated to move an insertion from an α-helix into a loop region. The *LdOSC* model was generated and optimised using the protein prediction algorithm Prime and other tools available in the Schrödinger modelling platform (Schrödinger Release 2019-3: Schrödinger, LLC, New York, NY, 2020). After an initial restrained minimization using the Protein Preparation Wizard tool, non-template loops were further minimised using the Refine Loops tool. The hydrogen bonding network of the whole model was optimised reorienting the hydroxyl (Ser, Tyr), thiol (Cys) and the amide groups (Asn and Gln), as well as the imidazole rings (His). In addition, the predicted protonation states of His, Asp and Glu, and tautomeric states of His were also optimised. VSGB Solvation Model and the OPLS3e force field were used to minimise strain in the structure and to adjust the placement of various groups.

#### Lysate production for thermal proteome profiling (TPP)

*L. donovani* promastigotes (5 × 10^7^ parasites mL^-1^) were incubated in the presence of compound (*S*)-1 (equivalent to 10× the established EC_50_ value) or vehicle (0.1% (v/v) DMSO) for 3h at 28°C. Parasites were then harvested (1912 g, 15 min, 4°C), washed once with ice-cold phosphate-buffered saline and again harvested. The resulting cell pellets were resuspended in 8 mL of ice-cold lysis buffer (1 mM EDTA, 1 mM DTT, 100 μM TLCK, 0.8% octyl β-D-glucopyranoside and 1× Roche EDTA-free cOmplete protease inhibitor cocktail in 50 mM potassium phosphate buffer, pH 7.4) and then submitted to 3 freeze−thaw cycles in order to biologically inactivate the parasites. Cells were then lysed using the Constant Systems cell disruptor at 30 kpsi. The resulting lysates were centrifuged (100,000 g, 20 min, 4°C), supernatants collected, and protein concentrations determined, using the Bio-Rad Protein Assay. It should be noted that each step of this process was carried out in the constant presence of compound (*S*)-1 (10 × EC_50_) or DMSO, as appropriate.

#### TPP assays

Lysates were adjusted to a final concentration of 1 mg/mL with Lysis Buffer. Aliquots (2 x 2 mL) were incubated at room temperature for 30 min in the presence of compound 1 (*S*) (10x EC_50_ value) or vehicle (0.1% DMSO). Each 2 mL aliquot (drug and vehicle treated) was further divided into 10 x 100 μL aliquots in 0.5 mL Protein LoBind tubes (Eppendorf) and incubated at the following temperatures (33, 37, 41, 45, 49 , 53, 57, 61, 65 and 69°C) for 3 min, followed by incubation at RT for 3 min. Each sample was then placed on ice for 15 min, centrifuged (100,000 g, 20 min, 4°C), supernatants harvested, and protein concentrations determined.

#### TPP sample processing and analysis

All aspects of sample processing, fractionation by HPLC, LC-MS/MS, peptide and protein identification and quantitation were described previously (Corpas-Lopez et al., 2019).

#### TPP data analysis

TPP experiments were analysed using the TPP Package available in Bioconductor, as previously described (Corpas-Lopez et al., 2019; Franken et al., 2015). Briefly, raw protein abundance, calculated from normalized reporter ion intensities of all quantified proteins, were log transformed and scaled between 0 and 1 by subtracting the global minimum and normalizing to the abundance at the lowest temperature of each protein to yield fold changes. The melting curves were calculated using a sigmoidal fitting approach with the TPP R package. This fitting was used to determine the melting point (T_m_), defined as the temperature at which half of all proteins were denatured. The melting point differences (ΔT_m_) were calculated by subtracting the T_m_ values of treated and untreated sample. The sigmoidal melting curves were filtered according to the following criteria: melting curves must reach a relative abundance plateau <0.3 and the coefficient of determination (R2) must be >0.8. In addition, non-parametric analysis of response curves (NPARC) was performed (Childs et al., 2019). This procedure is based on non-parametric statistics, comparing two models (treated and control curves) by their goodness of fit, allowing the detection of treatment-induced changes in the absence of the melting point, the central parameter of the standard method. Proteins with a FDR-adjusted *p*-value <0.01 in one biological replicate are considered hits. The mass spectrometry proteomics data have been deposited to the ProteomeXchange Consortium via the PRIDE (Perez-Riverol et al., 2019) partner repository with the dataset identifier PXD023780.

#### Induced-fit docking

Low energy conformers for both enantiomers of compound **1** were generated using LigPrep in the Schrödinger platform. The protonation state of each enantiomer at pH 7.4 was defined by MoKa from the Molecular Discovery suite (Milletti et al., 2007). The prepared ligands were then docked into the binding pocket of the *Ld*OSC model using the Schrödinger induced-fit docking protocol that uses Glide, OPLS3e force field and Prime to minimize the poses in the binding site obtained for compound **1**. A 20 Å cubic box centred on the centroid of the lanosterol ligand was used to generate the docking grid. No distance or hydrogen bond constraints were applied. The default settings were modified to increase the conformational sampling of the aliphatic rings of each ligand by increasing the energy window to 2.5 kcal mol^−^^1^ and the non-planar conformation of the amide bonds were penalised. For the Prime Refinement options the residues to be refined were at 6.0 Å from the ligand poses and no other residues were selected. For the redocking options the Extra-Precision method was selected (XP).

#### Binding energies

The molecular mechanics energies, combined with the generalised Born and surface area continuum solvation (MM-GBSA), were calculated using the following equation in Prime from Schrödinger with the VSGB Solvation model and OPLS3e force field:

ΔG_bind_ = E_complex(minimised)_ - E_ligand(minimised)_ - E_receptor(minimised)_

The Minimise Sampling method was also applied.

#### pK_a_ calculations

A variety of different software packages including SciFinder (scifinder.cas.org), MoKa (https://www.moldiscovery.com/software/moka/) and Epik (https://www.schrodinger.com/epik) were used to calculate pK_a_ values for compounds within this study.

### Quantification and statistical analysis

All potency data were analysed in GraFit using their 2- parameter fit. Details of replicates and data analysis for each experiment can be found in the figure legends. Label-free quantitative proteomics experiments were analysed using MaxQuant software (http://maxquant.org/, version 1.6.2.6a). The FDR threshold for peptides and proteins was 0.01. FTMS MS/MS mass tolerance was set to 10 ppm and ITMS MS/MS mass tolerance was 0.6 Da. Protein abundance was obtained from LFQ intensity values. LFQ intensities were calculated using at least 2 unique peptides. Thermal proteome profiling experiments were analysed using the TPP Package available in Bioconductor. Melt curves were calculated using a sigmoidal fitting approach with the TPP R package. Non-parametric statistical analysis of response curves (NPARC) (Childs et al., 2019) involves the comparison two models (treated and control curves) by their goodness of fit, allowing the detection of treatment-induced changes in the absence of the melting point, the central parameter of the standard method. Proteins with a FDR-adjusted *p*-value <0.01 in one biological replicate were considered hits.

## Supplemental information

Document S1. Figures S1–S7 and Tables S1–S6

Document S2. Article plus Supplemental information
