# Supplementary material for: Multiple unbiased approaches identify oxidosqualene cyclase as the molecular target of a promising anti-leishmanial
Source: Cell Chem Biol. 2021 May 20;28(5):711–721.e8. doi: 10.1016/j.chembiol.2021.02.008 (PMC8153249; doi:10.1016/j.chembiol.2021.02.008)
Supplement: Document S1. Figures S1–S7 and Tables S1–S6 [file mmc1.pdf]

Cell Chemical Biology, Volume *28*

Supplemental information

Multiple unbiased approaches identify oxidosqualene cyclase as the molecular target of a promising anti-leishmanial

Luciana S. Paradela, Richard J. Wall, Sandra Carvalho, Giulia Chemi, Victoriano Corpas-Lopez, Eoin Moynihan, Davide Bello, Stephen Patterson, Maria Lucia S. Güther, Alan H. Fairlamb, Michael A.J. Ferguson, Fabio Zuccotto, Julio Martin, Ian H. Gilbert, and Susan Wyllie

Supplementary information

Table S1. Selected crystal data and structural refinement for peak 1 isolated from chiral separation of compound 1 (racemate). Related to Figure **1** and table **1**.

Identification code	DDD01712564	
Empirical formula	C18 H20 F N3 O2 S	
Formula weight	361.43	
Temperature	104(2) K	
Wavelength	1.54178 Å	
Crystal system	Hexagonal	
Space group	P 61	
Unit cell dimensions	a = 10.1357(1) Å	α= 90°.
	b = 10.1357(1) Å	β= 90°.
	c = 28.9939(6) Å	γ = 120°.
Volume	2579.55(7) $Å^3$	
Z	6	
Density (calculated)	1.396 Mg/m^3	
Absorption coefficient	1.913 mm^{-1}	
F(000)	1140	
Crystal size	0.075 x 0.075 x 0.015 mm^3	
Theta range for data collection	5.038 to 72.163°.	
Index ranges	-12<=h<=11, -12<=k<=12, -35<=l<=35	
Reflections collected	59548	
Independent reflections	3393 [R(int) = 0.0532]	
Completeness to theta = 70.000°	100.0 %	
Absorption correction	Semi-empirical from equivalents	
Max. and min. transmission	0.4990 and 0.3414	
Refinement method	Full-matrix least-squares on F^2	
Data / restraints / parameters	3393 / 1 / 235	
Goodness-of-fit on F^2	1.112	
Final R indices [I>2sigma(I)]	R1 = 0.0243, wR2 = 0.0569	
R indices (all data)	R1 = 0.0256, wR2 = 0.0577	
Absolute structure parameter	0.008(5)	
Extinction coefficient	n/a	
Largest diff. peak and hole	0.142 and -0.184 e.$Å^{-3}$	

Table S2. Summary of primers used in OSC gene replacement studies. Related to figure **6.**

**Primers**	**Sequence**
KO-*OSC*-F	5′-GCTCTACACAGAGCCATACGATGC-3′
KO-*PURO*-F	5′-GCTGCAAGAACTCTTCCTCACG-3′
KO-*HYG*-F	5′-CGTCTGTCGAGAAGTTTCTGATCG-3′
KO-3′ UTR-R	5′-AGGTAGAGTGTGACGTGAGAACG-3′
Probe-*OSC*-F	5′-CGTGAATGGGCTCACCACCAGC-3′
Probe-*OSC*-R	5′-CGAGTGCTTGTGCACATTGGC-3′
Probe-5′ UTR-F	5′-ATCTTGTTGCCCTTCACCAGC-3′
Probe-5′ UTR-R	5′-ACTTTCCTCGCGCGTCTTTCC-3′

Table S3. Collated EC_{50} data for WT, resistant and transgenic cell lines. Related to figures **2** and **3.**

Cell line	EC_{50} values, µM (fold change versus WT)							
	Compound **1**		(*S*)-**1**		(*R*)-**1**		BIBX-79	
Wild-type	0.5 ± 0.02	(-)	0.4 ± 0.01	(-)	3 ± 0.1	(-)	0.5 ± 0.01	(-)
RES I	26 ± 3	(51)	10 ± 2	(26)	> 50	(> 19)	24 ± 7	(47)
RES2	13 ± 0.8	(26)	6 ± 0.2	(16)	43 ± 2	(16)	7 ± 0.5	(13)
RES3	14 ± 2	(28)	6 ± 0.8	(16)	> 50	(> 19)	6 ± 0.5	(11)
RES4	10 ± 2	(19)	4 ± 0.7	(11)	> 50	(> 19)	8 ± 0.5	(15)
RES5	17 ± 1	(34)	8 ± 1	(21)	> 50	(> 19)	8 ± 2	(15)
OSC^{OE}	5 ± 0.1	(10)	4 ± 0.1	(10)	30 ± 2	(11)	5 ± 0.3	(10)
HP^{OE}	0.7 ± 0.03	(1.4)	0.5 ± 0.1	(1)	3 ± 0.5	(1.3)	0.4 ± 0.001	(0.8)

All EC_{50} values represent the weighted mean ± standard deviation of at least three biological replicates ($n \geq 3$) with each biological replicate comprised of two technical replicates.

Table S4. Summary of encoding SNPs identified in compound 1-resistant clones.

Related to figure **2.**

Protein	Gene ID	Encoding mutation	Resistant clone	Zygosity
Oxidosqualene cyclase	LdBPK.06.2.000670	C773F	V	Het
Hypothetical protein, conserved	LdBPK.08.2.000410	R1099G	IV	Het
Protein tyrosine kinase, putative	LdLV9.15.2.203710	Q607H	I	Het
HEAT repeats, putative	LdBPK.18.2.000670	P264S	III	Het
Protein of unknown function (DUF2946), putative	LdBPK.20.2.000420	R165M	V	Het
PRO8NT (NUC069), PrP8 N-terminal domain	LdBPK.35.2.004000	A2318V	III	Het

Table S5. Genes encoded by the 54.5 kb fragment of chromosome 6 enriched in compound 1-resistant parasites following selection of the cosmid library. 87% of all of all reads mapped to this genomic location. Genes common to all enriched fragments are highlighted in light blue. Related to figure **2**.

			LdBPKLV9 reads		LdBPK282A1 reads	
**LdBPKLV9 gene ID**	**LdBPK282A1 gene ID**	**Gene name**	**RPKM**	**Total**	**RPKM**	**Total**
LdBPK.06.2.000630	LdBPK_060630	Carbonic anhydrase family protein, putative	1342.3	6990	1421.2	7502
LdBPK.06.2.000640	LdBPK_060640	Putative phosphatase/protein of unknown function DUF89	6970.8	119545	7018.9	122016
LdBPK.06.2.000650	LdBPK_060650	Hypothetical protein, unknown function	16301.7	528431	16190.1	531992
LdBPK.06.2.000660	LdBPK_060660	Hypothetical protein, conserved	23923.4	1275852	23663.1	1282076
LdBPK.06.2.000670	LdBPK_060670	Oxidosqualene cyclase	25915.7	440919	25529.5	442484
LdBPK.06.2.000680	LdBPK_060680	Hypothetical protein, conserved	24303.2	280329	24054.8	281258
LdBPK.06.2.000690	LdBPK_060690	Hypothetical protein, conserved	17752.3	392670	17687.6	396589
LdBPK.06.2.000700	LdBPK_060700	Hypothetical protein, conserved	17700.8	73262	17693.5	74233
LdBPK.06.2.000710	LdBPK_060710	Ctf8, putative	13594.1	94082	13523.3	94872
LdBPK.06.2.000720	LdBPK_060720	Hypothetical protein, conserved	4552.9	203268	4577.5	207161

Table S6. List of top 10 proteins demonstrating thermal shift in the presence of compound (*S*)-1 in technical replicates. These proteins were identified as "hits" or potential targets by NPARC analysis. The criteria used to determine NPARC hits is described in STAR methods. Related to figure **4**.

Gene ID	Encoded protein	NPARC *p*-value	ΔT_m (1)	ΔT_m (2)
LdBPK_070460	hypothetical protein - conserved	1.2E-11	-9.71	-8.55
LdBPK_151590	hypothetical protein - conserved	1.6E-10	T >69	T >69
LdBPK_360230	SET domain protein - putative	4.6E-09	5.63	4.78
LdBPK_091220	hypothetical protein - conserved	1.7E-07	-13.39	C >69
LdBPK_060670	oxidosqualene cyclase	4.9E-07	3.83	2.93
LdBPK_210770	ATP-binding cassette protein subfamily E - member 1 - putative	7.6E-07	3.42	3.92
LdBPK_261880	Leucine Rich repeat - putative	7.6E-07	3.98	5.13
LdBPK_190690	kinesin - putative	4.1E-06	T >69	T >69
LdBPK_200750	hypothetical protein - conserved	4.9E-06	6.34	7.65
LdBPK_090160	FHA domain/Ring finger domain/Zinc finger - C3HC4 type (RING finger) containing protein - putative	9.3E-06	9.43	2.67

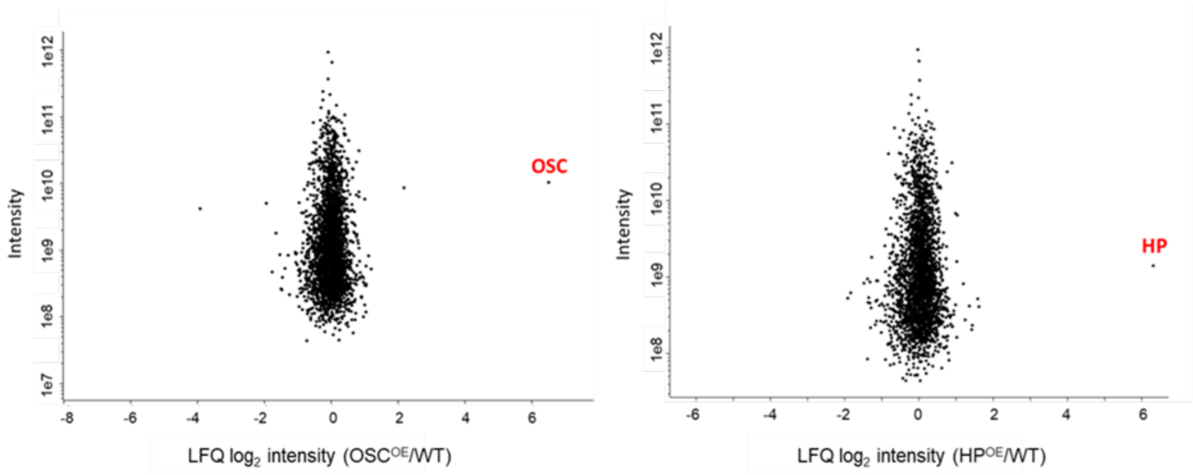

Figure S1. Label-free proteomics quantitation of OSC and HP transgenic cell lines. Relative levels of proteins in WT and transgenic cell lines were compared. Details of this analysis can be found in the materials and methods. Related to figure **3**.

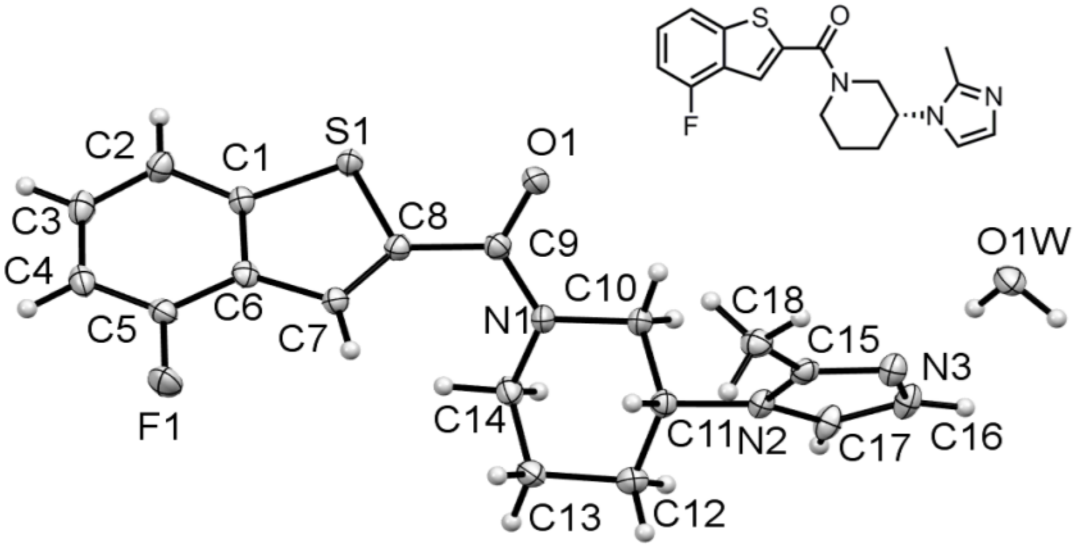

Figure S2. X-ray structure of compound 1 (*R*). The Oak Ridge Thermal Ellipsoid Plot (ORTEP) structure of (*R*)-(4-fluorobenzo[*b*]thiophen-2-yl)(3-(2-methyl-1*H*-imidazol-1-yl)piperidin-1-yl)methanone (compound **1** (*R*)). Please note, the crystal structure shows the presence of a water molecule indicating that the compound is a mono hydrate. Related to Figure **1**, table **1** and table **S1**.

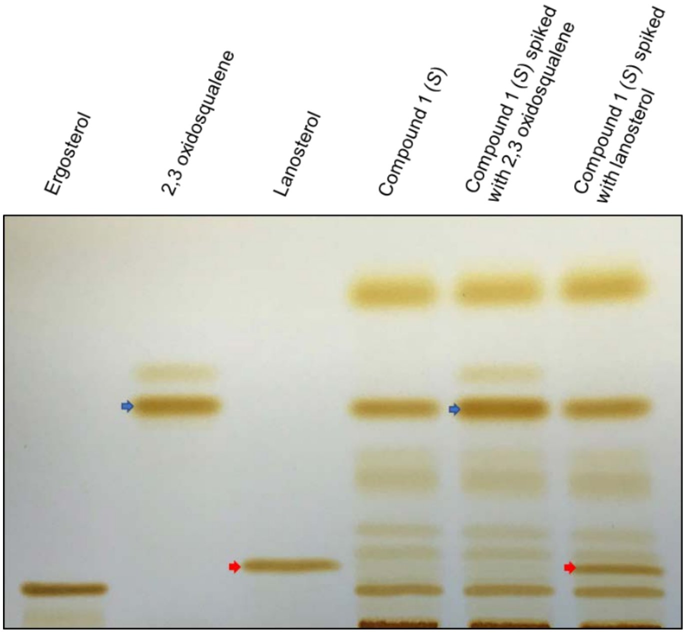

Figure S3. Effect of compound (*S*)-1 on the sterol biosynthesis in *L. donovani*. Sterols extracted from WT promastigotes treated with compound (*S*)-**1** (1.5 µM, 96 h) and identical samples spiked with 2,3-oxidosqualene (8 µg) were separated on HPTLC silica gel in heptane: ethyl ether: acetic acid buffer. Ergosterol, 2,3-oxidosqualene (blue arrows) and lanosterol (red arrows) standards (1 mg mL^{-1}) were run in parallel and the unsaturated double bonds of separated lipids were stained with iodine vapour. Related to figure **4**.

A

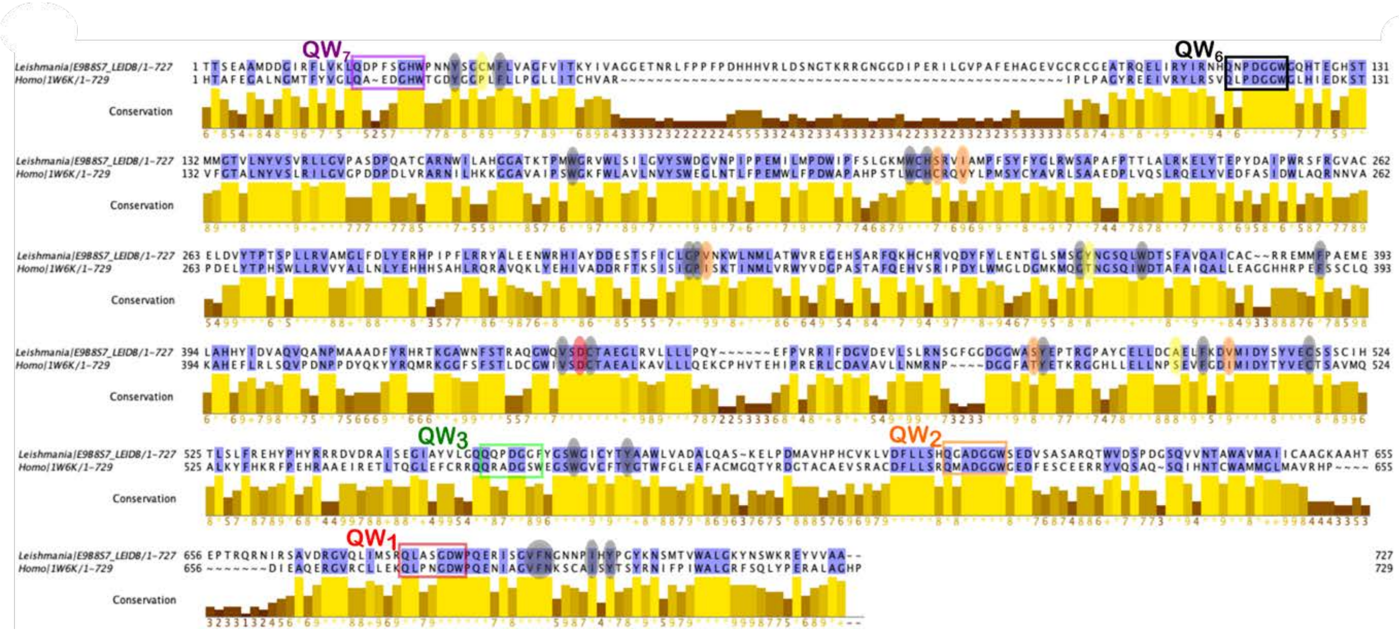

B

Human	Ld
Y98	Y318
P101	C321
F103	F323
W192	W462
W230	W500
H232	H502
C233	S503
V236	I506
G336	G606
P337	P607
I338	V608
G380	G650
T381	Y651
W387	W657
F444	F712
V453	V721
D455	D723
C456	C724
T502	S768
Y503	Y769
S518	A784
F521	F787
I524	V790
C533	C799
W581	W847
Y587	Y853
V695	V972
F696	F973
N697	N974
I702	I979
Y704	Y981

Figure S4. Edited alignment of the query sequence of *Ld*OSC and the template sequence of *h*OSC. (A) The alignment used to build the homology model of *Ld*OSC and the level of sequence conservation between the aligned residues. QW1-3 and QW6-7 motifs are highlighted red, orange green, black and purple, respectively. Identical residues are highlighted in blue. Residues that form the lanosterol binding site are highlighted with grey circles (identical), yellow circles (no match) and orange circles (residues with similar properties). The catalytic residue (Asp) is circled in red. In panel B, the residues of the binding site of *h*OSC and *Ld*OSC are listed. Related to figure **5**.

A

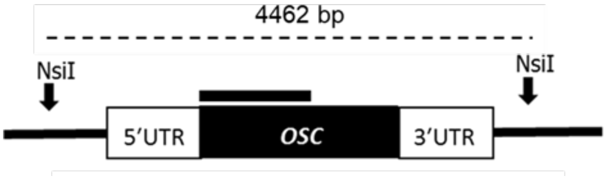

B

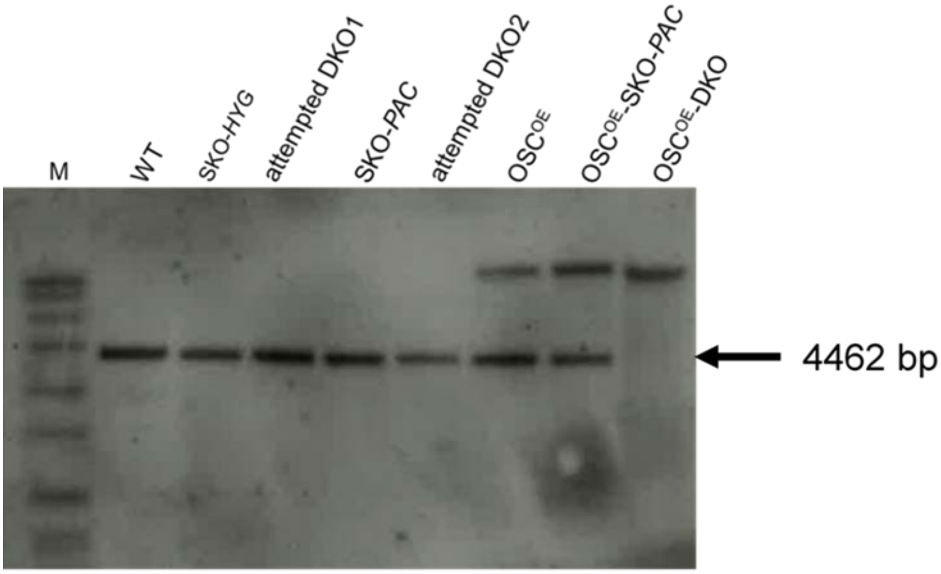

Figure S5. Assessing the essentiality of OSC in *L. donovani* promastigotes. (A) Schematic representation of the *OSC* locus. Black bars represent the region of the open reading frame of *OSC* that was DIG-labelled and used as a probe in Southern blot analysis. *Nsi*I sites with expected fragment sizes are shown. (B) Southern blot analysis of *Nsi*I-digested genomic DNA (~5 µg) from wild-type *L. donovani* (*Ld*BOB) (WT), *OSC*-single knockout clones (SKO-*HYG* and *PAC*), attempted double knockout cells (attempted DKO 1 and 2), OSC overexpressing cells (*OSC*^{OE}), and double knockout cells overexpressing OSC (*OSC*^{OE}-DKO). A DIG-labelled fragment of *OSC* was used as a probe. Related to figure **6**.

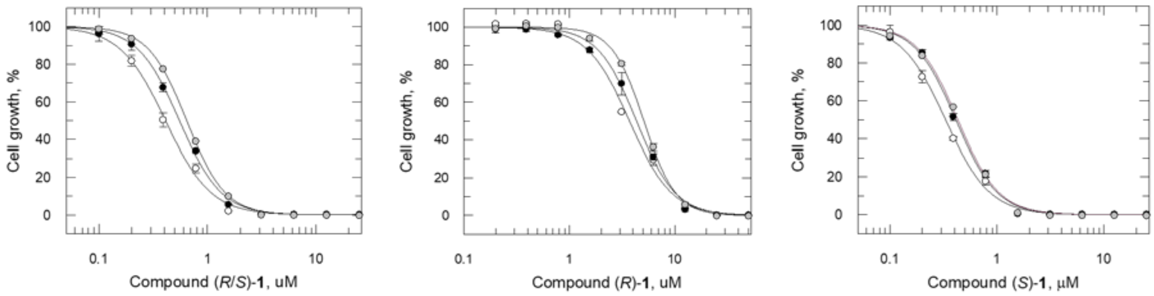

Figure S6. The effect of varying levels of FCS on the potency of compound 1 and enantiomers. Dose response curves with *L. donovani* promastigotes grown in culture media supplemented with 5, 10 or 20% FCS, respectively. For compound (*R*/*S*)-**1,** EC_{50} values of 0.4 ± 0.01, 0.55 ± 0.01 and 0.6 ± 0.008 µM were determined for parasites grown in 5, 10 and 20% FCS, respectively. For (*R*)-1, values of 3.7 ± 0.01, 4.3 ± 0.1 and 5 ± 0.08 µM were determined. Finally, for compound (*S*)-**1**, values of 0.3 ± 0.01, 0.41 ± 0.01 and 0.43 ± 0.01 µM were determined. All data represent mean ± SD of duplicate technical replicates. Related to table **1**.

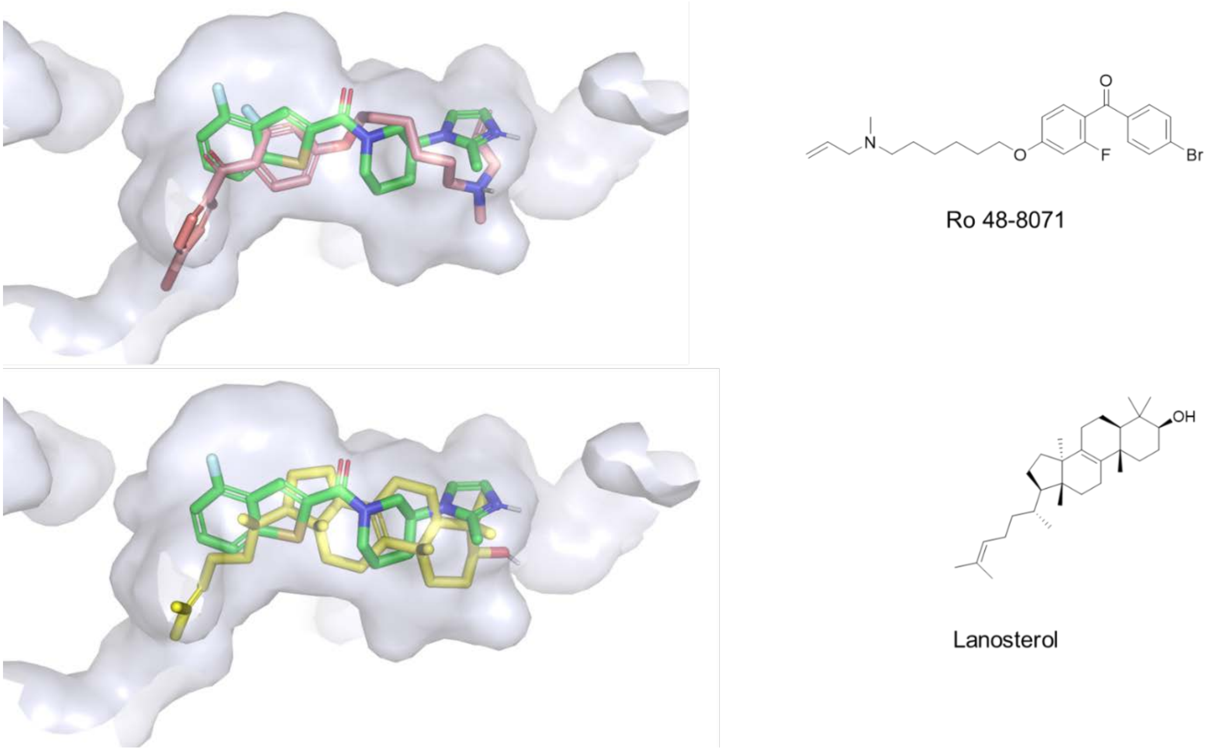

Figure S7. **Comparison of substrate and inhibitor binding in the *Ld*OSC active site.** Compound (*S*)-**1** binding in the *Ld*OSC homology model (best scoring pose) is shown in green. Binding of inhibitor *h*OSC inhibitor Ro 48-8071 (pink) and the substrate lanosterol (yellow) are superimposed. The molecular surface of the *Ld*OSC active site is shown in grey. Related to figure **5**.
