# Supplementary material for: Multiple unbiased approaches identify oxidosqualene cyclase as the molecular target of a promising anti-leishmanial
Source: Cell Chem Biol. 2021 May 20;28(5):711–721.e8. doi: 10.1016/j.chembiol.2021.02.008 (PMC8153249; doi:10.1016/j.chembiol.2021.02.008)
Supplement: Document S2. Article plus Supplemental information [file mmc2.pdf]

Article

Cell Chemical Biology

Multiple unbiased approaches identify oxidosqualene cyclase as the molecular target of a promising anti-leishmanial

Graphical abstract

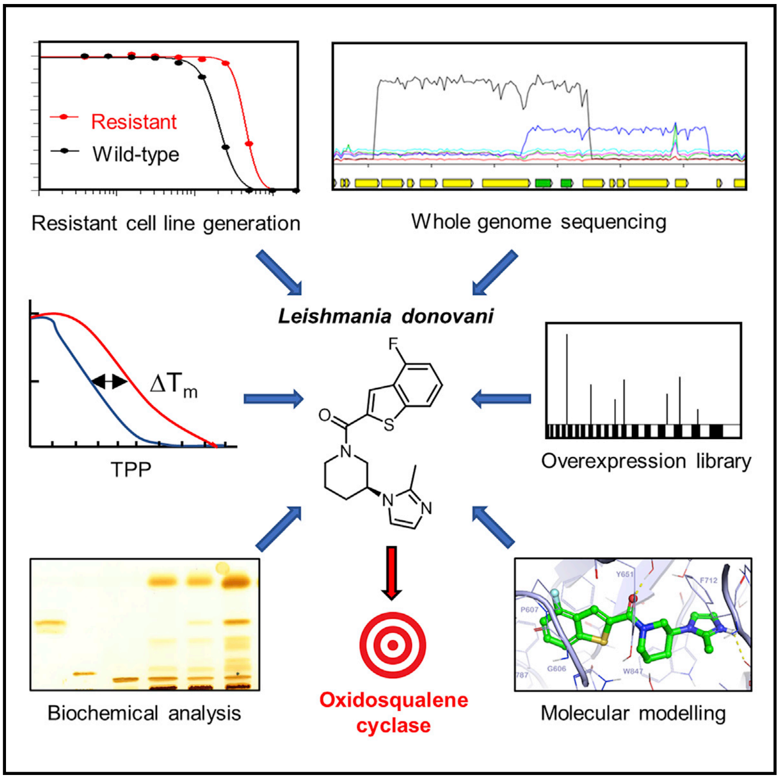

Highlights

- Genetics and chemo-proteomics identify the target of a promising anti-leishmanial
- Biochemical assays confirm the direct inhibition of oxidosqualene cyclase in cells
- Docking and modeling studies identify key interactions between compound and target
- Strategies to improve the potency of this benzothiophene are proposed

Authors

Luciana S. Paradela, Richard J. Wall, Sandra Carvalho, ..., Julio Martin, Ian H. Gilbert, Susan Wyllie

Correspondence

s.wyllie@dundee.ac.uk

In brief

Paradela et al. investigated the mechanism of action of a promising anti-leishmanial. Using genetics, chemo-proteomics, and biochemical approaches, the target of this benzothiophene was confirmed as oxidosqualene cyclase, a key enzyme of sterol biosynthesis. Docking and modeling identified key interactions between this compound and the active site of this enzyme.

Paradela et al., 2021, Cell Chemical Biology *28*, 711–721
May 20, 2021 © 2021 The Author(s). Published by Elsevier Ltd.
https://doi.org/10.1016/j.chembiol.2021.02.008

Article

Multiple unbiased approaches identify oxidosqualene cyclase as the molecular target of a promising anti-leishmanial

Luciana S. Paradela,[1] Richard J. Wall,[1] Sandra Carvalho,[1] Giulia Chemi,[2] Victoriano Corpas-Lopez,[1] Eoin Moynihan,[1] Davide Bello,[1] Stephen Patterson,[1] Maria Lucia S. Güther,[1] Alan H. Fairlamb,[1] Michael A.J. Ferguson,[1] Fabio Zuccotto,[2] Julio Martin,[3] Ian H. Gilbert,[2] and Susan Wyllie[1,4,*]

[1]Division of Biological Chemistry and Drug Discovery, Wellcome Centre for Anti-Infectives Research, School of Life Sciences, University of Dundee, Dow Street, Dundee DD1 5EH, UK

[2]Drug Discovery Unit, Wellcome Centre for Anti-Infectives Research, School of Life Sciences, University of Dundee, Dow Street, Dundee DD1 5EH, UK

[3]Global Health R&D, GlaxoSmithKline, Tres Cantos 28760, Spain

[4]Lead contact

*Correspondence: s.wyllie@dundee.ac.uk

https://doi.org/10.1016/j.chembiol.2021.02.008

SUMMARY

Phenotypic screening identified a benzothiophene compound with activity against *Leishmania donovani*, the causative agent of visceral leishmaniasis. Using multiple orthogonal approaches, oxidosqualene cyclase (OSC), a key enzyme of sterol biosynthesis, was identified as the target of this racemic compound and its enantiomers. Whole genome sequencing and screening of a genome-wide overexpression library confirmed that *OSC* gene amplification is associated with resistance to compound 1. Introduction of an ectopic copy of the *OSC* gene into wild-type cells reduced susceptibility to these compounds confirming the role of this enzyme in resistance. Biochemical analyses demonstrated the accumulation of the substrate of OSC and depletion of its product in compound (*S*)-1-treated-promastigotes and cell-free membrane preparations, respectively. Thermal proteome profiling confirmed that compound (*S*)-1 binds directly to OSC. Finally, modeling and docking studies identified key interactions between compound (*S*)-1 and the *Ld*OSC active site. Strategies to improve the potency for this promising anti-leishmanial are proposed.

INTRODUCTION

The protozoan parasites *Trypanosoma cruzi*, *Trypanosoma brucei,* and *Leishmania* spp. are the causative agents of the vector-borne diseases African sleeping sickness, Chagas disease, and the leishmaniases. Collectively, these diseases are responsible for more than 50,000 fatalities annually and the loss of more than 4,600,000 disease-adjusted life years (www.who.int/leishmaniasis/burden). Trypanosomatid diseases are principally diseases of poverty and inflict an enormous economic burden on some of the poorest countries on earth. Over the past 5 years, treatment options for African sleeping sickness have improved immeasurably with the introduction of nifurtimox-eflornithine combination therapy (NECT) to replace the more toxic organo-arsenical, melarsoprol (Priotto et al., 2009) and the licensing of the oral drug fexinidazole as an alternative to more complex parenteral regimens such as NECT (Pelfrene et al., 2019). Improved therapeutics, alongside robust surveillance screening programs and vector control measures, has significantly reduced cases of African sleeping sickness, raising hopes that this disease may be eliminated as a public health problem in the near future (Barrett, 2018). Unfortunately, new drugs for Chagas disease and leishmaniasis have been more difficult to develop. Current drugs suffer from a range of serious problems, including severe toxicity (Aldasoro et al., 2018; Soto and Soto, 2006), emerging drug resistance (Croft et al., 2006; Mueller et al., 2007), and poor efficacy (Jackson et al., 2010; Yun et al., 2009). New drugs that are safer, efficacious, and suitable for use in resource-poor settings are urgently required for the treatment of these neglected tropical diseases.

The principal goals of anti-trypanosomal drug discovery programs are to develop novel therapeutics that demonstrate improved efficacy with minimal host toxicity, are suitable for single-dose oral administration, and have the potential for use in a future combination therapy. However, progress has been hindered by the lack of robustly validated drug targets in *T. cruzi* and *Leishmania donovani* or *Leishmania infantum*, causative agents for Chagas disease and visceral leishmaniasis, respectively. As a consequence, almost all compounds currently in drug development pipelines for both diseases evolved from primary hits identified through whole-cell (phenotypic) screening of compounds (www.dndi.org) (Don and Ioset, 2014). Phenotypic approaches, although effective,

This is an open access article under the CC BY license (http://creativecommons.org/licenses/by/4.0/).

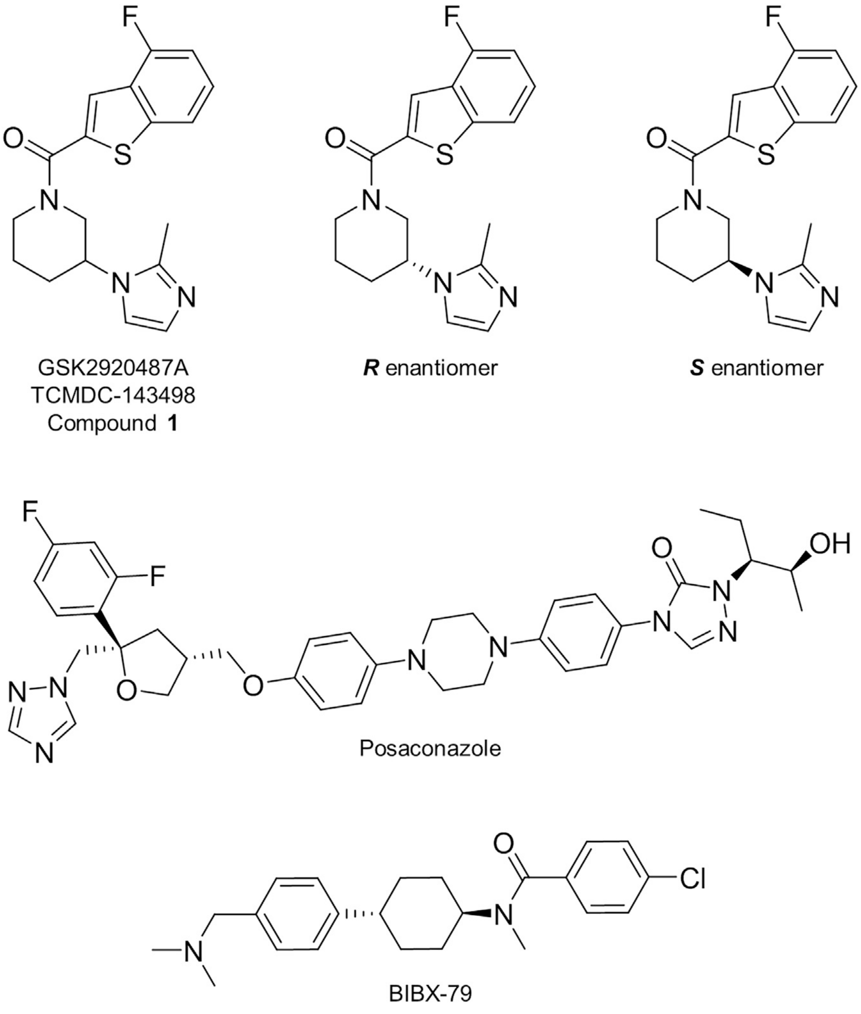

Figure 1. Chemical structures

do not provide information regarding the mechanism(s) of action (MoA) or specific molecular target(s) of active compounds. This information can be invaluable in the development of these compounds into selective and potent anti-parasitic agents, by addressing issues such as poor pharmacokinetic properties and host toxicity. MoA studies can also play a pivotal role in the management of drug discovery portfolios. For instance, compounds that act via mechanisms previously confirmed as unsuitable for drug development can be efficiently de-prioritized (Riley et al., 2015; Wall et al., 2018). Compound series found to inhibit the same molecular target(s) can be prioritized and rationalized, thus mitigating against the overpopulation of pipelines with compounds acting via the same mechanism (Wall et al., 2020). Furthermore, knowledge of the target pathway can be of potential value in optimizing drug combination therapy.

Here, we use orthogonal genetic, molecular, and biochemical approaches to determine the MoA of a benzothiophene compound demonstrating promising anti-leishmanial activity. Our comprehensive studies reveal that this compound specifically targets oxidosqualene cyclase (OSC), a key enzyme in the sterol biosynthetic pathway of these parasites. The implications of developing compounds with this MoA as anti-leishmanials of the future is discussed.

RESULTS

Compound selection

GSK's Kinetoboxes are three open-access compound collections assembled following high-throughput phenotypic screening of 1.8M compounds against *T. brucei*, *L. donovani* and *T. cruzi* (Peña et al., 2015). TCMDC-143498 (compound **1,** Figure 1), also known as GSK2920487A, is included in the *T. cruzi* box, having demonstrated promising activity against the intracellular stage of the parasite (half maximal effective concentration [EC_{50}] value of 0.8 μM). Interestingly, this compound was also moderately active against *L. donovani* axenic amastigotes and promastigotes (EC_{50} values of 20 μM and 0.5 μM, respectively) while demonstrating limited activity against mammalian cell lines (HepG2) (Table 1). The principal aim of our current study was to gain an understanding of the mechanism of action and/or molecular target(s) of compound **1** to facilitate the development of more potent and selective versions of this compound. Our studies focused on using multiple unbiased approaches to determine the MoA of this compound in *L. donovani*.

Resistance generation followed by whole genome sequencing

As a first step toward identifying the molecular target(s) of compound **1**, *L. donovani* promastigote cell lines resistant to this benzothiophene were selected. Starting at 0.5 μM (1× EC_{50}), five independent clonal lines of compound-susceptible parasites were exposed to stepwise increasing levels of compound **1** for a total of 120 days, until they were routinely growing at concentrations equivalent to >20× the established EC_{50} value (12 μM) (Figure 2A). The five independently generated resistant cell lines were cloned by limiting dilution and clones were assessed for susceptibility to compound **1**. The resulting clones were between 18- and 51-fold less sensitive to compound **1** than the wild-type (WT) parental clone (Figure 2B and Table S3). In each case, the resistance demonstrated by each clone was stable over at least 10 passages in culture in the absence of compound.

Genomic DNA recovered from the five resistant clones was analyzed by whole genome sequencing (WGS). Notably, copy

CellPress
OPEN ACCESS

Table 1. Collated EC_{50} values for compound 1 and related analogues

	EC_{50} values, μM			
Compound	Promastigotes	Axenic amastigotes	Intramacrophage	HepG2
Compound **1**	0.5 ± 0.02	20 ± 4	39 ± 8	>50
(*R*)-**1**	2.6 ± 0.1	>50	>50	>50
(*S*)-**1**	0.4 ± 0.01	17 ± 3	31 ± 7	>50
BIBX-79	0.5 ± 0.01	>50	ND	5 ± 0.4

EC_{50} values represent the weighted mean ± standard deviation of at least two biological replicates ($n \geq 2$) with each biological replicate comprised of three technical replicates. The exception is intramacrophage amastigotes EC_{50} values, where data represent the mean ± standard deviation of two technical replicates and representative of two biological replicates. ND, not determined.

number variant analysis revealed that all five resistant clones maintained amplified fragments of, or indeed entire copies of, chromosome 6. Resistant lines RES I, RES III, and RES IV had two additional copies of chromosome 6 compared with WT, while RES V maintained three additional copies (Figure 2C). RES I also maintained an additional 13 copies of a 30.9 kb region of chromosome 6. Furthermore, RES II resistant parasites had amplified a 44.3-kb fragment of chromosome 6, maintaining more than 34 extra copies of this chromosomal fragment compared with WT, drug-sensitive cells. Rationalizing the regions of chromosome 6 amplified in resistant clones indicates that a fragment encoding two complete gene-coding sequences is common to all. This coincident chromosomal fragment encodes oxidosqualene cyclase (*OSC*, LdBPK.06.2.000670) and a hypothetical protein (*HP*, LdBPK.06.2.000680).

A total of six single nucleotide polymorphisms (SNPs) encoding amino acid changes, relative to our parental clone, were identified in the genomes of compound **1**-resistant parasites (Table S4); however, no SNP was common to all cell lines. Notably, in RES V, a single heterozygous SNP was identified in the gene encoding OSC. This SNP encodes a Cys to Phe mutation at position 778 in this enzyme, a key component of the sterol biosynthetic pathway of *L. donovani*.

Screening of compound 1 against an *L. donovani* genome-wide overexpression library

As a parallel unbiased approach to investigate MoA, compound **1** was also screened against our genome-wide cosmid-based overexpression library (Corpas-Lopez et al., 2019). The principle behind this gain-of-function approach is that overexpression of a drug target can result in resistance to the corresponding drug by increasing the pool of functional protein or by reducing free drug through binding. *L. donovani* promastigotes were transfected with a pooled population of cosmids containing genomic DNA fragments of between 35 and 45 kb. The final transfected library provides a >15-fold genome coverage with 99% of genes represented. The library was selected with 1 μM (2× EC_{50}) compound **1** for 5 days and for a further 4 days at 2 μM. Following compound selection, cosmids maintained by the "resistant" parasite population were harvested and analyzed by next-generation sequencing. Mapping of overexpressed inserts to an assembled *L. donovani* genome revealed that 87% of all mapped reads aligned to a 54.5-kb region on chromosome 6 (Figures 2D and 2E). This region is composed of 10 genes (Table S5); however, only two genes were flanked by all the opposing barcodes: LdBPK.06.2.000670 encoding OSC and LdBPK.06.2.000680 encoding HP. Collectively, these data support our WGS analysis and the hypothesis that one of these proteins may be the molecular target of compound **1** or a key resistance determinant.

Target validation

To interrogate the potential role(s) of each protein in the MoA of compound **1**, cell lines overexpressing both putative targets were generated. Elevated levels of HP and OSC in these transgenic promastigotes, compared with WT, were confirmed by label-free MS quantification (Figure S1). While overexpression of HP had little or no effect on the potency of compound **1** (Figure 3A), cells overexpressing OSC were ~10-fold less sensitive to compound **1** in comparison to WT. These data identify OSC overexpression as the driver of compound **1**-resistance and identify this enzyme as the likely target of this benzothiophene. Next, we investigated the role of the OSC (Cys778Phe) mutation identified in RES V in resistance (Figure 3B). Promastigotes overexpressing the mutated version of this cyclase demonstrated the same level of susceptibility to compound **1** as WT. The fact that cells bearing elevated levels of this mutated enzyme remain susceptible to compound **1** not only rules out this mutation as playing any role in resistance, but also suggests that the mutated version of this enzyme may be nonfunctional.

OSC, also known as lanosterol synthase or ERG7, is a key enzyme in sterol biosynthesis, catalyzing the cyclization of 2,3-oxidosqualene to lanosterol (Figure 4A). A number of specific inhibitors of OSC have been described in the literature (Mark et al., 1996; Morand et al., 1997; Phillips et al., 2019) with a variety of different applications such as anti-fungal (Rose et al., 1996), anti-microbial (Hinshaw et al., 2003), and anti-cancer agents (Liang et al., 2014, 2016; Maione et al., 2015). Most notably, Buckner and colleagues (2001) have demonstrated that inhibitors of OSC can be potent inhibitors of *T. cruzi* growth *in vitro*. Consequently, we assessed the potency of an established OSC inhibitor, BIBX-79 (Mark et al., 1996), to evaluate potential cross-resistance against our transgenic and resistant cell lines. Indeed, OSC-overexpressing promastigotes were 10-fold less susceptible to BIBX-79 than WT (Figure 3C), demonstrating an equivalent level of resistance to that seen with compound **1**. Similarly, all compound **1**-resistant clones (RES I-V) were cross-resistant to BIBX-79 (Table S3). The cross-resistance profiles shared by compound **1** and BIBX-79 are entirely consistent with a shared mechanism of action and support OSC as the molecular target of both compounds in *L. donovani*.

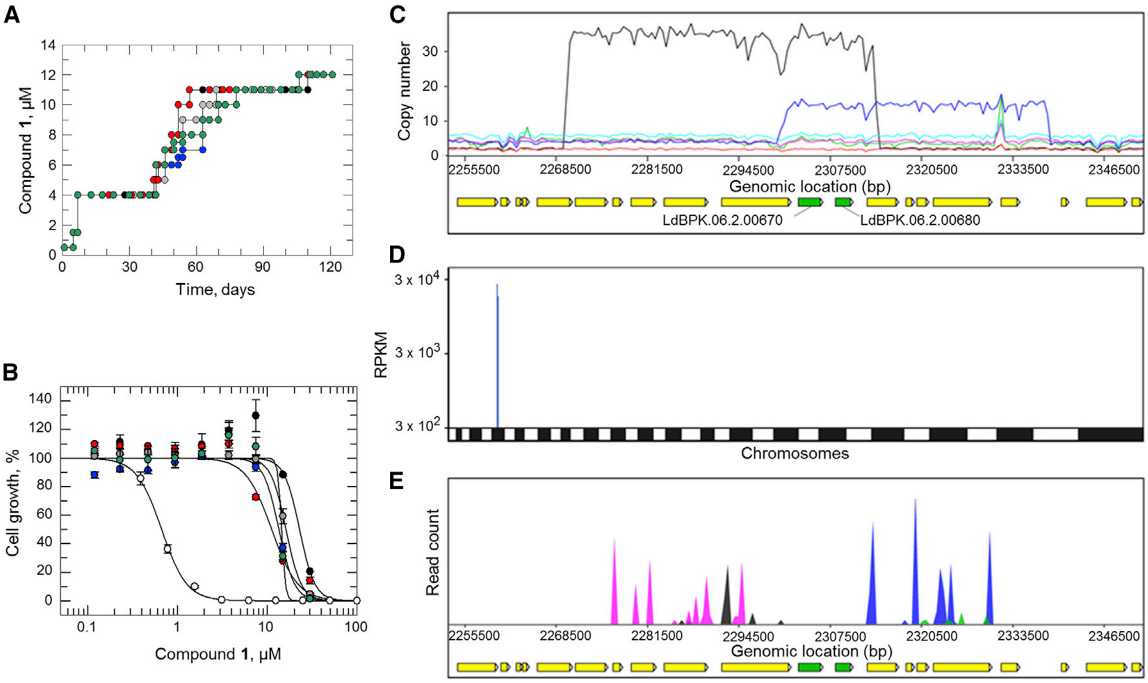

Figure 2. Target deconvolution studies with compound 1

(A) Schematic representation of the generation of compound **1**-resistant cell lines in *L. donovani*. Each passage of cells in culture (circles, lines 1-V) is indicated with cell lines I-V indicated in black, green, gray, blue, and red, respectively.

(B) EC_{50} values for compound **1** were determined for WT (white circles) and cloned resistant cell lines I–V (black, gray, blue, red, and green circles, respectively). These curves are the nonlinear fits of data using a two-parameter EC_{50} equation provided by GraFit. An EC_{50} value of 0.7 ± 0.01 μM was determined for compound **1** against WT promastigotes. EC_{50} values for resistant clones I–V were 23 ± 4, 16 ± 0.3, 13 ± 1, 11 ± 1, and 14 ± 6 μM, respectively. These EC_{50} curves and values are from one biological replicate, composed of two technical replicates. Collated datasets reporting the weighted mean ± SD of multiple biological replicates are summarized in Table S3.

(C) Copy number variations in resistant clones relative to WT. Amplification of chromosome 6 (or fragments) are evident in all resistant clones. Resistant clones are indicated as follows: RES I (blue), RES II (black), RES III (green), RES IV (pink), and RES V (cyan); WT clone is shown in red. *OSC* (LdBPK.06.2.000670) and *HP* (LdBPK._006.2.000680) green bars; other genes, yellow bars.

(D) Genome-wide map indicating cosmid library hits from screening of compound **1**. A single primary hit was identified, indicated in blue.

(E) Focus on primary "hit" on chromosome 6. *OSC* and *HP* genes shown as green bars; other genes, as yellow bars. The blue/pink and black/green peaks indicate independent cosmid inserts in different orientations.

Stereochemistry of compound 1

Compound **1** has a chiral center at the 3-position of the piperidine ring, thus previously reported screening data associated with this benzothiozine has been obtained with a racemate (1:1 mixture of *R* and *S* enantiomers) (Peña et al., 2015). In order to profile the biological activity of the individual enantiomers, semi-preparative chiral chromatography was used to separate both enantiomers of compound **1**. The absolute stereochemistry of the enantiomer eluted first following chromatographic separation (retention time 11.9 min) was established by X-ray crystallography to be *R* (Figure S2 and Table S1). Therefore, the enantiomer eluted in peak 2 (retention time 15.5 min) is compound (*S*)-**1**. The enantiomeric excess values of the separated samples of (*R*) and (*S*) were measured by chiral HPLC and found to be >99.7% and 98.6%, respectively, indicating that both samples were of excellent optical purity. The potency of each enantiomer was then determined against the various developmental stages of *L. donovani* and also against the transgenic cell lines developed in the course of our studies (Tables 1 and S3). While both enantiomers appear to specifically target OSC, as evidenced by reduced sensitivity in cell lines overexpressing this enzyme, in all assays, the *S* enantiomer was found to be at least 10-fold more potent than (*R*)-**1**. Of particular note, (*S*)-**1** demonstrated activity against intramacrophage amastigotes (31 ± 7 μM, Table 1), while (*R*)-**1** was inactive at all concentrations tested.

Evidence of target engagement

Our cumulative data provide compelling circumstantial evidence that OSC is the molecular target of compound **1** in *L. donovani*. Here, we sought to provide biochemical evidence of the inhibition of this enzyme by compound **1** and to investigate the broader effects on sterol biosynthesis. Thin-layer chromatography (TLC) was used to separate and then identify specific sterols in compound-treated and untreated promastigotes. Cells were treated with compound **1** (racemate), compound (*S*)-**1**, BIBX-79, or posaconazole for 96 h. Posaconazole targets sterol 14α-demethylase (CYP51), the enzyme immediately downstream of OSC that uses lanosterol as a substrate to produce 4,4-dimethylcholesta-8,14,24-trien-3β-ol. Neutral lipids were then extracted and separated by TLC alongside 2,3-oxidosqualene, lanosterol, and ergosterol standards (Figures 4B and S3). In keeping with our assertion that compound **1** and its enantiomers

CellPress
OPEN ACCESS

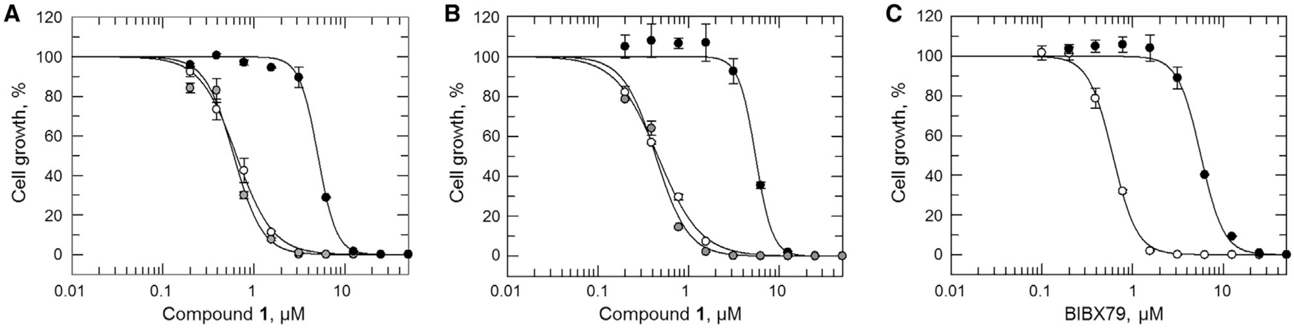

Figure 3. Validation of OSC as the molecular target of compound 1

(A) Dose–response curves for WT (white), OSC-overexpressing (black), and HP-overexpressing (gray) clones treated with compound **1**. EC_{50} values of 0.6 ± 0.01, 5 ± 0.1, and 0.6 ± 0.03 μM were determined for WT, OSC-overexpressing, and HP-overexpressing promastigotes, respectively.

(B) EC_{50} values for WT (white), OSC-overexpressing (black), and OSC^{C778F}-overexpressing (gray) promastigotes treated with compound **1** were 0.5 ± 0.01, 6 ± 0.3, and 0.4 ± 0.02 μM, respectively.

(C) EC_{50} values for WT (white) and OSC-overexpressing cells (black) treated with BIBX-79 were 0.6 ± 0.01 and 6 ± 0.2, respectively. All EC_{50} curves and values are from one biological replicate, composed of two technical replicates. Collated datasets reporting the weighted mean ± SD of multiple biological replicates are summarized in Table S3.

inhibit OSC, promastigotes treated with (*S*)-**1** were found to accumulate significant amounts of the substrate of this enzyme, 2,3-oxidosqualene, in comparison with untreated cells. Similarly, promastigotes treated with BIBX-79 were also found to accumulate this substrate. Levels of lanosterol, the product of OSC, would be reasonably expected to decrease in the presence of an OSC-specific inhibitor; however, lanosterol was below the limits of detection in the majority of our TLC studies, even in untreated parasites. This is likely due to the rapid turnover and generally low basal levels of lanosterol, a substrate for the zymosterol biosynthetic pathway (Figure 4A). Indeed, lanosterol is only visible, at very modest levels, in parasites treated with the CYP51-specific inhibitor posaconazole. In *T. cruzi*, CYP51 has been identified as a promiscuous drug target, with up to 80% of screening hits found to inhibit this enzyme (Riley et al., 2015). Previous studies report that compound **1** does not inhibit CYP51 in *in vitro* assays (Peña et al., 2015). The fact that the sterol signature of our posaconazole-treated promastigotes is distinct from that seen with compound **1**-treated parasites supports this observation.

Crude, cell-free membrane preparations were obtained by subjecting *L. donovani* promastigotes to nitrogen cavitation. Our aim was to use this cell-free, membrane system to focus solely on the reaction catalyzed by OSC, an established membrane protein. 2,3-oxidosqualene (OSC substrate) was added to these washed membranes prior to incubation at 37°C for 24 h. Sterols were then extracted, separated, and identified by TLC (Figure 4C). The production of lanosterol (OSC product) was clearly evident in assays supplemented with exogenous 2,3-oxidosqualene, confirming that OSC was active in these *L. donovani* membrane preparations. Lanosterol was undetectable in assays not supplemented with substrate. Most importantly, the production of lanosterol by membrane preparations supplemented with 2,3-oxidosqualene was ablated by the presence of the established OSC-inhibitor BIBX-79 and compound (*S*)-**1** at a range of concentrations. Collectively, these data provide direct, biochemical evidence that compound (*S*)-**1** inhibits the ergosterol biosynthetic pathway specifically at the 2,3-oxidosqualene to lanosterol step catalyzed by OSC.

Finally, thermal proteome profiling (TPP) was used as an approach to confirm the direct binding of compound (*S*)-**1** to OSC. TPP is based on the principle that binding of a drug to its protein target can significantly alter the thermal stability of that protein (Jafari et al., 2014). Briefly, *L. donovani* promastigotes were treated with compound (*S*)-**1** (10× established EC_{50} value) or DMSO vehicle. Lysates of treated promastigotes were then prepared and maintained in the continuous presence of compound or vehicle. Aliquots of each lysate were then incubated at designated temperatures (33–69°C), and for each temperature, insoluble (denatured) proteins were removed. The resulting soluble protein samples were reduced, alkylated, and digested with trypsin prior to derivatization with tandem mass tags. Pooled peptides were fractionated by HPLC and analyzed by LC/MS-MS prior to identification and quantitation. The melting points of each identified protein were then established. Full melt curves were established for 5,083 proteins, representing 68.3% coverage of the *L. donovani* proteome. The top 10 proteins demonstrating thermal shift in the presence of compound (*S*)-**1** and confirmed as legitimate "hits" by nonparametric analysis of response curves (NPARC), are summarized in Table S6. *Ld*OSC ranks as the fifth strongest "hit" in this unbiased, proteome-wide analysis, with individual melt curves revealing that the thermal stability of this enzyme increased by an average of 3.4°C in the presence of compound (*S*)-**1** (Figure 4D). These data confirm that compound (*S*)-**1** interacts directly with OSC and, in combination with our other studies, identifies *Ld*OSC as the molecular target of this benzothiophene compound.

Molecular modeling

With the aim of understanding the molecular basis for the superior potency of compound (*S*)-**1** and identifying key interactions within the active site of *Ld*OSC, a homology model was generated using the structure of the human OSC orthologue complexed with lanosterol as a template (Thoma et al., 2004). The consensus *Ld-human* OSC sequence used to build the model

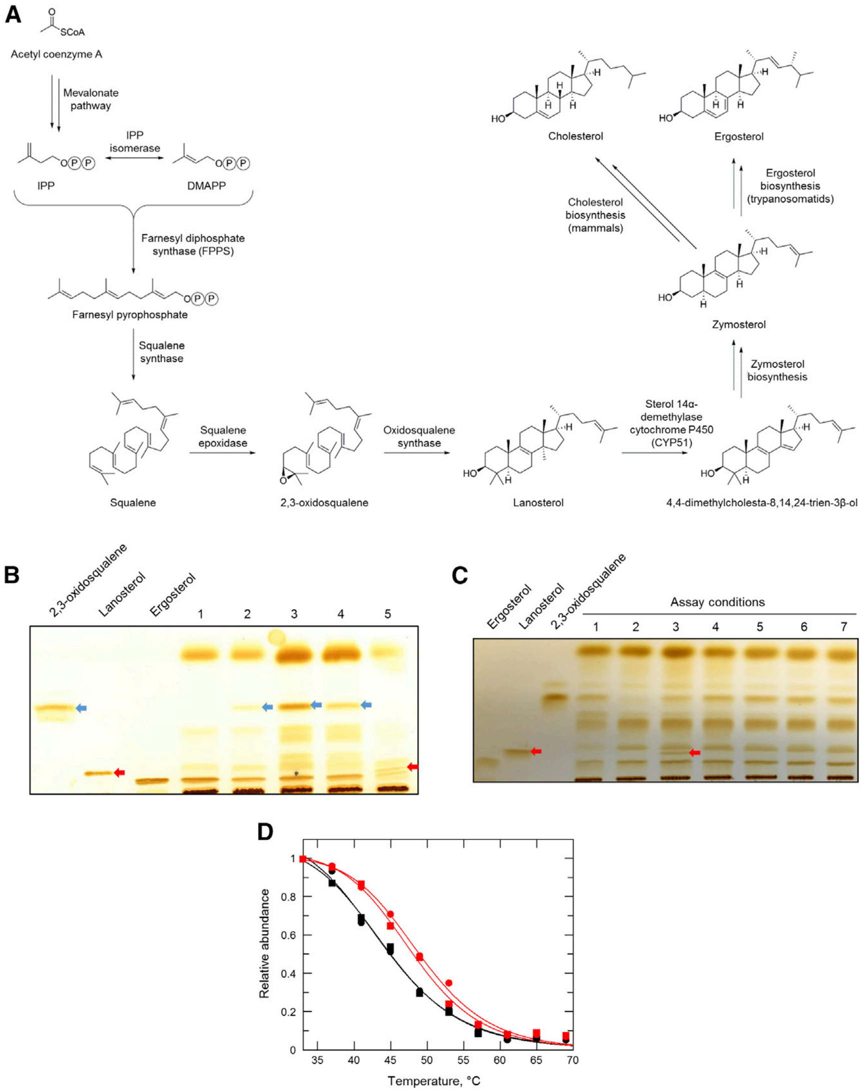

Figure 4. Effect of compound 1 on the sterol biosynthesis in *L. donovani*

(A) Sterol biosynthetic pathway of *L. donovani*.

(B) Sterols extracted from WT promastigotes (lane 1); promastigotes treated with compound **1** (racemate) (lane 2), compound (*S*)-**1** (lane 3), BIBX-79 (lane 4), and posaconazole (lane 5) for 96 h and analyzed by normal phase silica TLC. Ergosterol, lanosterol, and 2,3-oxidosqualene standards (1 mg·mL^{-1}) were run in parallel and the unsaturated double bonds of separated lipids were stained with iodine vapor. Bands representing 2,3-oxidosqualene and lanosterol are indicated by blue and red arrows, respectively.

(C) The activity of *L. donovani* OSC was monitored in cell-free membrane preparations by TLC. Crude membrane preparations were treated as follows: 2,3-oxidosqualene (OSC substrate) added and sterols immediately extracted (lane 1); no additions and sterols immediately extracted (lane 2); 2,3-oxidosqualene added, incubated at 37°C for 24 h and sterols extracted (lane 3); 2,3-oxidosqualene and compound (*S*)-**1** (equivalent to 1× EC_{50} value) added, incubated at 37°C for 24 h and sterols extracted (lane 4); 2,3-oxidosqualene and compound (*S*)-**1** (equivalent to 3× EC_{50} value) added, incubated at 37°C for 24 h and sterols extracted (lane 5); 2,3-oxidosqualene and compound (*S*)-**1** (equivalent to 1× EC_{50} value) added, preparation incubated at 37°C for 24 h and sterols extracted (lane 6); 2,3-oxidosqualene and BIBX-79 (equivalent to 3× EC_{50} value) added, incubated at 37°C for 24 h and sterols extracted (lane 7). Bands representing lanosterol (OSC product) are indicated by red arrows.

(D) TPP melt curves for *L. donovani* OSC following incubation with compound (*S*)-**1** (red) or vehicle (0.1% DMSO, black). Data from technical replicates (circles and squares) are shown, and the mean shift in melting temperature (ΔT_m) for OSC was 3.4°C.

was derived from a multisequence alignment of several OSCs and squalene cyclases (SC) from different organisms. The human and parasite enzymes share 40% sequence identity and 56% sequence similarity. This level of homology is well within the range required to generate a model of sufficient quality to support ligand binding studies. In general, the overall sequence similarity of this class of enzymes is relatively low. However, these enzymes are characterized by a number of functionally relevant $QX_{2\text{-}5}GXW$ consensus sequence motifs that are present in their C-termini (QW motifs) (Poralla et al., 1994). These motifs were used to guide and verify the sequence alignments. The *N*-terminus of *L. donovani* OSC (up to the QW7 motif) aligns poorly with OSC and SC sequences from other organisms. Thus, our modeling efforts focused on the C-terminus of the protein, which, with the exception of a 50-amino acid insertion between QW6 and QW7, aligns well with *h*OSC, and encompasses the lanosterol binding site. Indeed, the lanosterol binding site is particularly well conserved. Specifically, of the 31 amino acids within 5 Å of lanosterol, 23 residues are identical (74%) and five of the remaining eight share similar properties (19%) (Figure S4).

The generated homology model was refined, optimized (see STAR methods), and used to investigate the binding modes of compounds (*S*)-1 and (*R*)-**1** by molecular docking. The *S* enantiomer generated the best docking score: −14.4 kcal·mol^{-1} compared with −12.7 kcal·mol^{-1} obtained for (*R*)-**1**. The top scoring poses for both enantiomers were used to evaluate changes in binding free energy (ΔG_{bind}). In keeping with our crystallographic and potency data, the *S* enantiomer was confirmed as having a higher affinity for the active site of OSC with a ΔG_{bind} of −80.7 kcal·mol^{-1} compared with a value of −73.9 kcal·mol^{-1} for (*R*)-**1**. The most favorable binding pose for (*S*)-**1** is characterized by formation of a salt bridge with the carboxylate of the catalytic Asp723 through the positively charged nitrogen atom of the methylimidazole moiety (Figure 5). The imidazole moiety of compound **1** has a calculated pK_a value ranging between 7.8 and 8.6, suggesting that it is protonated at physiological pH. The model indicates that this positive charge is

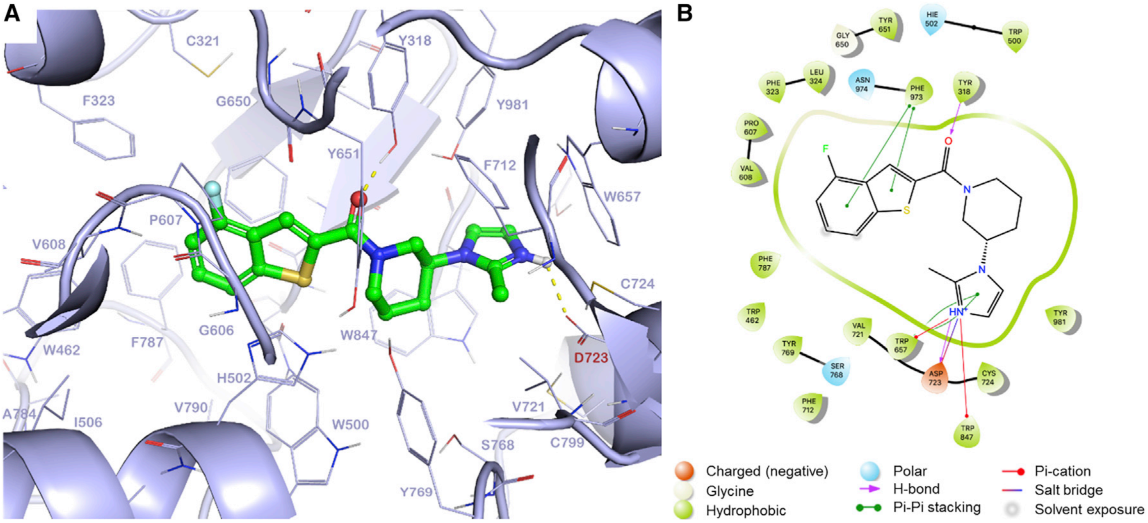

Figure 5. Docking of compound (*S*)-1 into a homology model of the *L. donovani* OSC active site

(A) Induced-fit docking pose of compound (*S*)-**1**. The ligand is represented as green sticks. The hydrogen bonds between the ligand and Asp723 and Tyr318 are shown as a yellow dotted line.

(B) 2D ligand interaction diagram based on the best-scoring docking pose for compound (*S*)-**1**.

further stabilized by π-cation interactions with the aromatic systems of Trp657 and Trp847. The carbonyl oxygen is hydrogen bonded to the Tyr318 side chain hydroxyl group and the aromatic systems at the extremes of the molecule are involved in two π-stacking interactions: the first between the methylimidazole ring and the indole ring of Trp657 and second between the benzothiophene moiety and the aromatic ring in Phe973 (Figure 5). It should be noted that our model of OSC indicates that residue Cys778, mutated to Phe in our resistant cell lines RES V, is between 12 and 19 Å from the lanosterol binding site. The remoteness of this mutation from the binding site is consistent with it playing no direct role in resistance to compound **1**.

Assessing the essentiality of OSC

Previous studies have provided conflicting evidence regarding the essentiality of enzymes involved in sterol biosynthesis across different *Leishmania* species. For instance, sterol 14α-demethylase (CYP51) has been demonstrated as essential for survival in *L. donovani* (McCall et al., 2015), but not in *L. major* (Xu et al., 2014). Here, our aim was to assess the essentiality of OSC in *L. donovani* promastigotes for the first time using a classical gene replacement strategy (Figure 6A). In the first instance, a single copy of *OSC* was successfully replaced in WT parasites with either a puromycin (*PAC*, puromycin *N*-acetyltransferase) or hygromycin (*HYG*, hygromycin phosphotransferase) drug resistance gene via homologous recombination. Attempts were made to directly replace the second allelic copy of *OSC* in these single-knockout cells (SKO) with either *HYG* or *PAC* directly or in cells constitutively expressing an ectopic copy of *OSC*. Despite recovering more than 40 putative nulls, resistant to selection with both hygromycin and puromycin, all of these clones retained a genomic copy of *OSC.* Indeed, both allelic copies of *OSC* were only successfully replaced in promastigotes bearing an ectopic copy of the gene (Figures 6B and S5). Our failure to replace both the endogenous copies of *OSC*, except in the presence of an ectopic copy of the gene, strongly suggests that this enzyme is essential for the promastigote stage of *L. donovani*. The current absence of a robust and reliable inducible-expression system in *Leishmania,* compatible with use intramacrophage, precludes our investigation of OSC essentiality in the more relevant amastigote stage of the parasite.

DISCUSSION

Our current study highlights the power of using orthogonal approaches to elucidate mechanisms of compound action. Several lines of evidence presented here establish OSC, a key enzyme of sterol biosynthesis, as the primary target of compound **1** and its enantiomers. WGS and screening of our genome-wide overexpression library confirmed that elevated levels of OSC in *Leishmania* promastigotes are associated with resistance to compound **1**. The generation of OSC-overexpressing parasites, through introduction of an ectopic copy of the gene into WT cells, led to a precipitous drop in susceptibility to compound **1**, thus confirming the direct role of this enzyme in resistance. Cells treated with these compounds accumulate significant amounts of 2,3-oxidosqualene, the established substrate of OSC. *L. donovani* membrane preparations supplemented with 2,3-oxidosqualene produced lanosterol, while this activity was ablated by incubation with the established OSC-specific inhibitor BIBX-79, as well as in the presence of compound (*S*)-**1**. Finally, TPP was used as an unbiased approach to confirm that compound (*S*)-**1** directly and specifically interacts with this enzyme of sterol biosynthesis.

Sterols perform a vital function in maintaining the structural integrity of cellular membranes. OSC catalyzes the most complex step in the production of mature sterols, the cyclization of 2,3-oxidosqualene to form lanosterol. In higher eukaryotes (including humans), the predominant sterol is cholesterol; however, the membranes of kinetoplastid parasites, such as

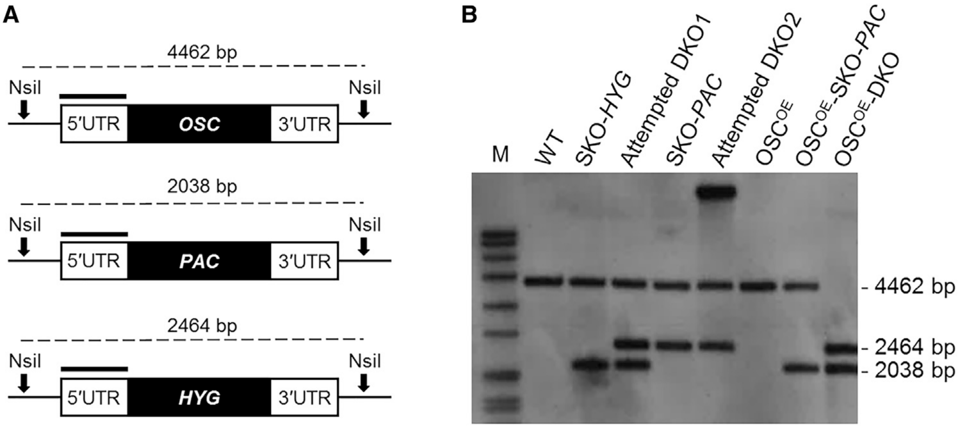

Figure 6. Assessing the essentiality of *OSC* in *L. donovani* promastigotes

(A) Schematic representation of the *OSC* locus in *OSC* single-knockout (HYG) and (PAC) cells. Black bars represent the 5′ UTR region upstream of the open reading frame of OSC, which was used as a probe in Southern blot analysis. NsiI sites with expected fragment sizes are shown.

(B) Southern blot analysis of NsiI-digested genomic DNA (~5 μg) from WT *L. donovani* (*Ld*BOB) cells, *OSC* single-knockout clones, attempted double knockout and a "rescued" DKO clone (OSC^{OE}-DKO) . The DIG-labeled 5′ UTR of *OSC* was used as a probe. This Southern blot was stripped and re-probed using a DIG-labeled fragment of OSC as a probe (see Figure S5).

Leishmania, more closely resemble those of fungi in composition, with ergosterol and ergostane-based sterols most abundant (Haughan et al., 1995). Indeed, kinetoplastids are unable synthesize cholesterol *de novo* (Roberts et al., 2003) and the different sterol composition of parasite membranes compared with that of their mammalian hosts has long been considered exploitable for drug discovery. Of particular note, posaconazole, which targets CYP51, the enzyme immediately downstream of OSC, was recently assessed in phase II clinical trials for Chagas disease (Molina et al., 2014). Despite initially positive results, 92% of patients taking part in this trial relapsed in the subsequent 10 to 12 months. Buckner and colleagues (Buckner et al., 2001; Hinshaw et al., 2003) have a long-standing interest in developing OSC inhibitors as antimicrobials, particularly targeting *T. cruzi*, with the most promising demonstrating low nM activity against the mammalian (amastigote) stage of the parasite. Several studies have demonstrated that *Leishmania* are susceptible to antifungal inhibitors of sterol biosynthesis, such as terbinafine (squalene epoxidase inhibitor) (Bahamdan et al., 1997; Berman and Gallalee, 1987; Goad et al., 1985) and ketoconazole (CYP51 inhibitor) (Berman, 1981; Berman et al., 1984). Ketoconazole and fluconazole, another CYP51 inhibitor, have shown promise for the treatment of cutaneous leishmaniasis in clinical trials (Alrajhi et al., 2002; Saenz et al., 1990). Collectively, these studies demonstrate the suitability of sterol biosynthesis as a target for anti-leishmanial drug discovery.

Despite being unable to synthesize cholesterol, *T. brucei*, *Leishmania* spp. and *T. cruzi* have demonstrated an ability to scavenge this sterol from their hosts (Coppens et al., 1988; De Cicco et al., 2012; Pereira et al., 2011). Indeed, uptake of exogenous cholesterol, via endocytosis of low-density lipoprotein (LDL) particles, is essential for the survival of bloodstream-form *T. brucei* (Coppens et al., 1995). The fact that kinetoplastids can use host-derived cholesterol raises the possibility that this may provide a route for parasites to circumvent the effects of sterol biosynthesis inhibition and reduce the efficacy of drugs targeting this metabolic pathway. In support of this hypothesis, studies with *L. amazonensis* showed that promastigotes treated with a variety of sterol biosynthesis inhibitors responded by increasing endocytosis of LDL (Andrade-Neto et al., 2011). Furthermore, the potency of these inhibitors could be somewhat modulated by varying access to exogenous cholesterol. In our current study, we did not observe this phenomenon, finding instead that varying levels of fetal calf serum in culture medium (5%–20%), an exogenous source of cholesterol, had little or no effect on the potency of compound **1** against promastigotes (Figure S6). It should also be noted that scavenged cholesterol alone cannot satisfy promastigote and amastigote sterol requirements (Roberts et al., 2003), thus *de novo* synthesis of ergosterol-based sterols is essential.

Compound **1** and its enantiomers demonstrate a notable drop-off in potency against axenic and intramacrophage amastigotes, compared with promastigotes. Initially, we hypothesized that this reduced potency may be due to the sparing effect of cholesterol scavenged from the macrophage, as discussed above. However, this would not explain the drop in potency seen with amastigotes cultured axenically in the absence of a host cell. The imidazole moiety of compound **1** has a calculated pK_a value range of between 7.8 and 8.6, suggesting that a proportion of this benzothiophene will be protonated at physiological pH. Intramacrophage amastigotes reside within acidified parasitophorous vacuoles at pH 5.5 and axenic amastigotes are cultured in media that closely mimics this environment. At pH 5.5, the levels of protonated compound **1** will be considerably higher than those in promastigote cultures (pH 7.4). This positively charged moiety is likely to adversely affect compound **1** permeability and thus the increased levels of protonation in amastigote cultures may be at least partially responsible for the observed drop in potency. With this in mind, one strategy to improve the potency of compound (*S*)-**1** could be to replace the methylimidazole with a bioisostere with a lower pK_a. For instance, the *h*OSC inhibitor BIBB515 has a dihydrooxazole moiety in this position (Lenhart et al., 2003) and a calculated pK_a value in the range of 5.44 to 4.72. Another potential strategy to

CellPress
OPEN ACCESS

improve potency is to exploit a hydrophobic channel within the active site of *Ld*OSC. Interestingly, both the terminal aliphatic chain of lanosterol and the *p*-bromo phenyl moiety of the *h*OSC inhibitor Ro 48-8071 extend in a hydrophobic channel that ultimately leads to the cell membrane (Thoma et al., 2004) (Figure S7). Based on our docking studies, positions 6 and 7 of the benzothiophene ring provide the right vector to exploit this channel and this has the potential to increase the potency of these inhibitors. Our intention is to investigate hydrophobic substituents to occupy this area.

In summary, these data confirm that compound **1** and its enantiomers specifically target OSC in *L. donovani*. We establish OSC as genetically essential for the survival of these parasites. Modeling and docking studies identify key interactions made between compound (*S*)-**1** and the OSC active site. In addition, we outline potential strategies to improve potency. Future studies should focus on evolving compounds within this series to achieve sub-μM potency against the mammalian stages of *L. donovani* prior to assessing the most promising compounds *in vivo*. It should be noted that compound **1** also demonstrates promising potency against intracellular *T. cruzi* (EC_{50} in the range 0.6–1 μM [Peña et al., 2015]). The failure of clinical trials with posaconazole for Chagas disease have left some in drug discovery reticent to pursue inhibitors of sterol biosynthesis. Nevertheless, future studies should also investigate the potential of these OSC-specific inhibitors for the treatment of American trypanosomiasis.

SIGNIFICANCE

Visceral leishmaniasis (kala-azar) is a serious vector-borne disease afflicting people, particularly in parts of Asia, Africa, and Latin America. There are approximately 300,000 new cases and an estimated 20,000 deaths each year, making it the world's second biggest parasitic killer after malaria. In 95% of cases, death can be prevented by timely and appropriate drug therapy; however, current treatments are far from ideal. Clinically used anti-leishmanials suffer from a number of serious issues, including the need for hospitalization, prolonged therapy, parenteral administration, high cost, variable efficacy, severe toxic side effects, and resistance. Thus, there is a pressing need for better, safer efficacious drugs that are fit-for-purpose in resource-poor settings. Unfortunately, anti-leishmanial drug discovery has been hindered by a lack of robustly validated drug targets in these parasites. This has limited target-focused screening programs and has increased reliance on phenotypic screening of parasites to identify start points for drug discovery. This approach has proved effective, however, a lack of information regarding the mechanism(s) of action or specific molecular target(s) of these active compounds can prove a barrier to the optimization of these early lead compounds. Here, we used multiple, unbiased approaches to identify the molecular target of a promising phenotypic hit as OSC, a key enzyme in sterol biosynthesis. Identifying the target of this benzothiophene enabled structure-focused strategies to improve potency to be proposed. Furthermore, this knowledge can inform future drug combinations and be exploited for *de novo*, target-based drug discovery. This illustrates the great value of comprehensive mechanism of action studies as an integrated part of a drug discovery program.

STAR★METHODS

Detailed methods are provided in the online version of this paper and include the following:

- KEY RESOURCES TABLE
- RESOURCE AVAILABILITY
  - Lead contact
  - Materials availability
  - Data and code availability
- EXPERIMENTAL MODEL AND SUBJECT DETAILS
  - Cell lines and culture conditions
- METHOD DETAILS
  - Chemistry
  - Chemistry - compound purification
  - Drug sensitivity assays
  - Cosmid library screening
  - Resistance generation
  - Whole genome sequencing and analysis
  - Generation of overexpression and knockout constructs
  - Generation of LdBOB transgenic cell lines
  - Southern blot analysis of transgenic cell lines
  - Protein quantification
  - LC-MS/MS analysis
  - Data analysis
  - Site-directed mutagenesis
  - Analysis of neutral lipids by thin-layer chromatography (TLC)
  - OSC cell-free assay
  - Homology modelling
  - Lysate production for thermal proteome profiling (TPP)
  - TPP assays
  - TPP sample processing and analysis
  - TPP data analysis
  - Induced-fit docking
  - Binding energies
  - pK_a calculations
- QUANTIFICATION AND STATISTICAL ANALYSIS

SUPPLEMENTAL INFORMATION

Supplemental information can be found online at https://doi.org/10.1016/j.chembiol.2021.02.008.

ACKNOWLEDGMENTS

We acknowledge Dr Alan Kennedy from the University of Strathclyde for small molecule crystallography of compound (*R*)-**1** and Erika Pinto for providing peritoneal macrophages for drug sensitivity studies. In addition, we thank Professor Terry Smith for help with preliminary lipidomic analysis. We also thank the Parasite Genomics group at the Wellcome Sanger Institute for use of the unpublished LdBPKLV9 genome. We thank the UK Medical Research Council and GlaxoSmithKline for the CASE PhD studentship awarded to Luciana Paradela. This work also was supported by a Wellcome Trust Center Award (203134/Z/16/Z) and a Wellcome Trust Strategic Award (105021).

AUTHOR CONTRIBUTIONS

Conceptualization, J.M., A.H.F., M.A.J.F., I.H.G., and S.W.; Methodology, L.S.P., S.P., M.L.S.G., F.Z., V.C.L., A.H.F., M.A.J.F., I.H.G., and S.W.; Experimental, L.S.P., R.J.W., S.C., G.C., V.C.L., M.L.S.G.; Resources, E.M., D.B., and S.P.; Supervision, S.P., F.Z., J.M., I.H.G., and S.W.; Writing, L.S.P., S.P., F.Z., and S.W.; Review and editing, SW.; Funding acquisition, J.M., I.H.G., and S.W.

DECLARATION OF INTERESTS

The authors declare no competing interests.

Received: October 14, 2020
Revised: January 25, 2021
Accepted: February 11, 2021
Published: March 09, 2021

REFERENCES

Aldasoro, E., Posada, E., Requena-Méndez, A., Calvo-Cano, A., Serret, N., Casellas, A., Sanz, S., Soy, D., Pinazo, M.J., and Gascon, J. (2018). What to expect and when: benznidazole toxicity in chronic Chagas' disease treatment. J. Antimicrob. Chemother. *73*, 1060–1067.

Alrajhi, A.A., Ibrahim, E.A., De Vol, E.B., Khairat, M., Faris, R.M., and Maguire, J.H. (2002). Fluconazole for the treatment of cutaneous leishmaniasis caused by Leishmania major. N. Engl. J. Med. *346*, 891–895.

Andrade-Neto, V.V., Cicco, N.N., Cunha-Junior, E.F., Canto-Cavalheiro, M.M., Atella, G.C., and Torres-Santos, E.C. (2011). The pharmacological inhibition of sterol biosynthesis in Leishmania is counteracted by enhancement of LDL endocytosis. Acta Trop. *119*, 194–198.

Bahamdan, K.A., Tallab, T.M., Johargi, H., Nourad, M.M., Ibrahim, K., el Sherbini, A.H., Karkashan, E., Khare, A.K., and Nauri, M.M. (1997). Terbinafine in the treatment of cutaneous leishmaniasis: a pilot study. Int. J. Dermatol. *36*, 59–60.

Barrett, M.P. (2018). The elimination of human African trypanosomiasis is in sight: report from the third WHO stakeholders meeting on elimination of gambiense human African trypanosomiasis. PLoS Negl. Trop. Dis. *12*, e0006925.

Berman, J.D. (1981). Activity of imidazoles against Leishmania tropica in human macrophage cultures. Am. J. Trop. Med. Hyg. *30*, 566–569.

Berman, J.D., and Gallalee, J.V. (1987). In vitro antileishmanial activity of inhibitors of steroid biosynthesis and combinations of antileishmanial agents. J. Parasitol. *73*, 671–673.

Berman, J.D., Holz, G.G., Jr., and Beach, D.H. (1984). Effects of ketoconazole on growth and sterol biosynthesis of Leishmania mexicana promastigotes in culture. Mol. Biochem. Parasitol. *12*, 1–13.

Brown, G.M., Millar, A.R., Masterson, C., Brimacombe, J.S., Nikolaev, A.V., and Ferguson, M.A. (1996). Synthetic phospho-oligosaccharide fragments of lipophosphoglycan as acceptors for Leishmania major alpha-D-mannosylphosphate transferase. Eur. J. Biochem. *242*, 410–416.

Buckner, F.S., Griffin, J.H., Wilson, A.J., and Van Voorhis, W.C. (2001). Potent anti-Trypanosoma cruzi activities of oxidosqualene cyclase inhibitors. Antimicrob. Agents Chemother. *45*, 1210–1215.

Carver, T., Harris, S.R., Berriman, M., Parkhill, J., and McQuillan, J.A. (2012). Artemis: an integrated platform for visualization and analysis of high-throughput sequence-based experimental data. Bioinformatics *28*, 464–469.

Childs, D., Bach, K., Franken, H., Anders, S., Kurzawa, N., Bantscheff, M., Savitski, M.M., and Huber, W. (2019). Nonparametric analysis of thermal proteome profiles reveals novel drug-binding proteins. Mol. Cell. Proteomics *18*, 2506–2515.

Coppens, I., Baudhuin, P., Opperdoes, F.R., and Courtoy, P.J. (1988). Receptors for the host low density lipoproteins on the hemoflagellate Trypanosoma brucei: purification and involvement in the growth of the parasite. Proc. Natl. Acad. Sci. U S A *85*, 6753–6757.

Coppens, I., Levade, T., and Courtoy, P.J. (1995). Host plasma low density lipoprotein particles as an essential source of lipids for the bloodstream forms of Trypanosoma brucei. J. Biol. Chem. *270*, 5736–5741.

Corpas-Lopez, V., Moniz, S., Thomas, M., Wall, R.J., Torrie, L.S., Zander-Dinse, D., Tinti, M., Brand, S., Stojanovski, L., Manthri, S., et al. (2019). Pharmacological validation of N-Myristoyltransferase as a drug target in leishmania donovani. ACS Infect. Dis. *5*, 111–122.

Croft, S.L., Sundar, S., and Fairlamb, A.H. (2006). Drug resistance in leishmaniasis. Clin. Microbiol. Rev. *19*, 111–126.

De Cicco, N.N., Pereira, M.G., Corrêa, J.R., Andrade-Neto, V.V., Saraiva, F.B., Chagas-Lima, A.C., Gondim, K.C., Torres-Santos, E.C., Folly, E., Saraiva, E.M., et al. (2012). LDL uptake by Leishmania amazonensis: involvement of membrane lipid microdomains. Exp. Parasitol. *130*, 330–340.

Don, R., and Ioset, J.R. (2014). Screening strategies to identify new chemical diversity for drug development to treat kinetoplastid infections. Parasitology *141*, 140–146.

Franken, H., Mathieson, T., Childs, D., Sweetman, G.M., Werner, T., Tögel, I., Doce, C., Gade, S., Bantscheff, M., Drewes, G., et al. (2015). Thermal proteome profiling for unbiased identification of direct and indirect drug targets using multiplexed quantitative mass spectrometry. Nat. Protoc. *10*, 1567–1593.

Glover, L., Alsford, S., Baker, N., Turner, D.J., Sanchez-Flores, A., Hutchinson, S., Hertz-Fowler, C., Berriman, M., and Horn, D. (2015). Genome-scale RNAi screens for high-throughput phenotyping in bloodstream-form African trypanosomes. Nat. Protoc. *10*, 106–133.

Goad, L.J., Holz, G.G., Jr., and Beach, D.H. (1985). Effect of the allylamine antifungal drug SF 86-327 on the growth and sterol synthesis of Leishmania mexicana mexicana promastigotes. Biochem. Pharmacol. *34*, 3785–3788.

Goyard, S., Segawa, H., Gordon, J., Showalter, M., Duncan, R., Turco, S.J., and Beverley, S.M. (2003). An in vitro system for developmental and genetic studies of Leishmania donovani phosphoglycans. Mol. Biochem. Parasitol. *130*, 31–42.

Haughan, P.A., Chance, M.L., and Goad, L.J. (1995). Effects of an azasterol inhibitor of sterol 24-transmethylation on sterol biosynthesis and growth of Leishmania donovani promastigotes. Biochem. J. *308* (Pt 1), 31–38.

Hinshaw, J.C., Suh, D.Y., Garnier, P., Buckner, F.S., Eastman, R.T., Matsuda, S.P., Joubert, B.M., Coppens, I., Joiner, K.A., Merali, S., et al. (2003). Oxidosqualene cyclase inhibitors as antimicrobial agents. J. Med. Chem. *46*, 4240–4243.

Jackson, Y., Alirol, E., Getaz, L., Wolff, H., Combescure, C., and Chappuis, F. (2010). Tolerance and safety of nifurtimox in patients with chronic chagas disease. Clin. Infect. Dis. *51*, e69–75.

Jafari, R., Almqvist, H., Axelsson, H., Ignatushchenko, M., Lundbäck, T., Nordlund, P., and Martinez Molina, D. (2014). The cellular thermal shift assay for evaluating drug target interactions in cells. Nat. Protoc. *9*, 2100–2122.

Langmead, B., and Salzberg, S.L. (2012). Fast gapped-read alignment with Bowtie 2. Nat. Methods *9*, 357–359.

Lenhart, A., Reinert, D.J., Aebi, J.D., Dehmlow, H., Morand, O.H., and Schulz, G.E. (2003). Binding structures and potencies of oxidosqualene cyclase inhibitors with the homologous squalene-hopene cyclase. J. Med. Chem. *46*, 2083–2092.

Li, H., Handsaker, B., Wysoker, A., Fennell, T., Ruan, J., Homer, N., Marth, G., Abecasis, G., and Durbin, R. (2009). The sequence alignment/map format and SAMtools. Bioinformatics *25*, 2078–2079.

Liang, Y., Besch-Williford, C., Aebi, J.D., Mafuvadze, B., Cook, M.T., Zou, X., and Hyder, S.M. (2014). Cholesterol biosynthesis inhibitors as potent novel anti-cancer agents: suppression of hormone-dependent breast cancer by the oxidosqualene cyclase inhibitor RO 48-8071. Breast Cancer Res. Treat. *146*, 51–62.

Liang, Y., Mafuvadze, B., Aebi, J.D., and Hyder, S.M. (2016). Cholesterol biosynthesis inhibitor RO 48-8071 suppresses growth of hormone-dependent and castration-resistant prostate cancer cells. Onco Targets Ther. *9*, 3223–3232.

Maione, F., Oliaro-Bosso, S., Meda, C., Di Nicolantonio, F., Bussolino, F., Balliano, G., Viola, F., and Giraudo, E. (2015). The cholesterol biosynthesis

CellPress
OPEN ACCESS

enzyme oxidosqualene cyclase is a new target to impair tumour angiogenesis and metastasis dissemination. Sci. Rep. *5*, 9054.

Mark, M., Muller, P., Maier, R., and Eisele, B. (1996). Effects of a novel 2,3-oxidosqualene cyclase inhibitor on the regulation of cholesterol biosynthesis in HepG2 cells. J. Lipid Res. *37*, 148–158.

McCall, L.I., El Aroussi, A., Choi, J.Y., Vieira, D.F., De Muylder, G., Johnston, J.B., Chen, S., Kellar, D., Siqueira-Neto, J.L., Roush, W.R., et al. (2015). Targeting Ergosterol biosynthesis in Leishmania donovani: essentiality of sterol 14 alpha-demethylase. PLoS Negl. Trop. Dis. *9*, e0003588.

Milletti, F., Storchi, L., Sforna, G., and Cruciani, G. (2007). New and original pKa prediction method using grid molecular interaction fields. J. Chem. Inf. Model. *47*, 2172–2181.

Molina, I., Gómez i Prat, J., Salvador, F., Treviño, B., Sulleiro, E., Serre, N., Pou, D., Roure, S., Cabezos, J., Valerio, L., et al. (2014). Randomized trial of posaconazole and benznidazole for chronic Chagas' disease. N. Engl. J. Med. *370*, 1899–1908.

Morand, O.H., Aebi, J.D., Dehmlow, H., Ji, Y.H., Gains, N., Lengsfeld, H., and Himber, J. (1997). Ro 48-8.071, a new 2,3-oxidosqualene:lanosterol cyclase inhibitor lowering plasma cholesterol in hamsters, squirrel monkeys, and minipigs: comparison to simvastatin. J. Lipid Res. *38*, 373–390.

Mueller, M., Ritmeijer, K., Balasegaram, M., Koummuki, Y., Santana, M.R., and Davidson, R. (2007). Unresponsiveness to AmBisome in some Sudanese patients with kala-azar. Trans. R. Soc. Trop. Med. Hyg. *101*, 19–24.

Pelfrene, E., Harvey Allchurch, M., Ntamabyaliro, N., Nambasa, V., Ventura, F.V., Nagercoil, N., and Cavaleri, M. (2019). The European Medicines Agency's scientific opinion on oral fexinidazole for human African trypanosomiasis. PLoS Negl. Trop. Dis. *13*, e0007381.

Peña, I., Pilar Manzano, M., Cantizani, J., Kessler, A., Alonso-Padilla, J., Bardera, A.I., Alvarez, E., Colmenarejo, G., Cotillo, I., Roquero, I., et al. (2015). New compound sets identified from high throughput phenotypic screening against three kinetoplastid parasites: an open resource. Sci. Rep. *5*, 8771.

Pereira, M.G., Nakayasu, E.S., Sant'Anna, C., De Cicco, N.N., Atella, G.C., de Souza, W., Almeida, I.C., and Cunha-e-Silva, N. (2011). Trypanosoma cruzi epimastigotes are able to store and mobilize high amounts of cholesterol in reservosome lipid inclusions. PLoS One *6*, e22359.

Perez-Riverol, Y., Csordas, A., Bai, J., Bernal-Llinares, M., Hewapathirana, S., Kundu, D.J., Inuganti, A., Griss, J., Mayer, G., Eisenacher, M., et al. (2019). The PRIDE database and related tools and resources in 2019: improving support for quantification data. Nucleic Acids Res. *47*, D442–D450.

Phillips, R.E., Yang, Y., Smith, R.C., Thompson, B.M., Yamasaki, T., Soto-Feliciano, Y.M., Funato, K., Liang, Y., Garcia-Bermudez, J., Wang, X., et al. (2019). Target identification reveals lanosterol synthase as a vulnerability in glioma. Proc. Natl. Acad. Sci. U S A *116*, 7957–7962.

Poralla, K., Hewelt, A., Prestwich, G.D., Abe, I., Reipen, I., and Sprenger, G. (1994). A specific amino acid repeat in squalene and oxidosqualene cyclases. Trends Biochem. Sci. *19*, 157–158.

Priotto, G., Kasparian, S., Mutombo, W., Ngouama, D., Ghorashian, S., Arnold, U., Ghabri, S., Baudin, E., Buard, V., Kazadi-Kyanza, S., et al. (2009). Nifurtimox-eflornithine combination therapy for second-stage African Trypanosoma brucei gambiense trypanosomiasis: a multicentre, randomised, phase III, non-inferiority trial. Lancet *374*, 56–64.

Riley, J., Brand, S., Voice, M., Caballero, I., Calvo, D., and Read, K.D. (2015). Development of a fluorescence-based trypanosoma cruzi CYP51 inhibition assay for effective compound triaging in drug discovery programmes for chagas disease. PLoS Negl. Trop. Dis. *9*, e0004014.

Roberts, C.W., McLeod, R., Rice, D.W., Ginger, M., Chance, M.L., and Goad, L.J. (2003). Fatty acid and sterol metabolism: potential antimicrobial targets in apicomplexan and trypanosomatid parasitic protozoa. Mol. Biochem. Parasitol. *126*, 129–142.

Rose, I.C., Sharpe, B.A., Lee, R.C., Griffin, J.H., Capobianco, J.O., Zakula, D., and Goldman, R.C. (1996). Design, synthesis and in vitro evaluation of pyridinium ion based cyclase inhibitors and antifungal agents. Bioorg. Med. Chem. *4*, 97–103.

Saenz, R.E., Paz, H., and Berman, J.D. (1990). Efficacy of ketoconazole against Leishmania braziliensis panamensis cutaneous leishmaniasis. Am. J. Med. *89*, 147–155.

Soto, J., and Soto, P. (2006). Miltefosine: oral treatment of leishmaniasis. Expert Rev. Anti Infect. Ther. *4*, 177–185.

Thoma, R., Schulz-Gasch, T., D'Arcy, B., Benz, J., Aebi, J., Dehmlow, H., Hennig, M., Stihle, M., and Ruf, A. (2004). Insight into steroid scaffold formation from the structure of human oxidosqualene cyclase. Nature *432*, 118–122.

Wall, R.J., Carvalho, S., Milne, R., Bueren-Calabuig, J.A., Moniz, S., Cantizani-Perez, J., MacLean, L., Kessler, A., Cotillo, I., Sastry, L., et al. (2020). The Q(i) site of cytochrome b is a promiscuous drug target in trypanosoma cruzi and leishmania donovani. ACS Infect. Dis. *6*, 515–528.

Wall, R.J., Moniz, S., Thomas, M.G., Norval, S., Ko, E.J., Marco, M., Miles, T.J., Gilbert, I.H., Horn, D., Fairlamb, A.H., et al. (2018). Antitrypanosomal 8-hydroxy-naphthyridines are chelators of divalent transition metals. Antimicrob. Agents Chemother. *62*, e00235-18.

Wyllie, S., Oza, S.L., Patterson, S., Spinks, D., Thompson, S., and Fairlamb, A.H. (2009). Dissecting the essentiality of the bifunctional trypanothione synthetase-amidase in Trypanosoma brucei using chemical and genetic methods. Mol. Microbiol. *74*, 529–540.

Wyllie, S., Patterson, S., Stojanovski, L., Simeons, F.R., Norval, S., Kime, R., Read, K.D., and Fairlamb, A.H. (2012). The anti-trypanosome drug fexinidazole shows potential for treating visceral leishmaniasis. Sci. Transl. Med. *4*, 119re111.

Xu, W., Hsu, F.F., Baykal, E., Huang, J., and Zhang, K. (2014). Sterol biosynthesis is required for heat resistance but not extracellular survival in leishmania. PLoS Pathog. *10*, e1004427.

Yun, O., Lima, M.A., Ellman, T., Chambi, W., Castillo, S., Flevaud, L., Roddy, P., Parreño, F., Albajar Viñas, P., and Palma, P.P. (2009). Feasibility, drug safety, and effectiveness of etiological treatment programs for Chagas disease in Honduras, Guatemala, and Bolivia: 10-year experience of Médecins Sans Frontières. PLoS Negl. Trop. Dis. *3*, e488.

STAR★METHODS

KEY RESOURCES TABLE

REAGENT or RESOURCE	SOURCE	IDENTIFIER
**Chemicals, peptides, and recombinant proteins**		
Compound 1	GSK	TCMDC-85 143498
Compound 1	Enamine	Z1139335838
BIBX-79	Enamine	Z1768160684
Posaconazole	Sigma Aldrich	Cat# SML2287
Resazurin	Sigma Aldrich	Cat# R7017
G418 disulfate salt	Sigma Aldrich	Cat# A1720
Puromycin *N*-acetyltransferase	Invitrogen	Cat# ant-pr-1
Hygromycin 180 phosphotransferase	Invitrogen	Cat# ant-hg-1
Nourseothricin antibiotic	Jena Bioscience	Cat# AB-101
BamHI-HF restriction enzyme	New England Biolabs	Cat# R3136
SwaI restriction enzyme	New England Biolabs	Cat# R0604
SmaI restriction enzyme	New England Biolabs	Cat# R0141
Octyl β-D-glucopyranoside	Sigma Aldrich	Cat# O8001
Nα-Tosyl-L-lysine chloromethyl ketone hydrochloride	Sigma Aldrich	Cat# T7254
cOmplete™, Mini, EDTA-free Protease Inhibitor Cocktail	Sigma Aldrich	Cat# 11836170001
Bradford Reagent	Supelco	Cat# B6916
2,3-Oxidosqualene	Sigma Aldrich	Cat# 41043
Lanosterol	Sigma Aldrich	Cat# L5768
Ergosterol	Sigma Aldrich	Cat# PHR1512
Methanol	Sigma Aldrich	Cat# 34860
Chloroform	VWR Chemicals	Cat# 22711.260
Iodine	Sigma Aldrich	Cat# 207772
Heptane CHROMASOLV™	Fischer Scientific	Cat# 34873
Diethyl ether	Fischer Scientific	Cat# D/2400/21
Acetic acid (glacial)	VWR Chemicals	Cat# 20104.334P
Lysyl Endopeptidase®, Mass Spectrometry Grade	Alpha Labs (Wako)	Cat# 125-02543
Trichloroacetic acid solution 6.1 N	Sigma-Aldrich	Cat# T0699
HPTLC silica gel 60	Supelco	Cat# 1055470001
**Critical commercial assays**		
Human T Cell Nucleofector™ Kit	Lonza	Cat# VPA-1002
RNeasy Mini Kit	Qiagen	Cat# 74104
Luna® Universal One-Step RT-qPCR Kit	New England Biolabs	Cat# E3005
PCR DIG Probe Synthesis Kit	Roche	Cat# 11636090910
TMT10plex™ Isobaric Mass Tagging Kit	Thermo	Cat# 90111
**Deposited data**		
Sequencing of genome-wide cosmid library screening	European Nucleotide Archive (ENA)	PRJEB37256
Whole genome sequencing of resistant cell lines	European Nucleotide Archive (ENA)	PRJEB37435
Mass spectrometry data	Proteomics Identification Database (PRIDE)	PXD023780
X-ray structural data	Cambridge Crystallographic Data Centre (CCDC)	2027159

(*Continued on next page*)

Continued		
REAGENT or RESOURCE	SOURCE	IDENTIFIER
Experimental models: cell lines		
LdBOB cosmid-based genome-wide overexpression library	(Corpas-Lopez et al., 2019)	N/A
*Ld*BOB (MHOM/SD/62/1S-CL2D) RES I-V clones	This paper	N/A
OE-*LdOSC*-pIR1SAT	This paper	N/A
OE-*LdHP*-pIR1SAT	This paper	N/A
KO-*Ld*OSC-PAC cassette	This paper	N/A
KO-*Ld*OSC-HYG cassette	This paper	N/A
Experimental models: organisms/strains		
*Leishmania donovani Ld*BOB (MHOM/SD/62/1S-CL2D)	(Goyard et al., 2003)	
Oligonucleotides		
Summarised in Tables S1 and S2	This paper	University of Dundee oligonucleotide synthesis service
Primer SDM-*LdOSC*-F 5′-GACA CGCGGCCCGGCGTACTTCGA GCTGCTGGACTGTGCGG-3′	*In house*	University of Dundee oligonucleotide synthesis service
Primer SDM-*LdOSC*-R 5′- CCGCACAG TCCAGCAGCTCGAAGTACG CCGGGCCGCGTGTC-3′	*In house*	University of Dundee oligonucleotide synthesis service
Oxidosqualene cyclase (*Ld*OSC, LdBPK.06.2.000670)	Commercial synthesis (GeneArt, Invitrogen)	Custom synthesis
Hypothetical protein (*Ld*HP, LdBPK.06.2.000680)	Commercial synthesis (GeneArt, Invitrogen)	Custom synthesis
Recombinant DNA		
pIR1-SAT plasmid	Kindly provided by Professor Stephen Beverley Washington University	N/A
Software and algorithms		
GRAFIT version 5.0.4	Erithacus Software	http://www.erithacus.com/grafit/
Artemis genome browser	Wellcome Sanger	https://www.sanger.ac.uk/tool/artemis/
MaxQuant software	Mav Plank Institute	http://maxquant.org/
Schrödinger suite (2019-3 release)	Schrödinger, LLC, New York, NY, 2020	https://www.schrodinger.com/
Moka - Molecular Discovery	(Milletti et al., 2007)	https://www.moldiscovery.com/software/moka/
Bowtie2	(Langmead and Salzberg, 2012)	http://bowtie-bio.sourceforge.net/bowtie2/index.shtml
Samtools v1.9	(Li et al., 2009)	http://www.htslib.org/
BCFtools v1.9	(Li et al., 2009)	https://samtools.github.io/bcftools/
Artemis v16.0.0	Wellcome Sanger	https://www.sanger.ac.uk/tool/artemis/
RITseq.py - Python script for sequence mapping	(Glover et al., 2015)	Nature methods
Adobe Illustrator CS5.1	Adobe	https://www.adobe.com/
Adobe Photoshop CS5.1	Adobe	https://www.adobe.com/
Inkscape vector graphics Editor 0.48	Inkscape	https://inkscape.org/
Excel 365	Microsoft	https://www.office.com/

RESOURCE AVAILABILITY

Lead contact

Further information and requests for resources should be directed to the Lead Contact, Susan Wyllie (s.wyllie@dundee.ac.uk).

Materials availability

Materials and reagents are available from the authors upon reasonable request.

Data and code availability

Genomic datasets generated during this study are available at European Nucleotide Archive [https://www.ebi.ac.uk/ena] deposited under the accession numbers PRJEB37256 and PRJEB37435, respectively. Proteomics datasets generated during this study are available at Proteomics Identification Database [https://www.ebi.ac.uk/pride/] deposited under the accession number PXD023780. X-ray structural data generated during this study are available at the Cambridge Crystallographic Data Centre [https://www.ccdc.cam.ac.uk/] deposited under the accession number 2027159.

EXPERIMENTAL MODEL AND SUBJECT DETAILS

Cell lines and culture conditions

A clonal *Leishmania donovani* cell line *Ld*BOB (derived from MHOM/SD/62/1S-CL2D) was grown as either promastigotes or axenic amastigotes in media-specific for each developmental stage, as previously described (Goyard et al., 2003). Axenic amastigotes were grown at 37°C in 5% CO_2 and promastigotes were grown at 28°C.

METHOD DETAILS

Chemistry

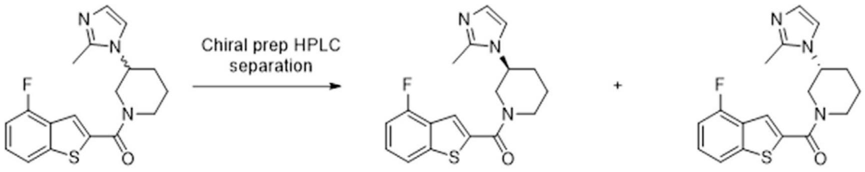

(*R*/*S*)-1 (*S*)-1 (*R*)-1

The enantiomers of (*R*/*S*)-1 were separated by chiral preparative-HPLC (performed by NEOMED Institute, Montréal). Full experimental details and analytical data for all compounds are shown below.

Absolute stereochemistry was determined by X-ray crystallography (Dr Alan Kennedy, University of Strathclyde, Glasgow). Briefly, a sample of the enantiomer that eluted first during HPLC purification was recrystallised by slow evaporation from heptane/toluene, and structural solution of the resultant crystals demonstrated that peak 1 was the *R* enantiomer. Crystallographic data were measured using a Bruker D8 Venture instrument. The structure was refined to convergence against F2 using all unique reflections and the program SHELXL-2014 as utilised within the WINGX GUI. All non-H atoms were refined anisotropically and H atoms were refined in riding modes, with the exception of H atoms in water molecules. These were refined independently and isotropically. Selected crystallographic data are presented in Table S1 and full structural details in cif format can be obtained from the Cambridge Crystallographic Data Centre (CCDC) via https://www.ccdc.cam.ac.uk/structures/. The database reference number is CCDC 2027159.

Chemistry - compound purification

^{1}H-NMR, ^{19}F-NMR, and 2D-NMR spectra were recorded on a Bruker Avance DPX 500 spectrometer (^{1}H at 500.1 MHz, ^{19}F at 470.5 MHz). Chemical shifts (δ) are expressed in ppm recorded using the residual solvent as the internal reference in all cases. Signal splitting patterns are described as singlet (s), doublet (d), triplet (t), quartet (q), multiplet (m), broad (br), or a combination thereof. LC-MS analyses were performed using an Agilent Technologies 1200 series HPLC connected to an Agilent Technologies 6130 quadrupole LC/MS connected to an Agilent diode array detector. High-resolution electrospray measurements were performed on a Bruker Daltonics MicrOTOF mass spectrometer.

(R/S)-(4-fluorobenzo[b]thiophen-2-yl)(3-(5-methyl-1H-imidazol-1-yl)piperidin-1-yl)methanone ((R/S)-1)

Purchased from Enamine (Product ID Z1139335838), off-white solid. MS (ES+): *m/z* (%) 344 (100) $[M+H]^+$, 687 (9) $[2M+H]^+$. HRMS (ES+): calcd. for $C_{18}H_{19}F_1N_3O_1S_1$ $[M+H]^+$ 344.1227, found 344.1233 (1.5 ppm).

Chiral separation of (R/S)-(4-fluorobenzo[b]thiophen-2-yl)(3-(5-methyl-1H-imidazol-1-yl)piperidin-1-yl)methanone ((R/S)-1)

The individual enantiomers of **(*R*/*S*)-1** (88 mg) were separated on a ChiralPak IA column (10×250 mm, 5 μm) connected to a Minigram semi-preparative SFC system (mobile phase 25:75 MeOH +10 mM ammonium formate: CO_2, 10 mL/min). The same HPLC system and conditions were used to determine the enantiomeric excess of each separated enantiomer.

CellPress
OPEN ACCESS

(*R*)-(4-fluorobenzo[*b*]thiophen-2-yl)(3-(5-methyl-1*H*-imidazol-1-yl)piperidin-1-yl)methanone (**(*R*)-1**) (peak 1, retention time 11.9 min, e.e. >99.7%): off-white solid (29 mg). Note, on standing the material would turn into a semi-solid, likely due to formation of the hydrate. ^{1}H-NMR (DMSO-d_6): δ 7.89 (d, 1H, *J*=8.5 Hz, ArH), 7.77 (d, 1H, *J*=0.5 Hz, ArH), 7.51-7.46 (m, 1H, ArH), 7.26 (dd, 1H, *J*=10.5, 8.5 Hz, ArH), 7.20 (br s, 1H, ArH), 6.76 (br s, 1H, ArH), 4.50-4.00 (m, 3H), 3.4-3.0 (m, 2H), 2.29 (s, 3H, CH_3), 2.10-2.04 (m, 1H, C*H*H), 2.03-1.95 (m, 1H, C*H*H), 1.89-1.82 (m, 1H, C*H*H), 1.76-1.67 (m, 1H, C*H*H). Note, many of the aliphatic peaks are broad probably due to restricted bond rotation. Note, residual formate from the HPLC purification was also observed. ^{19}F{^{1}H}-NMR (DMSO-d_6): δ -117.4. MS (ES+): *m/z* (%) 179 (76) [M- 3-(5-methyl-1*H*-imidazol-1-yl)piperidine]$^+$, 262 (39) [M- 5-methyl-1*H*-imidazole]$^+$, 344 (100) [M+H]$^+$.

(*S*)-(4-fluorobenzo[*b*]thiophen-2-yl)(3-(5-methyl-1*H*-imidazol-1-yl)piperidin-1-yl)methanone (**(*S*)-1**) (peak 2, retention time 15.5 min, enantiomeric excess. 98.6%): off-white solid (21 mg). Note, on standing the material would turn into a semi-solid, likely due to formation of the hydrate. ^{1}H-NMR (DMSO-d_6): δ 7.89 (d, 1H, *J*=8.5 Hz, ArH), 7.77 (d, 1H, *J*=0.5 Hz, ArH), 7.51-7.46 (m, 1H, ArH), 7.26 (dd, 1H, *J*=10.5, 8.5 Hz, ArH), 7.20 (br s, 1H, ArH), 6.76 (br s, 1H, ArH), 4.50-4.00 (m, 3H), 3.4-3.0 (m, 2H), 2.29 (s, 3H, CH_3), 2.09-2.05 (m, 1H, C*H*H), 2.03-1.95 (m, 1H, C*H*H), 1.88-1.83 (m, 1H, C*H*H), 1.76-1.67 (m, 1H, C*H*H). Note, many of the aliphatic peaks are broad most likely due to restricted bond rotation. Note, residual formate from the HPLC purification was also observed. ^{19}F {^{1}H}-NMR (DMSO-d_6): δ -117.4. MS (ES+): *m/z* (%) 179 (100) [M- 3-(5-methyl-1*H*-imidazol-1-yl)piperidine]$^+$, 262 (21) [M- 5-methyl-1*H*-imidazole]$^+$, 344 (15) [M+H]$^+$. $[\alpha]^{20}_{D}$ = +53.3 (c 1.00, CH_2Cl_2).

Chiral HPLC chromatograms and NMR spectra for (*R/S*)-1, (*R*)-1 and (*S*)-1 are available upon request.

Drug sensitivity assays

To examine the effects of compounds on parasite growth, promastigote and axenic amastigotes were seeded in 96-well plates at 5×10^4 and 2×10^5 parasites mL^{-1}, respectively. Parasites were exposed to test compounds over a range of concentrations (two-fold serial dilutions). Cells were incubated for 72 h, after which resazurin was added to each well to a final concentration of 50 μM before measuring fluorescence (excitation of 528 nm and emission of 590 nm), after a further 2-3 h incubation. Data were processed using GRAFIT (version 5.0.4, Erithacus Software) and fitted to a 2-parameter equation to determine the effective concentration inhibiting growth by 50% (EC_{50}):

$$y = \frac{100}{1 + \left(\frac{[I]}{EC_{50}}\right)^m}$$

In this equation, [*I*] represents the inhibitor concentration, and m is the slope factor. Experiments were performed at least in three independent biological replicates for each parasite species with the data presented as the weighted mean ± standard deviation. Intra-macrophage drug sensitivity assays were determined, as previously described (Wyllie et al., 2012).

Cosmid library screening

The construction of our cosmid-based genome-wide overexpression library in *L. donovani* has been described in detail previously (Corpas-Lopez et al., 2019). Here, cosmid-containing *L. donovani* promastigotes were maintained at a minimum concentration of 3.33×10^5 cells mL^{-1} (1.5×10^7 cells in total) in the presence of 125 μg mL^{-1} G418. Compound **1** was added to the library at an initial concentration equivalent to 2x the established EC_{50} value. Cell densities were monitored daily and the library was sub-cultured before reaching 1×10^7 mL^{-1}, with addition of fresh test compound. Resistant cells were harvested and cosmid DNA isolated as described (Corpas-Lopez et al., 2019). After purification, cosmid DNA (30 μg in 100 μL Tris-buffer) was sequenced using an Illumina HiSeq platform (Beijing Genomics Institute). Sequence reads were aligned to the *L. donovani* BPKLV9 genome sequence (v39.0, tritrypdb.org) and *L. donovani* BPK282A1 genome sequence (v39, tritrypdb.org) using Bowtie2 software (Langmead and Salzberg, 2012) with the following condition: very-sensitive-local. The aligned files were then manipulated using SAMtools (Li et al., 2009) and a custom python script to identify reads with the following barcodes: 5'-GCGGCCGCTCTAGAACTAGT-3' and 5'-CTCTTAAAAGCATCATGTCT-3' (for fragments in sense direction) or 5'-ACTAGTTCTAGAGCGGCCGC-3' and 5'-AGACATGATGCTTTTAAGAG-3' (for fragments in anti-sense direction). Reads were then quantified using the Artemis genome browser (Carver et al., 2012) and Excel then analysed as previously described (Corpas-Lopez et al., 2019). All associated datasets have been deposited with the European Nucleotide Archive under the following accession number: PRJEB37256.

Resistance generation

Compound-resistant cell lines were generated by subculturing a clone of wild-type *L. donovani* in the continuous presence of test compounds. Starting at sublethal concentrations, drug concentrations in 5 independent cultures were increased in a stepwise manner. When parasites were able to survive and grow in concentrations of compound **1** equivalent to >20x the established EC_{50} value, the resulting cell lines were cloned by limiting dilution in the presence of compound. Five clones (RES I–V) were selected for further biological study.

Whole genome sequencing and analysis

Genomic DNA was isolated from WT and resistant clones using a standard alkaline lysis protocol. Whole genomic sequencing was performed using a HiSeq4000 next-generation sequencing platform (Beijing Genomics Institute, Hong Kong) and the resulting data was analysed as described previously (Wall et al., 2020), except the newly released LdBPKLV9 genome was also used as a reference. All associated datasets have been deposited with the European Nucleotide Archive under the following accession number: PRJEB37435.

Generation of overexpression and knockout constructs

Oxidosqualene cyclase (*LdOSC*, LdBPK.06.2.000670) and hypothetical protein (*LdHP*, LdBPK.06.2.000680) overexpression constructs were assembled by inserting synthetic versions of each gene (GeneArt, Invitrogen) into the pIR1SAT vector via a BamHI site. The resulting overexpression constructs (OE-*LdOSC* or OE-*LdHP*-pIR1SAT, respectively) were sequenced in-house to confirm their accuracy. Primers used in sequencing are summarised in Table S2.

LdOSC gene replacement cassettes were synthesised, comprising the selectable drug resistance genes puromycin *N*-acetyltransferase (*PAC*) or hygromycin phosphotransferase (*HYG*) flanked by the 470 bp immediately upstream and downstream of *LdOSC* gene. The accuracy of the resulting cassettes (KO-*LdOSC*-PAC and KO-*LdOSC*-*HYG*) were again verified by sequencing. Primers used in sequencing are summarised in Table S2.

Generation of LdBOB transgenic cell lines

Overexpression constructs were linearised with SwaI and knockout constructs were digested with SmaI prior to transfection. Mid-log-promastigotes (2×10^7 cells in total) were transfected with 5 – 10 μg of overexpression or knockout constructs using the Human T-Cell Nucleofector kit and Amaxa Nucleofector electroporator (program V-033). Following transfection, cells were allowed to recover for 16-24 h, before the appropriated drug selection (nourseothricin 100 μg mL^{-1}, hygromycin 50 μg mL^{-1} and puromycin 20 μg mL^{-1}). Cloned cell lines were generated by limiting dilution, maintained in selective medium and removed from drug selection for one passage prior to experiments.

Southern blot analysis of transgenic cell lines

The ORF and 5′ UTR of *OSC* were amplified by PCR (using the primers listed in Table S2) with the PCR DIG Probe Synthesis kit (Roche). The resulting digoxigenin (DIG)-labelled products were used as probes and Southern-blot analysis was carried out as previously described (Wyllie et al., 2009).

Protein quantification

L. donovani promastigotes WT and transgenic cell lines confirmed as overexpressing OSC and HP were grown for 72 h in roller bottles, starting at an initial concentration of 1×10^5 cells mL^{-1} (1.5×10^7 cells in total). Mid-log phase promastigotes were washed with ice-cold PBS and harvested by centrifugation (1912 g, 15 min, 4°C). The cell pellets were resuspended in 8 mL of ice-cold lysis buffer (1 mM EDTA, 1 mM DTT, 100 μM TLCK, and 1× Roche EDTA-free cOmplete protease inhibitor cocktail in 50 mM potassium phosphate buffer, pH 7.4), submitted to 3 freeze−thaw cycles in a dry ice/ethanol bath to biologically inactivate the parasites and followed by cell disruption (Constant Systems, UK) at 30 kpsi. The resulting lysates were centrifuged (100,000 g, 20 min, 4 °C), supernatants were collected, and the protein concentrations were determined using the Bio-Rad Protein Assay.

LC-MS/MS analysis

Analysis of peptides was performed on a Q Exactive™ plus, Mass Spectrometer (Thermo Scientific) coupled to a Dionex Ultimate 3000 RS (Thermo Scientific). The following buffers were used: Buffer A (0.1% formic acid in Milli-Q water (v/v)) and Buffer B (80% acetonitrile and 0.1% formic acid in Milli-Q water (v/v)). Samples (15 μL) were loaded at 10 μL min^{-1} onto a trap column (100 μm × 2 cm, PepMap nanoViper C18 column, 5 μm, 100 Å, Thermo Scientific) that had been pre-equilibrated with Buffer A (98%). The trap column was then washed for 5 min and switched in-line with a resolving C18 column (75 μm × 50 cm, PepMap RSLC C18 column, 2 μm, 100 Å; Thermo Scientific). Peptides were eluted from the column at a constant flow rate of 300 nL min^{-1} with a linear gradient of 2-35% Buffer B over 125 min, followed by 98% Buffer B for 127 min. The column was then washed with 98% Buffer B for 20 min prior to equilibration in 2% Buffer B for 17 min. The Q Exactive plus was used in data-dependent mode. The scan cycle comprised MS1 scan (m/z range from 335-1600, with a maximum ion injection time of 20 ms, a resolution of 70 000 and automatic gain control (AGC) value of 1×10^6) followed by 15 sequential dependent MS2 scans (with an isolation window set to 1.4 Da, resolution at 17500, maximum ion injection time at 100 ms and AGC 2×10^5). Stepped collision energy was set to 27 and fixed first mass to 100 m/z. The spectrum was acquired in centroid mode and unassigned charge states, charge states above 6, as well as singly charged species, were rejected. To ensure mass accuracy, the Q Exactive plus was calibrated on the day of analysis. LC-MS analysis was performed by the FingerPrints Proteomics Facility (University of Dundee).

Data analysis

MS data analysis was performed using MaxQuant software (http://maxquant.org/, version 1.6.2.6a). Carbamidomethyl (C), oxidation (M), acetyl (Protein N-term), deamidation (NQ) and Gln-> pyro-Glu were set as a variable modification. Proteins were identified by searching a protein sequence database containing *L. donovani* BPK282A1 annotated proteins (downloaded from TriTrypDB 46,

CellPress
OPEN ACCESS

http://www.tritrypdb.org). Label-free quantitation (LFQ) and "match between runs" features were enabled. Trypsin/P and LysC/P were selected as the digestive enzymes with two potential missed cleavages. The FDR threshold for peptides and proteins was 0.01. FTMS MS/MS mass tolerance was set to 10 ppm and ITMS MS/MS mass tolerance was 0.6 Da. Protein abundance was obtained from LFQ intensity values. LFQ intensities were calculated using at least 2 unique peptides. Data was visualised using Perseus 1.6.2.1 (https://maxquant.org/perseus/).

Site-directed mutagenesis

The custom-synthesised plasmid pMS containing *LdOSC* gene was used as a template for site-directed mutagenesis using the QuikChange II Site-Directed Mutagenesis Kit (Agilent), as per manufacturer's instructions. Complimentary primers were designed to generate a single nucleotide polymorphism (G2333T): SDM-*LdOSC*-F 5′-GACACGCGGCCCGGCGTACTTCGAGCTGCTGGACTGTGCGG-3′ and SDM-*LdOSC*-R 5′-CCGCACAGTCCAGCAGCTCGAAGTACGCCGGGCCGCGTGTC-3′. PCR conditions were as follows: 1 cycle of 30 s at 95°C; and 16 cycles of 30 s at 95°C, 1 min at 55°C and 5 min at 68°C. The accuracy of the mutagenesis was confirmed by sequencing.

Analysis of neutral lipids by thin-layer chromatography (TLC)

Promastigotes were incubated with compound **1,** compound (*S*)-**1,** BIBX-79 or posaconazole at concentrations equivalent to 3x their respective EC_{50} values. Depending on the level of compound treatment, the starting cell density of cultures varied between 1×10^3 cells mL^{-1} and 8×10^5 cells mL^{-1}. After 96 h, 50 mL of each culture (mid-log) were washed 2× with phosphate-buffered saline (3000*g*, 5 min, RT). Resulting pellets were resuspended in 1:2:0.8 parts of chloroform:methanol:water (v/v/v), homogenised and left at room temperature overnight for extraction. The supernatant enriched with lipids was harvested following centrifugation (3000*g,* 20 min), transferred to a new tube and subjected to a second round of extraction. The lower phase containing the total pool of lipids was collected and the extract volume corresponding to 1×10^8 cells was dried under nitrogen.

Extracted lipids were dissolved in 30 μL chloroform, spotted onto HPTLC silica gel 60 plates (Sigma) and allowed to migrate in heptane: ethyl ether: acetic acid (85:15:1, v/v/v). Ergosterol, lanosterol and 2,3-oxidosqualene standards (1 mg mL^{-1}) were run in parallel. Following separation, the unsaturated double bonds of lipids were stained with iodine vapour.

OSC cell-free assay

Mid-log promastigotes were harvested by centrifugation (3000*g*, 10 min, RT) and washed once with ice-cold phosphate-buffered saline. Membrane-enriched lysates were prepared by nitrogen cavitation using a pre-chilled 45 mL Parr cell disruption vessel (406 PSI, 4°C), as described (Brown et al., 1996). Aliquots of membrane-enriched fractions (equivalent to 3 $x10^8$ cells) were suspended in Buffer A (50 mM Hepes pH 7.4, 25 mM KCl, 5 mM $MgCl_2$, 5 mM $MnCl_2$, 0.1 mM $TosLysCH_2Cl$, 1 μg/mL leupeptin) supplemented with 20% glycerol, flash-frozen in liquid nitrogen and stored at −80°C. Prior to assay, aliquots were defrosted on ice then diluted in Buffer A supplemented with 0.8% of octyl β-D-glucopyranoside to the final volume of 600 μL. Substrate (2,3-oxidosqualene, 6 μg) and/or test compounds (*S*)-1 and BIBX-79 were added at concentrations equivalent to 1, 3 or 10× their respective EC_{50} values. Following incubation for 24h at 37 °C, lipids were extracted from each sample, spotted onto HPTLC silica gel and stained with iodine vapour, as previously described.

Homology modelling

The full amino acid sequence of *Ld*OSC (E9B8S7) was used to identify homologues through a BLAST homology search. The human OSC (*h*OSC: P48449) demonstrated 40% sequence identity and 58% similarity to the parasite enzyme; and was chosen as a suitable template for homology modelling. A 2.1Å crystal structure of *h*OSC complexed with lanosterol was used as the template structure (PDB ID: 1W6K). In this structure, a lanosterol molecule is fully enclosed within the enzyme active site. To improve the modelling of the enzyme binding site, lanosterol was also modelled into the active site of *Ld*OSC. A multi-sequence alignment of 10 squalene and oxidosqualene cyclases from different organisms was used to derive the *Ld*-human OSC sequence alignment used to build the homology model. The alignment was further manually curated to move an insertion from an α-helix into a loop region. The *LdOSC* model was generated and optimised using the protein prediction algorithm Prime and other tools available in the Schrödinger modelling platform (Schrödinger Release 2019-3: Schrödinger, LLC, New York, NY, 2020). After an initial restrained minimization using the Protein Preparation Wizard tool, non-template loops were further minimised using the Refine Loops tool. The hydrogen bonding network of the whole model was optimised reorienting the hydroxyl (Ser, Tyr), thiol (Cys) and the amide groups (Asn and Gln), as well as the imidazole rings (His). In addition, the predicted protonation states of His, Asp and Glu, and tautomeric states of His were also optimised. VSGB Solvation Model and the OPLS3e force field were used to minimise strain in the structure and to adjust the placement of various groups.

Lysate production for thermal proteome profiling (TPP)

L. donovani promastigotes (5×10^7 parasites mL^{-1}) were incubated in the presence of compound (*S*)-1 (equivalent to 10× the established EC_{50} value) or vehicle (0.1% (v/v) DMSO) for 3h at 28°C. Parasites were then harvested (1912 g, 15 min, 4°C), washed once with ice-cold phosphate-buffered saline and again harvested. The resulting cell pellets were resuspended in 8 mL of ice-cold lysis buffer (1 mM EDTA, 1 mM DTT, 100 μM TLCK, 0.8% octyl β-D-glucopyranoside and 1× Roche EDTA-free cOmplete protease inhibitor cocktail in 50 mM potassium phosphate buffer, pH 7.4) and then submitted to 3 freeze−thaw cycles in order to biologically inactivate

the parasites. Cells were then lysed using the Constant Systems cell disruptor at 30 kpsi. The resulting lysates were centrifuged (100,000 g, 20 min, 4 °C), supernatants collected, and protein concentrations determined, using the Bio-Rad Protein Assay. It should be noted that each step of this process was carried out in the constant presence of compound (*S*)-1 (10 × EC_{50}) or DMSO, as appropriate.

TPP assays

Lysates were adjusted to a final concentration of 1 mg/mL with Lysis Buffer. Aliquots (2 x 2 mL) were incubated at room temperature for 30 min in the presence of compound 1 (*S*) (10x EC_{50} value) or vehicle (0.1% DMSO). Each 2 mL aliquot (drug and vehicle treated) was further divided into 10 x 100 μL aliquots in 0.5 mL Protein LoBind tubes (Eppendorf) and incubated at the following temperatures (33, 37, 41, 45, 49 , 53, 57, 61, 65 and 69°C) for 3 min, followed by incubation at RT for 3 min. Each sample was then placed on ice for 15 min, centrifuged (100,000 g, 20 min, 4°C), supernatants harvested, and protein concentrations determined.

TPP sample processing and analysis

All aspects of sample processing, fractionation by HPLC, LC-MS/MS, peptide and protein identification and quantitation were described previously (Corpas-Lopez et al., 2019).

TPP data analysis

TPP experiments were analysed using the TPP Package available in Bioconductor, as previously described (Corpas-Lopez et al., 2019; Franken et al., 2015). Briefly, raw protein abundance, calculated from normalized reporter ion intensities of all quantified proteins, were log transformed and scaled between 0 and 1 by subtracting the global minimum and normalizing to the abundance at the lowest temperature of each protein to yield fold changes. The melting curves were calculated using a sigmoidal fitting approach with the TPP R package. This fitting was used to determine the melting point (T_m), defined as the temperature at which half of all proteins were denatured. The melting point differences (ΔT_m) were calculated by subtracting the T_m values of treated and untreated sample. The sigmoidal melting curves were filtered according to the following criteria: melting curves must reach a relative abundance plateau <0.3 and the coefficient of determination (R2) must be >0.8. In addition, non-parametric analysis of response curves (NPARC) was performed (Childs et al., 2019). This procedure is based on non-parametric statistics, comparing two models (treated and control curves) by their goodness of fit, allowing the detection of treatment-induced changes in the absence of the melting point, the central parameter of the standard method. Proteins with a FDR-adjusted *p*-value <0.01 in one biological replicate are considered hits. The mass spectrometry proteomics data have been deposited to the ProteomeXchange Consortium via the PRIDE (Perez-Riverol et al., 2019) partner repository with the dataset identifier PXD023780.

Induced-fit docking

Low energy conformers for both enantiomers of compound **1** were generated using LigPrep in the Schrödinger platform. The protonation state of each enantiomer at pH 7.4 was defined by MoKa from the Molecular Discovery suite (Milletti et al., 2007). The prepared ligands were then docked into the binding pocket of the *Ld*OSC model using the Schrödinger induced-fit docking protocol that uses Glide, OPLS3e force field and Prime to minimize the poses in the binding site obtained for compound **1**. A 20 Å cubic box centred on the centroid of the lanosterol ligand was used to generate the docking grid. No distance or hydrogen bond constraints were applied. The default settings were modified to increase the conformational sampling of the aliphatic rings of each ligand by increasing the energy window to 2.5 kcal mol^{-1} and the non-planar conformation of the amide bonds were penalised. For the Prime Refinement options the residues to be refined were at 6.0 Å from the ligand poses and no other residues were selected. For the redocking options the Extra-Precision method was selected (XP).

Binding energies

The molecular mechanics energies, combined with the generalised Born and surface area continuum solvation (MM-GBSA), were calculated using the following equation in Prime from Schrödinger with the VSGB Solvation model and OPLS3e force field:

$$\Delta G_{bind} = E_{complex(minimised)} - E_{ligand(minimised)} - E_{receptor(minimised)}$$

The Minimise Sampling method was also applied.

pK_a calculations

A variety of different software packages including SciFinder (scifinder.cas.org), MoKa (https://www.moldiscovery.com/software/moka/) and Epik (https://www.schrodinger.com/epik) were used to calculate pK_a values for compounds within this study.

QUANTIFICATION AND STATISTICAL ANALYSIS

All potency data were analysed in GraFit using their 2- parameter fit. Details of replicates and data analysis for each experiment can be found in the figure legends. Label-free quantitative proteomics experiments were analysed using MaxQuant software (http://maxquant.org/, version 1.6.2.6a). The FDR threshold for peptides and proteins was 0.01. FTMS MS/MS mass tolerance was set

to 10 ppm and ITMS MS/MS mass tolerance was 0.6 Da. Protein abundance was obtained from LFQ intensity values. LFQ intensities were calculated using at least 2 unique peptides. Thermal proteome profiling experiments were analysed using the TPP Package available in Bioconductor. Melt curves were calculated using a sigmoidal fitting approach with the TPP R package. Non-parametric statistical analysis of response curves (NPARC) (Childs et al., 2019) involves the comparison two models (treated and control curves) by their goodness of fit, allowing the detection of treatment-induced changes in the absence of the melting point, the central parameter of the standard method. Proteins with a FDR-adjusted *p*-value <0.01 in one biological replicate were considered hits.

Cell Chemical Biology, Volume *28*

Supplemental information

Multiple unbiased approaches identify oxidosqualene cyclase as the molecular target of a promising anti-leishmanial

Luciana S. Paradela, Richard J. Wall, Sandra Carvalho, Giulia Chemi, Victoriano Corpas-Lopez, Eoin Moynihan, Davide Bello, Stephen Patterson, Maria Lucia S. Güther, Alan H. Fairlamb, Michael A.J. Ferguson, Fabio Zuccotto, Julio Martin, Ian H. Gilbert, and Susan Wyllie

Supplementary information

Table S1. Selected crystal data and structural refinement for peak 1 isolated from chiral separation of compound 1 (racemate). Related to Figure **1** and table **1**.

Identification code	DDD01712564	
Empirical formula	C18 H20 F N3 O2 S	
Formula weight	361.43	
Temperature	104(2) K	
Wavelength	1.54178 Å	
Crystal system	Hexagonal	
Space group	P 61	
Unit cell dimensions	a = 10.1357(1) Å	α= 90°.
	b = 10.1357(1) Å	β= 90°.
	c = 28.9939(6) Å	γ = 120°.
Volume	2579.55(7) $Å^3$	
Z	6	
Density (calculated)	1.396 Mg/m^3	
Absorption coefficient	1.913 mm^{-1}	
F(000)	1140	
Crystal size	0.075 x 0.075 x 0.015 mm^3	
Theta range for data collection	5.038 to 72.163°.	
Index ranges	-12<=h<=11, -12<=k<=12, -35<=l<=35	
Reflections collected	59548	
Independent reflections	3393 [R(int) = 0.0532]	
Completeness to theta = 70.000°	100.0 %	
Absorption correction	Semi-empirical from equivalents	
Max. and min. transmission	0.4990 and 0.3414	
Refinement method	Full-matrix least-squares on F^2	
Data / restraints / parameters	3393 / 1 / 235	
Goodness-of-fit on F^2	1.112	
Final R indices [I>2sigma(I)]	R1 = 0.0243, wR2 = 0.0569	
R indices (all data)	R1 = 0.0256, wR2 = 0.0577	
Absolute structure parameter	0.008(5)	
Extinction coefficient	n/a	
Largest diff. peak and hole	0.142 and -0.184 e.$Å^{-3}$	

Table S2. Summary of primers used in OSC gene replacement studies. Related to figure **6.**

**Primers**	**Sequence**
KO-*OSC*-F	5′-GCTCTACACAGAGCCATACGATGC-3′
KO-*PURO*-F	5′-GCTGCAAGAACTCTTCCTCACG-3′
KO-*HYG*-F	5′-CGTCTGTCGAGAAGTTTCTGATCG-3′
KO-3′ UTR-R	5′-AGGTAGAGTGTGACGTGAGAACG-3′
Probe-*OSC*-F	5′-CGTGAATGGGCTCACCACCAGC-3′
Probe-*OSC*-R	5′-CGAGTGCTTGTGCACATTGGC-3′
Probe-5′ UTR-F	5′-ATCTTGTTGCCCTTCACCAGC-3′
Probe-5′ UTR-R	5′-ACTTTCCTCGCGCGTCTTTCC-3′

Table S3. Collated EC_{50} data for WT, resistant and transgenic cell lines. Related to figures **2** and **3.**

Cell line	EC_{50} values, µM (fold change versus WT)							
	Compound **1**		(*S*)-**1**		(*R*)-**1**		BIBX-79	
Wild-type	0.5 ± 0.02	(-)	0.4 ± 0.01	(-)	3 ± 0.1	(-)	0.5 ± 0.01	(-)
RES I	26 ± 3	(51)	10 ± 2	(26)	> 50	(> 19)	24 ± 7	(47)
RES2	13 ± 0.8	(26)	6 ± 0.2	(16)	43 ± 2	(16)	7 ± 0.5	(13)
RES3	14 ± 2	(28)	6 ± 0.8	(16)	> 50	(> 19)	6 ± 0.5	(11)
RES4	10 ± 2	(19)	4 ± 0.7	(11)	> 50	(> 19)	8 ± 0.5	(15)
RES5	17 ± 1	(34)	8 ± 1	(21)	> 50	(> 19)	8 ± 2	(15)
OSC^{OE}	5 ± 0.1	(10)	4 ± 0.1	(10)	30 ± 2	(11)	5 ± 0.3	(10)
HP^{OE}	0.7 ± 0.03	(1.4)	0.5 ± 0.1	(1)	3 ± 0.5	(1.3)	0.4 ± 0.001	(0.8)

All EC_{50} values represent the weighted mean ± standard deviation of at least three biological replicates (n ≥ 3) with each biological replicate comprised of two technical replicates.

Table S4. Summary of encoding SNPs identified in compound 1-resistant clones.

Related to figure **2.**

**Protein**	**Gene ID**	**Encoding mutation**	**Resistant clone**	**Zygosity**
Oxidosqualene cyclase	LdBPK.06.2.000670	C773F	V	Het
Hypothetical protein, conserved	LdBPK.08.2.000410	R1099G	IV	Het
Protein tyrosine kinase, putative	LdLV9.15.2.203710	Q607H	I	Het
HEAT repeats, putative	LdBPK.18.2.000670	P264S	III	Het
Protein of unknown function (DUF2946), putative	LdBPK.20.2.000420	R165M	V	Het
PRO8NT (NUC069), PrP8 N-terminal domain	LdBPK.35.2.004000	A2318V	III	Het

Table S5. Genes encoded by the 54.5 kb fragment of chromosome 6 enriched in compound 1-resistant parasites following selection of the cosmid library. 87% of all of all reads mapped to this genomic location. Genes common to all enriched fragments are highlighted in light blue. Related to figure **2**.

			LdBPKLV9 reads		LdBPK282A1 reads	
**LdBPKLV9 gene ID**	**LdBPK282A1 gene ID**	**Gene name**	**RPKM**	**Total**	**RPKM**	**Total**
LdBPK.06.2.000630	LdBPK_060630	Carbonic anhydrase family protein, putative	1342.3	6990	1421.2	7502
LdBPK.06.2.000640	LdBPK_060640	Putative phosphatase/protein of unknown function DUF89	6970.8	119545	7018.9	122016
LdBPK.06.2.000650	LdBPK_060650	Hypothetical protein, unknown function	16301.7	528431	16190.1	531992
LdBPK.06.2.000660	LdBPK_060660	Hypothetical protein, conserved	23923.4	1275852	23663.1	1282076
LdBPK.06.2.000670	LdBPK_060670	Oxidosqualene cyclase	25915.7	440919	25529.5	442484
LdBPK.06.2.000680	LdBPK_060680	Hypothetical protein, conserved	24303.2	280329	24054.8	281258
LdBPK.06.2.000690	LdBPK_060690	Hypothetical protein, conserved	17752.3	392670	17687.6	396589
LdBPK.06.2.000700	LdBPK_060700	Hypothetical protein, conserved	17700.8	73262	17693.5	74233
LdBPK.06.2.000710	LdBPK_060710	Ctf8, putative	13594.1	94082	13523.3	94872
LdBPK.06.2.000720	LdBPK_060720	Hypothetical protein, conserved	4552.9	203268	4577.5	207161

Table S6. List of top 10 proteins demonstrating thermal shift in the presence of compound (*S*)-1 in technical replicates. These proteins were identified as "hits" or potential targets by NPARC analysis. The criteria used to determine NPARC hits is described in STAR methods. Related to figure **4**.

Gene ID	Encoded protein	NPARC *p*-value	ΔT_m (1)	ΔT_m (2)
LdBPK_070460	hypothetical protein - conserved	1.2E-11	-9.71	-8.55
LdBPK_151590	hypothetical protein - conserved	1.6E-10	T >69	T >69
LdBPK_360230	SET domain protein - putative	4.6E-09	5.63	4.78
LdBPK_091220	hypothetical protein - conserved	1.7E-07	-13.39	C >69
LdBPK_060670	oxidosqualene cyclase	4.9E-07	3.83	2.93
LdBPK_210770	ATP-binding cassette protein subfamily E - member 1 - putative	7.6E-07	3.42	3.92
LdBPK_261880	Leucine Rich repeat - putative	7.6E-07	3.98	5.13
LdBPK_190690	kinesin - putative	4.1E-06	T >69	T >69
LdBPK_200750	hypothetical protein - conserved	4.9E-06	6.34	7.65
LdBPK_090160	FHA domain/Ring finger domain/Zinc finger - C3HC4 type (RING finger) containing protein - putative	9.3E-06	9.43	2.67

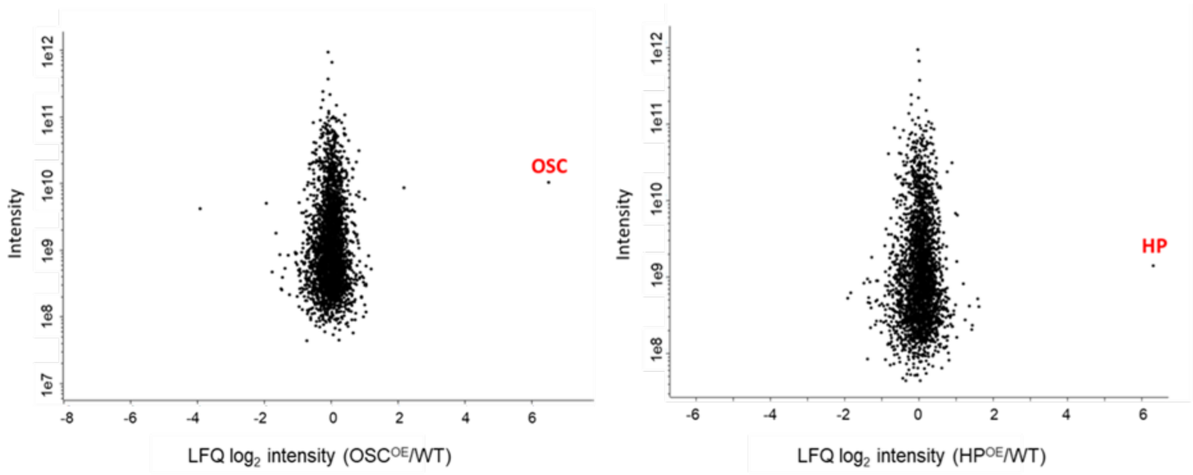

Figure S1. Label-free proteomics quantitation of OSC and HP transgenic cell lines. Relative levels of proteins in WT and transgenic cell lines were compared. Details of this analysis can be found in the materials and methods. Related to figure **3**.

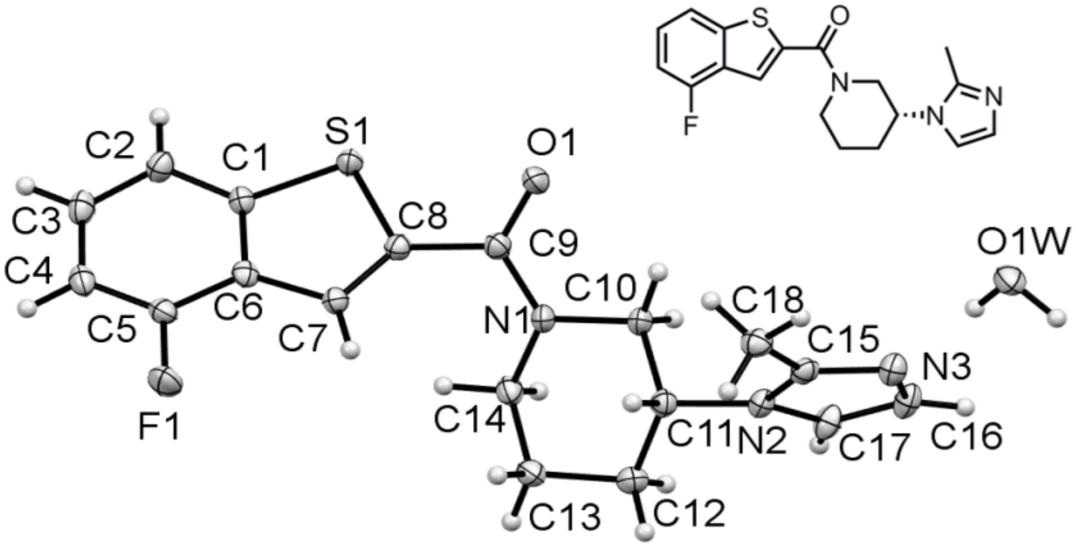

Figure S2. X-ray structure of compound 1 (*R*). The Oak Ridge Thermal Ellipsoid Plot (ORTEP) structure of (*R*)-(4-fluorobenzo[*b*]thiophen-2-yl)(3-(2-methyl-1*H*-imidazol-1-yl)piperidin-1-yl)methanone (compound **1** (*R*)). Please note, the crystal structure shows the presence of a water molecule indicating that the compound is a mono hydrate. Related to Figure **1**, table **1** and table **S1**.

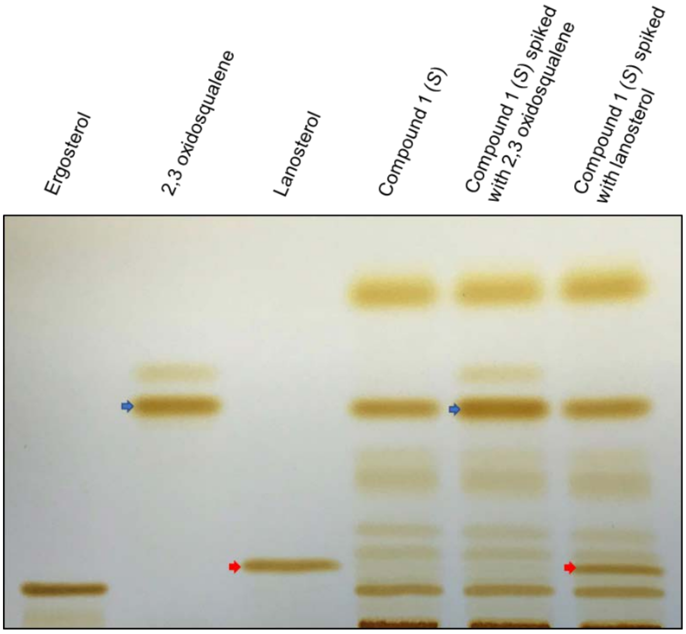

Figure S3. Effect of compound (*S*)-1 on the sterol biosynthesis in *L. donovani*. Sterols extracted from WT promastigotes treated with compound (*S*)-**1** (1.5 µM, 96 h) and identical samples spiked with 2,3-oxidosqualene (8 µg) were separated on HPTLC silica gel in heptane: ethyl ether: acetic acid buffer. Ergosterol, 2,3-oxidosqualene (blue arrows) and lanosterol (red arrows) standards (1 mg mL^{-1}) were run in parallel and the unsaturated double bonds of separated lipids were stained with iodine vapour. Related to figure **4**.

A

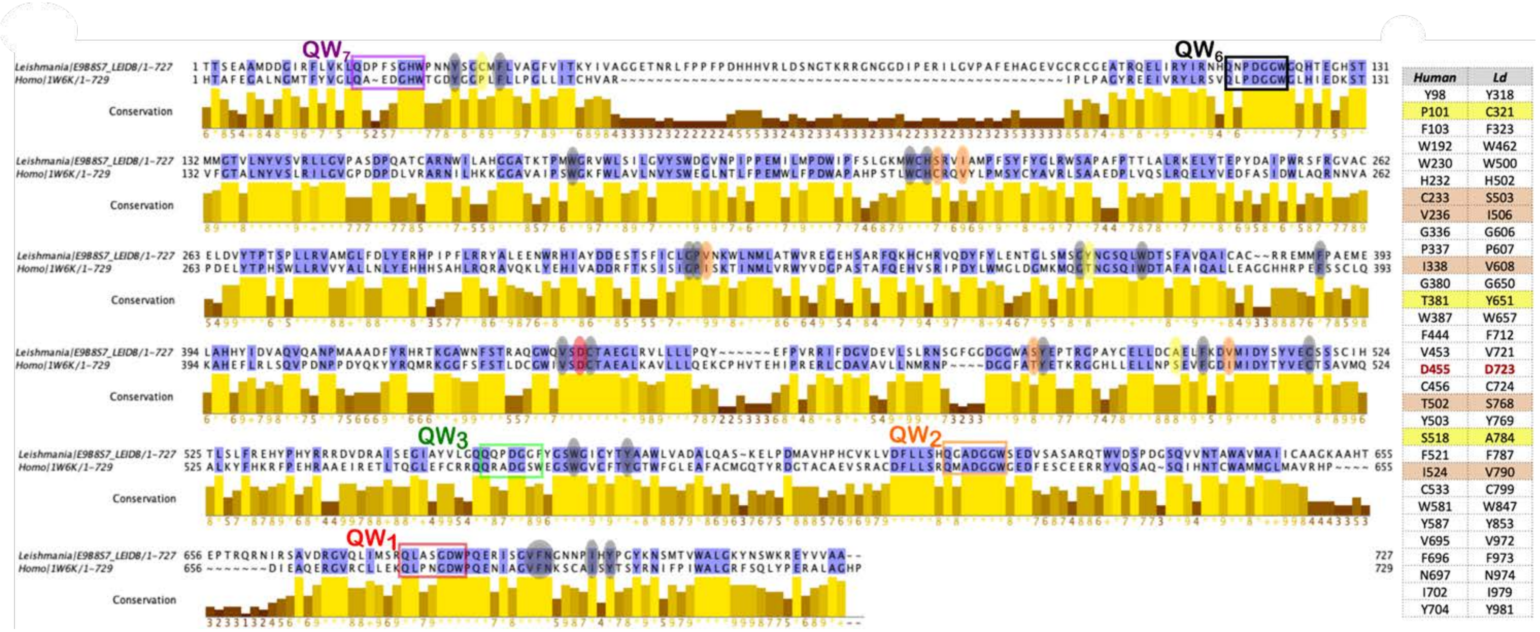

B

Human	*Ld*
Y98	Y318
P101	C321
F103	F323
W192	W462
W230	W500
H232	H502
C233	S503
V236	I506
G336	G606
P337	P607
I338	V608
G380	G650
T381	Y651
W387	W657
F444	F712
V453	V721
D455	D723
C456	C724
T502	S768
Y503	Y769
S518	A784
F521	F787
I524	V790
C533	C799
W581	W847
Y587	Y853
V695	V972
F696	F973
N697	N974
I702	I979
Y704	Y981

Figure S4. Edited alignment of the query sequence of *Ld*OSC and the template sequence of *h*OSC. (A) The alignment used to build the homology model of *Ld*OSC and the level of sequence conservation between the aligned residues. QW1-3 and QW6-7 motifs are highlighted red, orange green, black and purple, respectively. Identical residues are highlighted in blue. Residues that form the lanosterol binding site are highlighted with grey circles (identical), yellow circles (no match) and orange circles (residues with similar properties). The catalytic residue (Asp) is circled in red. In panel B, the residues of the binding site of *h*OSC and *Ld*OSC are listed. Related to figure **5**.

A

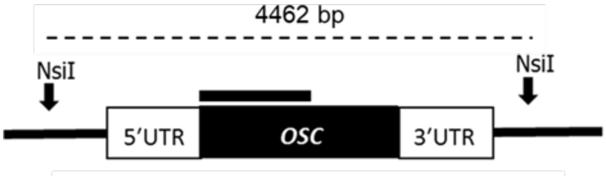

B

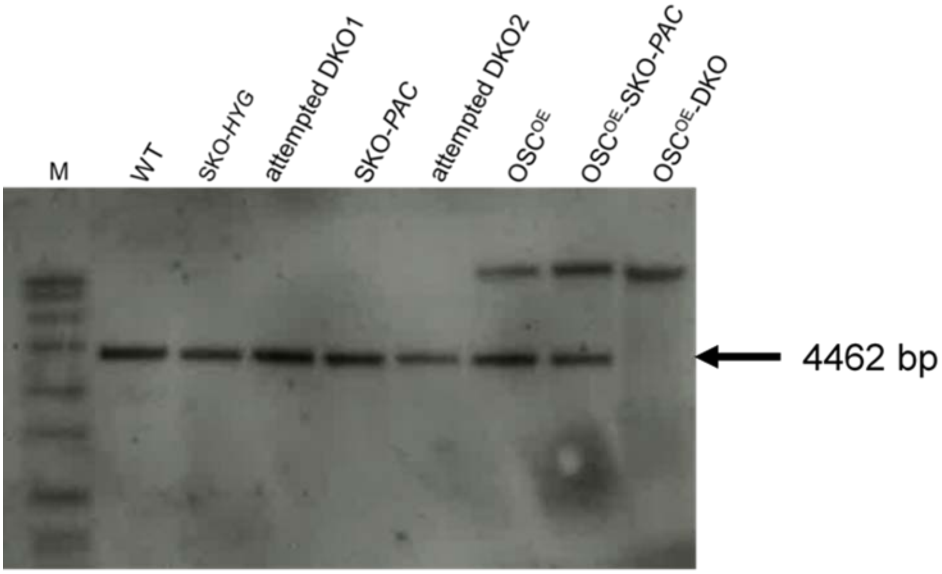

Figure S5. Assessing the essentiality of OSC in *L. donovani* promastigotes. (A) Schematic representation of the *OSC* locus. Black bars represent the region of the open reading frame of *OSC* that was DIG-labelled and used as a probe in Southern blot analysis. *Nsi*I sites with expected fragment sizes are shown. (B) Southern blot analysis of *Nsi*I-digested genomic DNA (~5 µg) from wild-type *L. donovani* (*Ld*BOB) (WT), *OSC*-single knockout clones (SKO-*HYG* and *PAC*), attempted double knockout cells (attempted DKO 1 and 2), OSC overexpressing cells (*OSC*^{OE}), and double knockout cells overexpressing OSC (*OSC*^{OE}-DKO). A DIG-labelled fragment of *OSC* was used as a probe. Related to figure **6**.

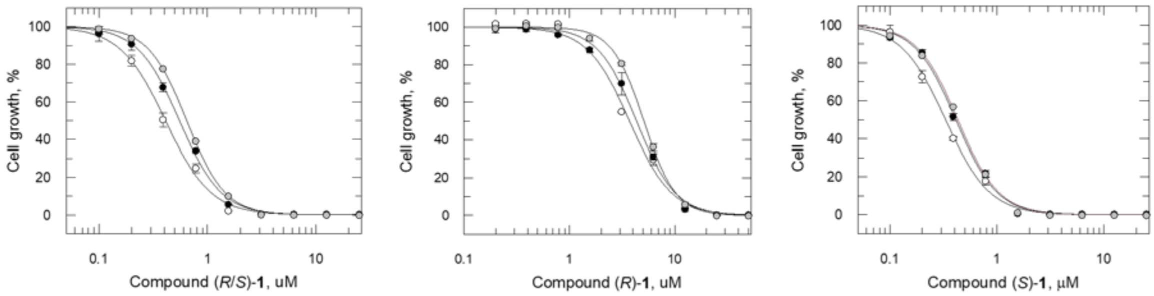

Figure S6. The effect of varying levels of FCS on the potency of compound 1 and enantiomers. Dose response curves with *L. donovani* promastigotes grown in culture media supplemented with 5, 10 or 20% FCS, respectively. For compound (*R*/*S*)-**1,** EC_{50} values of 0.4 ± 0.01, 0.55 ± 0.01 and 0.6 ± 0.008 µM were determined for parasites grown in 5, 10 and 20% FCS, respectively. For (*R*)-1, values of 3.7 ± 0.01, 4.3 ± 0.1 and 5 ± 0.08 µM were determined. Finally, for compound (*S*)-**1**, values of 0.3 ± 0.01, 0.41 ± 0.01 and 0.43 ± 0.01 µM were determined. All data represent mean ± SD of duplicate technical replicates. Related to table **1**.

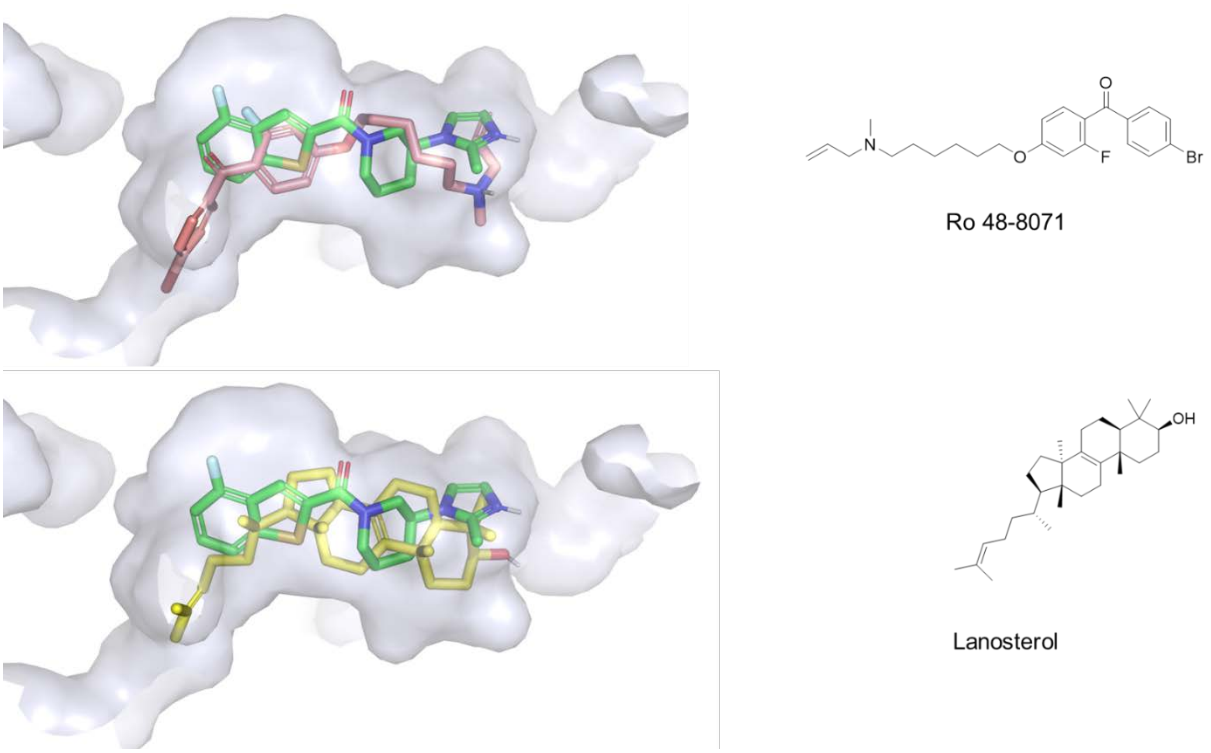

Figure S7. Comparison of substrate and inhibitor binding in the *Ld*OSC active site. Compound (*S*)-**1** binding in the *Ld*OSC homology model (best scoring pose) is shown in green. Binding of inhibitor *h*OSC inhibitor Ro 48-8071 (pink) and the substrate lanosterol (yellow) are superimposed. The molecular surface of the *Ld*OSC active site is shown in grey. Related to figure **5**.
